# Synthesis and evaluation of photoaffinity labeling reagents for identifying binding sites of sulfated neurosteroids on NMDA and GABA_A_ receptors†

**DOI:** 10.1039/d4ra07074g

**Published:** 2024-11-13

**Authors:** Mingxing Qian, Yuanjian Xu, Hong-Jin Shu, Zi-Wei Chen, Lei Wang, Charles F. Zorumski, Alex S. Evers, Steven Mennerick, Douglas F. Covey

**Affiliations:** a Department of Developmental Biology, Washington University in St. Louis 660 S. Euclid Ave. St. Louis MO 63110 USA dcovey@wustl.edu; b Department of Psychiatry, Washington University in St. Louis 660 S. Euclid Ave. St. Louis MO 63110 USA; c Department of Anesthesiology, Washington University in St. Louis 660 S. Euclid Ave. St. Louis MO 63110 USA; d Taylor Family Institute for Innovative Psychiatric Research, Washington University in St. Louis 660 S. Euclid Ave. St. Louis MO 63110 USA; e Department of Anesthesiology, Union Hospital, Tongji Medical College, Huazhong University of Science and Technology Wuhan 430022 China; f Key Laboratory of Anesthesiology and Resuscitation (Huazhong University of Science and Technology), Ministry of Education China

## Abstract

The endogenous neurosteroids dehydroepiandrosterone sulfate (DHEAS) and pregnenolone sulfate (PS) are allosteric modulators of γ-aminobutyric acid type A (GABA_A_) and *N*-methyl-d-aspartate (NMDA) type glutamate receptors. Analogues of these endogenous steroid sulfates can be either positive or negative allosteric modulators (PAMs or NAMs, respectively) of these receptors, but there is limited information about the steroid-protein binding interactions that mediate these effects and photoaffinity labeling reagents (PALs) of sulfated steroids have not been reported previously. The synthesis of a panel of ten sulfated steroid analogues containing a diazirine group, five of which also contain an alkyne group for click chemistry reactions, for use in photoaffinity labeling studies to identify binding sites for steroid sulfates that are either positive or negative allosteric modulators is reported. Electrophysiological measurements on cultured rat hippocampal neurons were made to determine the modes of allosteric modulation in comparison to those of PS on both receptors. PALs with the activity profile of PS (NMDA PAM, GABA_A_ NAM) were identified. Unexpectedly, PALs with PAM activity at both receptors were also found. Photolabeling of both receptors by two of the PALs was performed to demonstrate their utility, and by inference those of the other PALs, for future studies to identify binding sites for endogenous steroid sulfates on both receptors.

## Abstract 2

Photoaffinity labels (PALs) for sulfated steroid binding sites on GABA_A_ and NMDA receptors. The PALs have varying profiles of positive and negative modulatory (PAM and NAM) actions.

## Graphical abstract

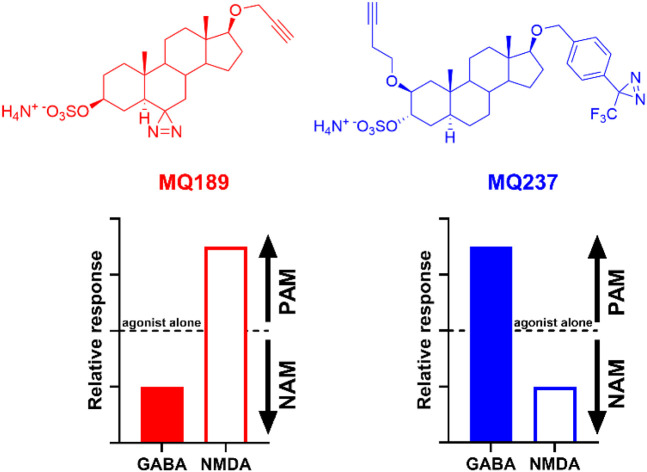

## Introduction

**Fig. 1:**
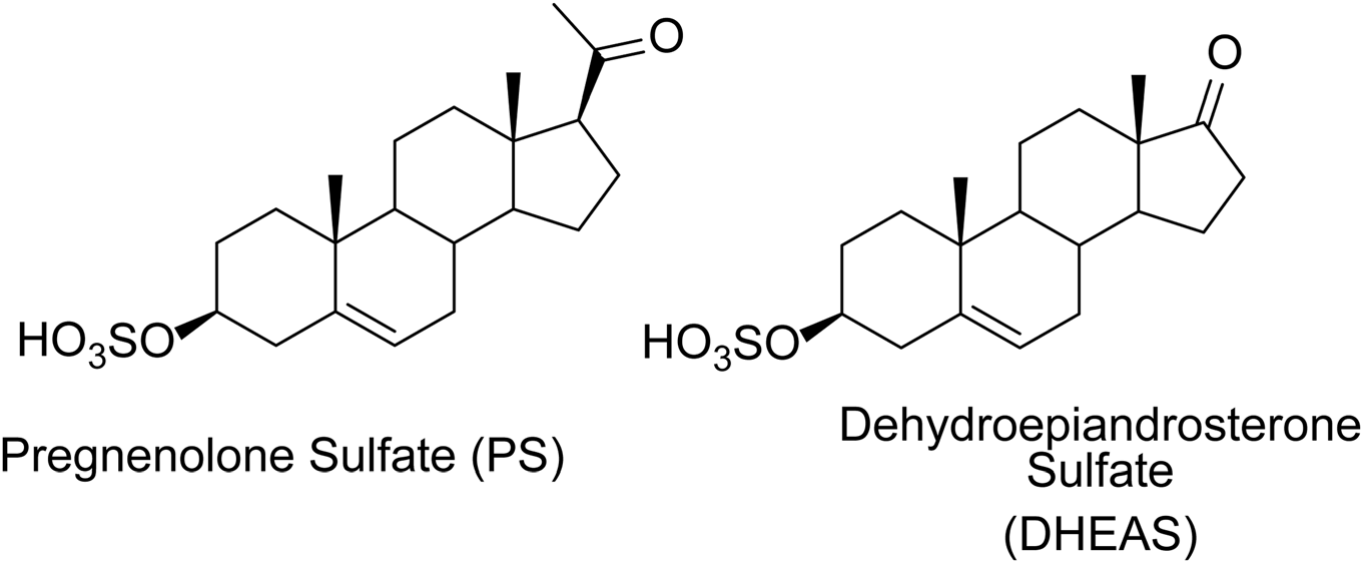
Structures of PS and DHEAS.

**Fig. 2:**
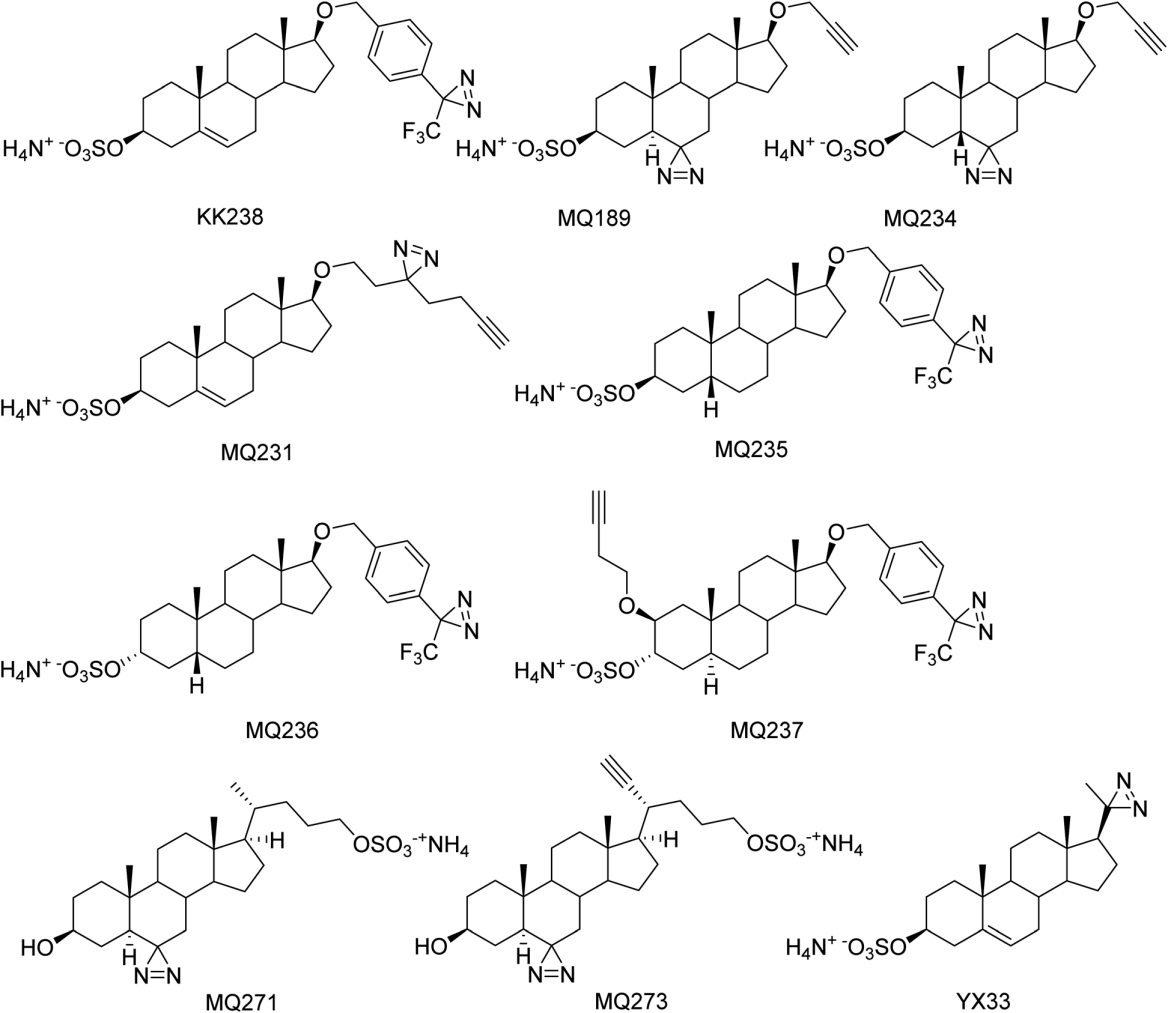
Structures of synthesized sulfated steroid PALs for photoaffinity labeling studies of NMDARs and GABA_A_Rs.

The endogenous steroids pregnenolone sulfate (PS) and dehydroepiandrosterone sulfate (DHEAS) (Fig. 1) are allosteric modulators of *N*-methyl-d-aspartate receptors (NMDARs) and γ-aminobutyric acid type-A receptors (GABA_A_Rs). PS is a positive allosteric modulator (PAM) of NMDARs containing GluN2A or GluN2B subunits and a negative allosteric modulator (NAM) of NMDARs containing GluN2C or GluN2D subunits.^1–3^ DHEAS is only a weak PAM at NMDAR.^4^ PS and DHEAS are NAMs at GABA_A_Rs.^5,6^ A cryo-EM study has shown binding of both PS and DHEAS in the pore of GABA_A_Rs.^7^ Additional information on modulation of these receptors by PS and DHEAS has been presented in recent reviews.^8,9^ Although site-directed mutagenesis and structural methods (cryo-EM) have been useful for gaining information on the locations of sulfated steroid binding sites on both receptors, another method for identifying these binding sites, photoaffinity labeling, has not been applied, and to our knowledge no photoaffinity labels (PALs) of sulfated steroid modulators of these receptors have been reported. Given limitations of sample preparation, overfitting, and other caveats of cryo-EM, we embarked on a complementary chemical biology effort. A similar effort has been instructive in delineating binding sites for non-sulfated steroid modulators of GABA_A_Rs.^10–13^ Herein, we report the synthesis of a panel of sulfated steroid PALs (Fig. 2). The PALs were screened using electrophysiological methods to determine their NAM and PAM actions at both NMDARs and GABA_A_Rs. Selected PALs were also shown to covalently modify NMDARs or GABA_A_Rs. Of particular interest was the finding that three PALs containing the trifluromethylphenyl diazirine (TPD) photolabeling group (MQ235, MQ236, MQ237) had the novel profile of NAM activity at NMDARs and PAM activity at GABA_A_Rs. Another compound containing the TPD photolabeling group (KK238), an analogue of PS, had the novel profile of PAM activity at both receptors.

## Results and discussion

### Chemistry

**Scheme 1:**
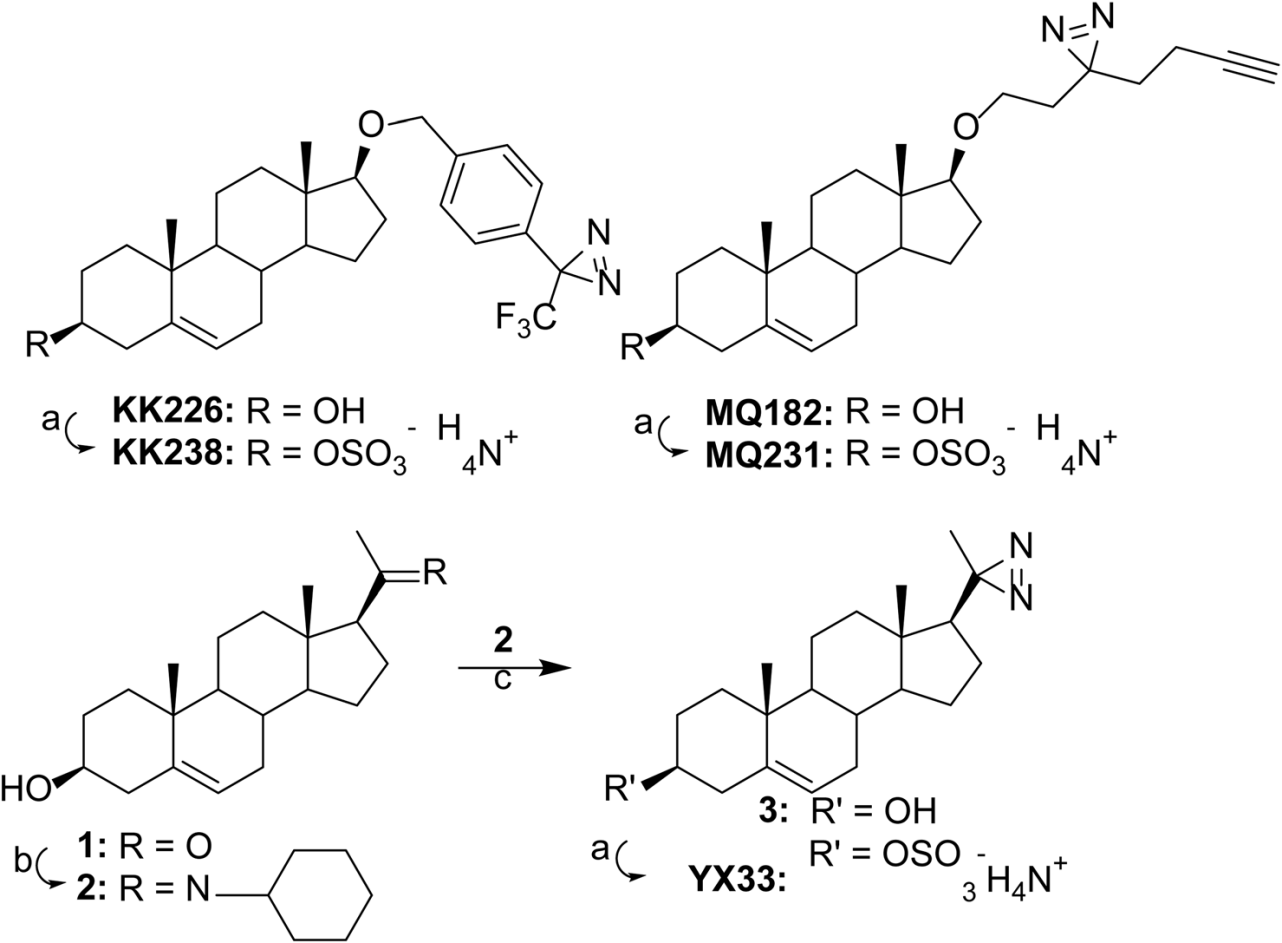
Synthesis of KK238, MQ231 and YX33. Reagents: (a) (1) SO_3_–Me_3_N, pyridine, 23 °C, 15 h; (2) 7 N NH_3_ in MeOH, (KK238, 99%), (MQ231, 64%), (YX33, 47%); (b) CF_3_CO_2_H, cyclohexylamine, reflux under N_2_, 15 h; (c) (1) NH_2_OSO_3_H, NH_3_, MeOH, 23 °C, 72 h; (2) NH_3_, I_2_, 0 °C then 23 °C, 20 min; (3) SO_3_–Me_3_N, pyridine, 23 °C, 15 h; (4) 7 N NH_3_ in MeOH, (19%, from 1).

The syntheses of KK238, MQ231 and YX33 are shown in Scheme 1. KK238 and MQ231 were prepared by sulfation of the previously reported steroids KK226 (ref. 14) and MQ182 (ref. 15) followed by conversion to their ammonium salts. YX33 was prepared from pregnenolone (1) by reaction with cyclohexylamine to yield intermediate enamine 2 which was then converted to diazirine 3 and then similarly sulfated and converted to its ammonium salt to yield YX33.

**Scheme 2:**
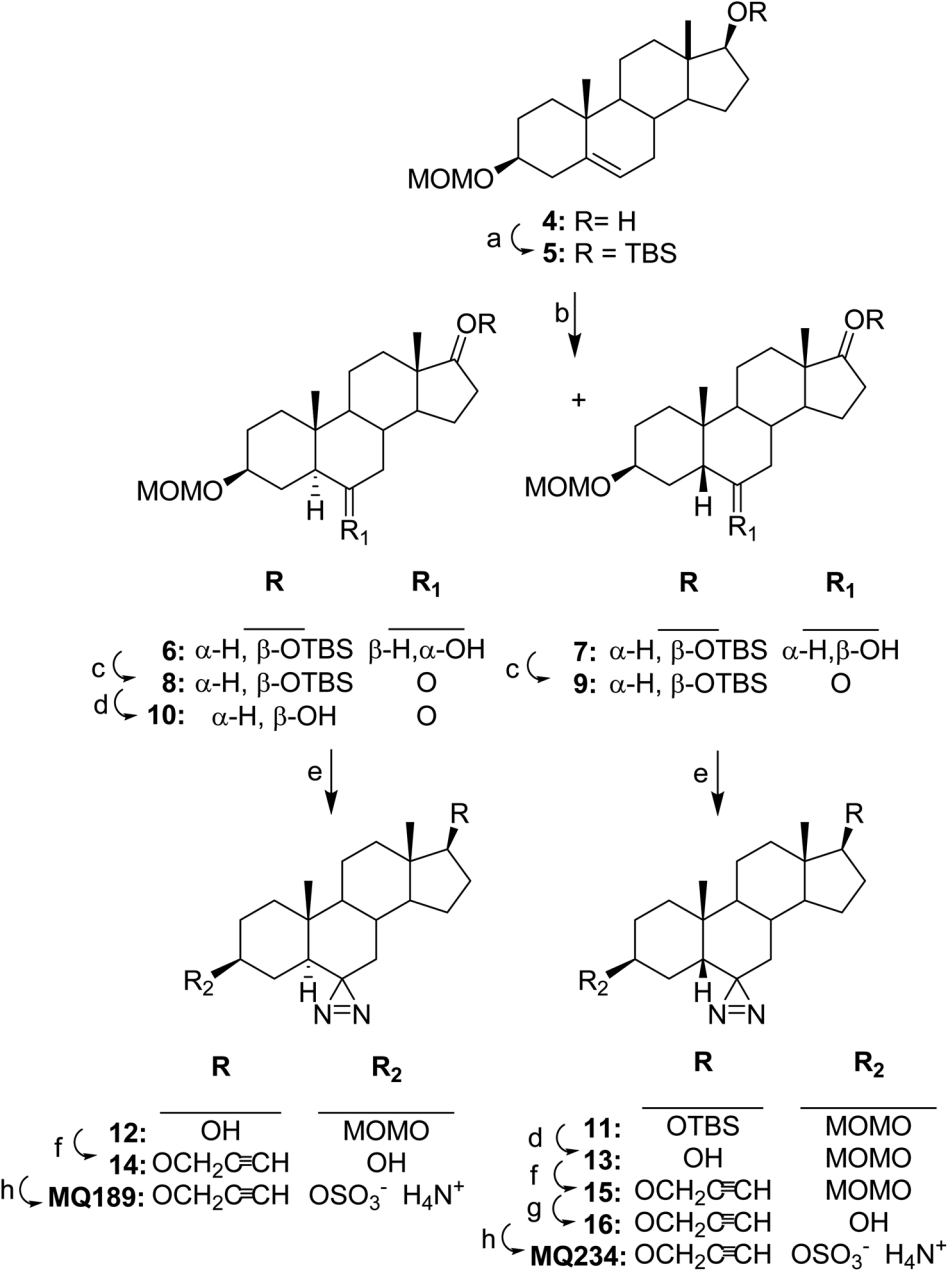
Synthesis of MQ189 and MQ234. Reagents, conditions and yields: (a) TBSCl, imidazole, DMF, 23 °C, 16 h, (96%); (b) (1) BH_3_-THF, 23 °C, 2 h, (2) 3 N NaOH, 23 °C, 1 h, (6, 73% + 7, 13%); (c) Dess–Martin reagent, NaHCO_3_, 23 °C, 1 h, (8, 88%), (9, 98%); (d) TBAF, THF, 23 °C, 16 h, (8 → 10, 96%), (11 → 13, 74%); (e) (1) 7 N NH_3_ in MeOH, 23 °C, 24 h followed by NH_2_OSO_3_H, 23 °C, 72 h; (2) NH_3_, I_2_, 23 °C, 20 min, (9 → 11, 4% + 12, 43%), (10 → 12, 86%); (f) (1) NaH, THF, reflux, 1 h; (2) propargyl bromide in toluene, reflux 16 h, (13 → 15, 83%), (12 → 14, 62% after removal of MOM group as described for steroid 16 in following reaction (g)); (g) 6 N HCl, THF, 23 °C, 2 h, (16, 74%); (h) (1) SO_3_–Me_3_N, pyridine, 23 °C, 15 h; (2) 7 N NH_3_ in MeOH, (MQ189, 89%), (MQ234, 93%).

The syntheses of MQ189 and MQ234 are described in Scheme 2. The hydroxyl group of previously reported steroid 4 (ref. 15) was protected to yield steroid 5 which was then hydroborated to give a mixture of steroids 6 and 7 which were separated by flash column chromatography. The newly formed 6-hydroxyl group in separated steroids 6 and 7 was oxidized to yield 6-ketosteroids 8 and 9, respectively. The hydroxyl protecting group of steroid 8 was then removed to obtain steroid 10 which was then converted by a two-step procedure to diazirine 12. Propargylation of the 17-hydroxyl group of steroid 12 with subsequent hydrolysis of the hydroxyl protecting group gave propargylated steroid 14. Sulfation and salt formation gave MQ189.

Using a similar, but slightly different reaction sequence steroid 9 was converted to diazirine 11 and then the hydroxyl protecting group was removed to yield steroid 13. Propargylation as described for the preparation of steroid 14 converted steroid 13 to steroid 15. Hydrolysis of the hydroxyl protecting group in steroid 15 yielded steroid 16 and then sulfation and salt formation converted steroid 16 to MQ234.

**Scheme 3:**
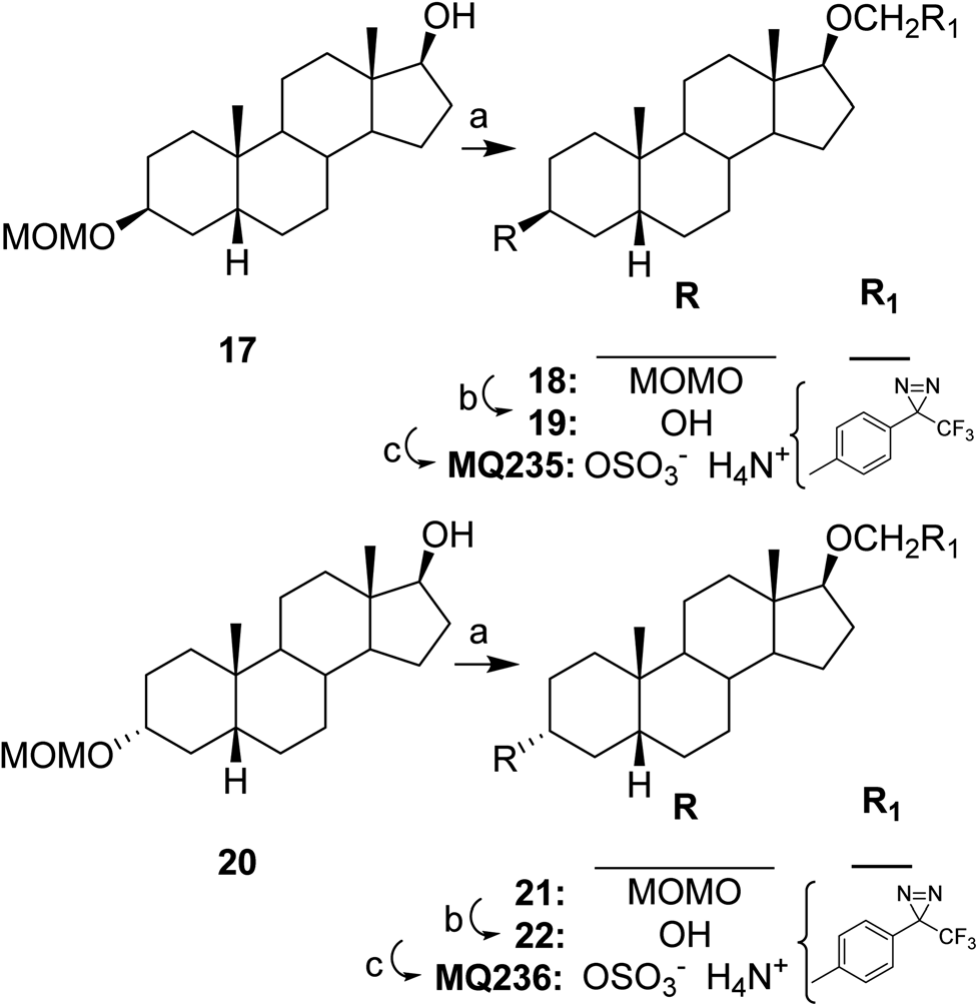
Syntheses of MQ235 and MQ236. Reagents, conditions and yields: (a) 3-[4-(Iodomethyl)phenyl]-3-(trifluoromethyl)-3*H*-diazirine, Bu_4_NI, NaH, THF, reflux 16 h, (18, 59%), (21, 63%); (b) 6 N HCl, THF, 23 °C, 2 h, (19, 57%), (22, 91%); (c) (1) SO_3_–Me_3_N, pyridine, 23 °C, 15 h; (2) 7 N NH_3_ in MeOH, (MQ235, 74%), (MQ236, 93%).

The syntheses of MQ235 and MQ236 are described in Scheme 3. The TPD group was attached to the hydroxyl group of previously reported steroid 17 (ref. 16) to give steroid 18. Hydrolysis of the hydroxyl protecting group in steroid 18 converted it to steroid 19 and sulfation followed by salt formation converted steroid 19 to MQ235. A similar reaction sequence (20 → 21 → 22 → MQ236) converted previously reported steroid 20 (ref. 17) to MQ236.

**Scheme 4:**
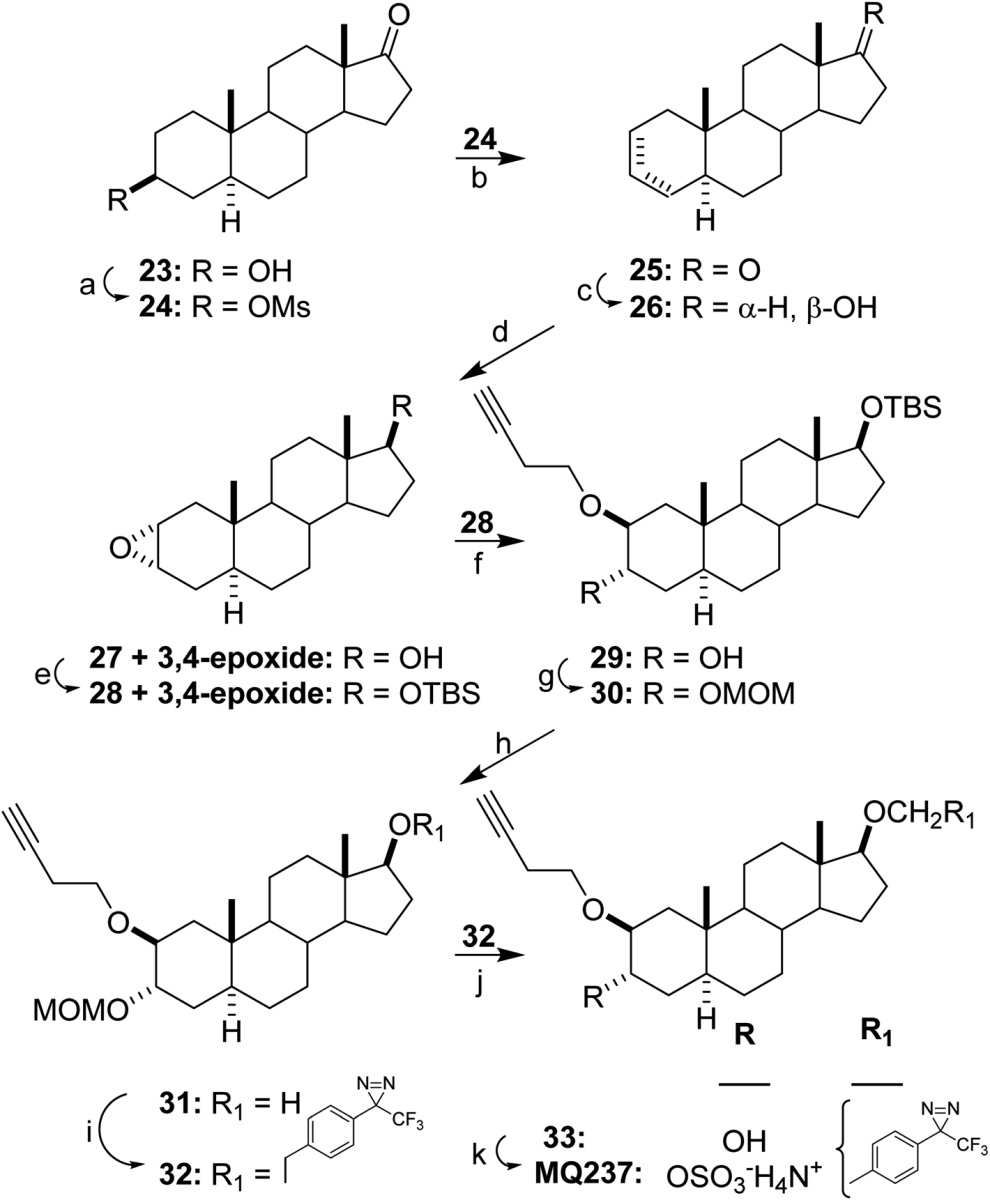
Synthesis of MQ237. Reagents, conditions and yields: (a) MsCl, Et_3_N, CH_2_Cl_2_, 0 °C, 2 h, (98%); (b) LiBr, DMF, 130 °C, 90 min, (91%); (c) NaBH_4_, EtOH, 23 °C, 1 h, (95%); (d) HCO_2_H, 30% H_2_O_2_, 23 °C, 2 h, (81%); (e) TBSCl, imidazole, DMF, 23 °C, 16 h, (86%); (f) C_2_(CN)_4_, 3-butyn-1-ol, 23 °C, 72 h, (44%); (g) ClCH_2_OMe, (i-Pr)_2_NEt, CH_2_Cl_2_, 23 °C, 16 h, (78%); (h) TBAF, THF, 23 °C, 16 h, (71%); (i) 3-[4-(Iodomethyl)phenyl]-3-(trifluoromethyl)-3*H*-diazirine, Bu_4_NI, NaH, THF, reflux 16 h, (52%); (j) 6 N HCl, THF, 23 °C, 2 h, (68%); (k) (1) SO_3_–Me_3_N, pyridine, 23 °C, 15 h; (2) 7 N NH_3_ in MeOH, (MQ237, 30%).

The synthesis of MQ237 is described in Scheme 4. The hydroxyl group of epiandrosterone (23) was converted into mesylate 24 and then steroid 24 was converted into an inseparable mixture of the Δ^2^ and Δ^3^ (5 : 1 ratio) steroids 25. The 17-ketone group of steroid mixture 25 was reduced to give steroid mixture 26 which was then converted into epoxide mixture 27. The hydroxyl groups of epoxy steroid mixture 27 were protected to obtain steroid mixture 28 and then the epoxides were opened using 3-butyn-1-ol in tetracyanoethylene to give a mixture of products from which pure steroid 29 was obtained after flash column chromatography. Protection of the hydroxyl group of steroid 29 gave steroid 30 and removal of the hydroxyl protecting group from steroid 30 gave steroid 31. Using reactions already shown in Scheme 3 for addition of the TPD group, *i.e.*, removal of the hydroxyl protecting group, sulfation and salt formation; the reaction sequence 31 → 32 → 33 → MQ237 completed the synthesis of MQ237.

**Scheme 5:**
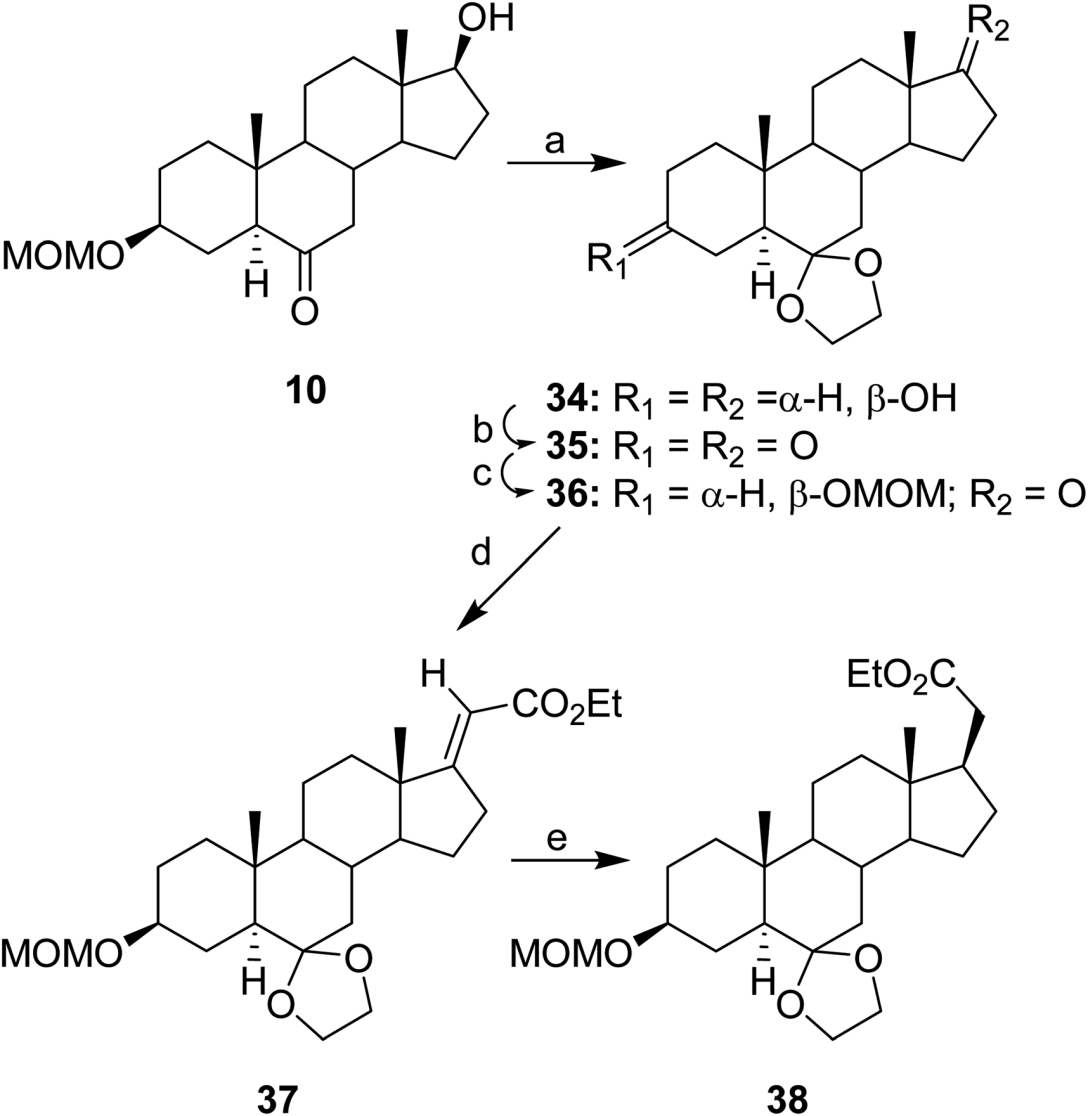
Initial steps in synthesis of MQ271 and MQ273. (a) HO(CH_2_)_2_OH, benzene, PTSA, reflux, 16 h, (57%); (b) Dess–Martin reagent, NaHCO_3_, 23 °C, 1 h, (98%); (c) (1) Li(*t*-BuO)_3_AlH, THF, −40 °C, 1 h; (2) ClCH_2_OMe, (i-Pr)_2_NEt, CH_2_Cl_2_, 23 °C, 16 h, (84%, 2 steps); (d) triethyl phosphonoacetate, EtOH, NaOEt, under N_2_, reflux 16 h, (74%); (e) H_2_, PtO_2_, EtOH, 23 °C, 3 h, (87%).

**Scheme 6:**
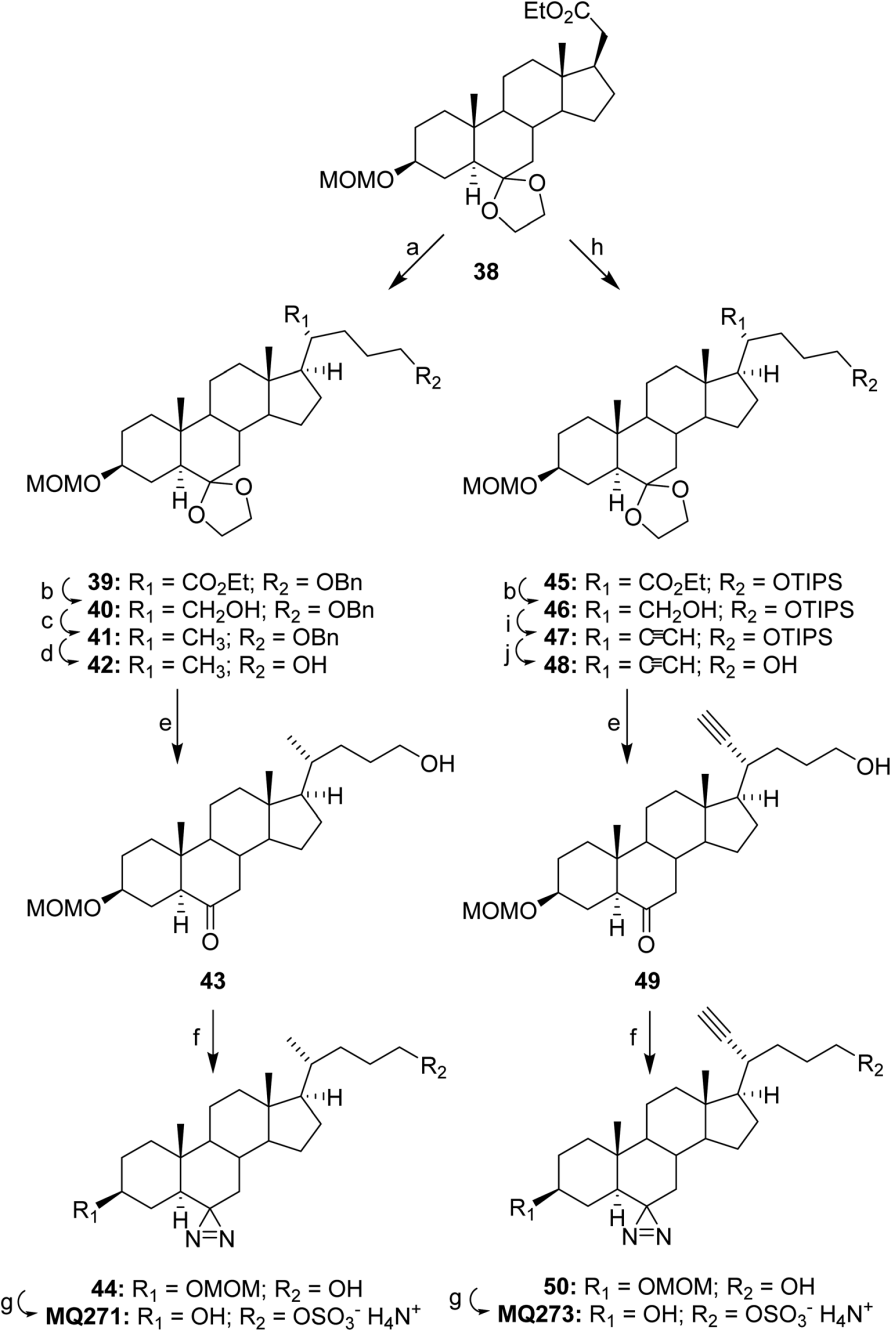
Final steps in synthesis of MQ272 and MQ273. Reagents: (a) ((3-iodopropoxy)methyl)benzene, LDA, HMPA, THF, −78 °C, 45 min then 23 °C, 16 h, (98%); (b) LiAlH_4_, Et_2_O, 23 °C, 2 h, (40, 82%; 46, 96%); (c) (1) MsCl, Et_3_N, CH_2_Cl_2,_ 0 °C 2 h; (2) LiAlH_4_, Et_2_O, 45 min, 23 °C, (97%); (d) Pd/C, H_2_ (50 psi), EtOAc, 1 h, (93%); (e) PTSA, acetone/H_2_O, 23 °C, 3 h, (43, 84%; 49, 96%); (f) (1) NH_2_OSO_3_H, NH_3_, MeOH, 23 °C, 72 h; (2) NH_3_, I_2_, 0 °C then 23 °C, 20 min, (44, 84%; 50, 73%); (g) (1) SO_3_–Me_3_N, pyridine, 23 °C, 15 h; (2) 6 N HCl, THF, 23 °C, 2 h; (3) 7 N NH_3_ in MeOH, (MQ271, 79%; MQ273, 83%); (h) (3-iodopropoxy)(triisopropyl)silane, LDA, HMPA, THF, −78 °C then 23 °C, 16 h, (95%) (i) (1) Dess–Martin reagent, NaHCO_3_, 23 °C, 2 h; (2) (dimethyl-1-diazo-2-oxopropyl)phosphonate, K_2_CO_3_, MeOH/THF, 23 °C, 48 h, (64%); (j) TBAF, THF, 23 °C, 4 h, (∼100%).

The initial steps for the synthesis of MQ271 and MQ273 are shown in Scheme 5 and the final steps are shown in Scheme 6. In Scheme 5, the ketone group of steroid 10 was protected as a cyclic ketal to give steroid 34 and oxidation of the hydroxyl group of steroid 34 gave steroid 35. The 3-ketone group of steroid 35 was selectively reduced and the resultant 3-hydroxyl group was subsequently protected to obtain steroid 36. A previously reported procedure was used to introduce a side chain at the C17 position with the required sterochemisty.^18^ Thus, a Wittig reaction converted steroid 36 to steroid 37 and subsequent hydrogenation converted steroid 37 to steroid 38.

In Scheme 6, alkylation of steroid 38 with ((3-iodopropoxy)methyl)benzene converted steroid 38 to steroid 39 and reduction of its carboethoxy group yielded steroid 40. Mesylation of steroid 40 followed by displacement of the mesylate gave steroid 41 and hydrogenation of steroid 41 yielded steroid 42. Removal of the ketal group in steroid 42 gave steroid 43. The ketone group of steroid 43 was then converted to the diazirine group in steroid 44. Protecting group removal followed by sulfation and salt formation converted steroid 44 to MQ271.

In Scheme 6, alkylation of steroid 38 with (3-iodopropoxy)(triisopropyl)silane converted steroid 38 to steroid 45 and reduction of its carboethoxy group yielded steroid 46. Oxidation of the hydroxyl group of steroid 46 gave an intermediate aldehyde that was immediately converted to the alkyne containing steroid 47. Removal of the TIPS protecting group converted steroid 47 to steroid 48 and removal of the ketal group in steroid 48 converted this compound to steroid 49. The ketone group of steroid 43 was then converted to the diazirine group in steroid 50 and removal of the hydroxyl protecting group, sulfation and salt formation converted steroid 44 to MQ273.

### Electrophysiologic evaluation

**Fig. 3:**
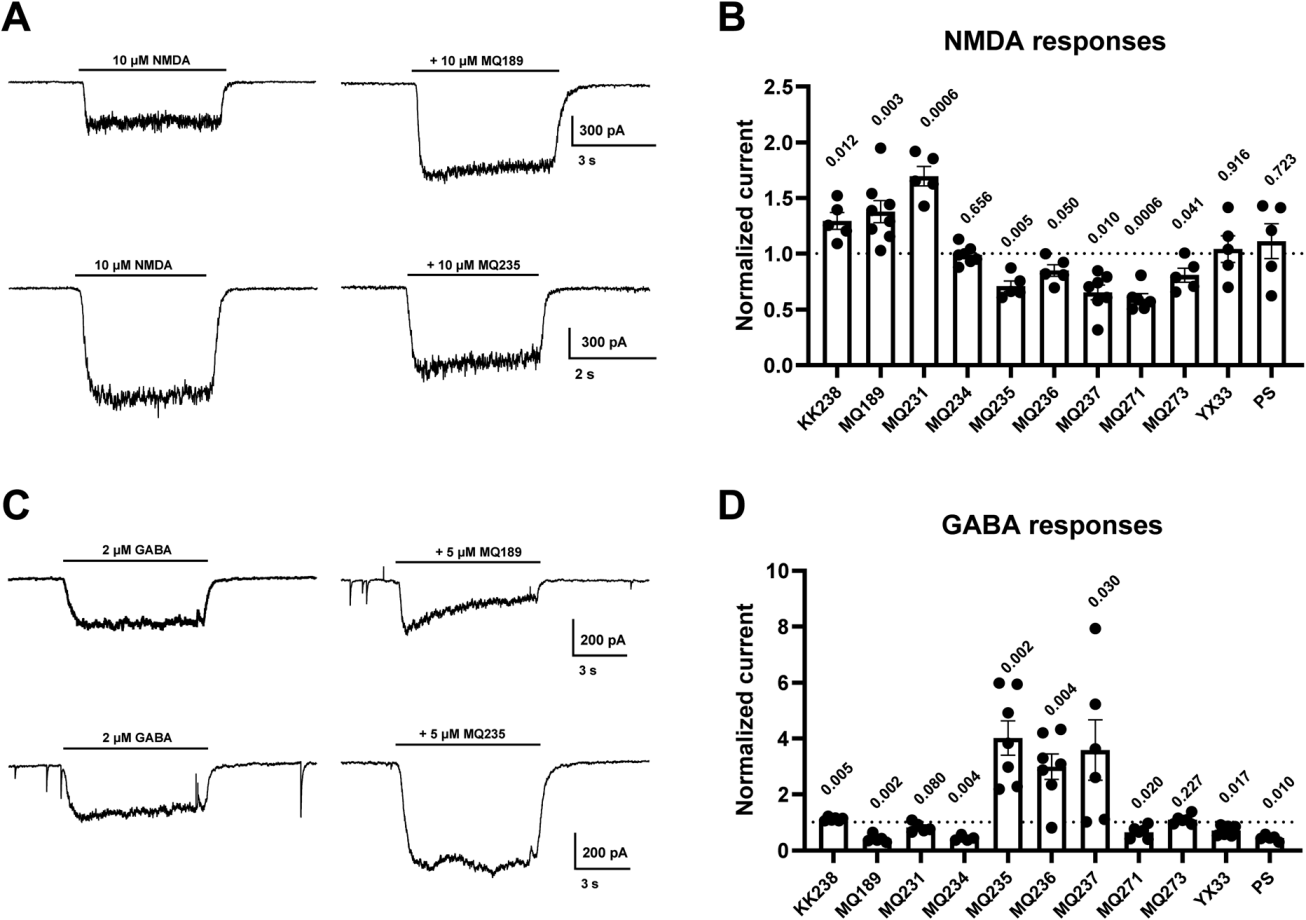
Electrophysiological activity of PAL reagents at NMDARs and GABA_A_Rs. (A) Representative traces of neuroactive steroid analogues that potentiate (top) and inhibit (bottom) responses to 10 μM NMDA elicited from rat hippocampal neurons. Left panels show responses from two different neurons to NMDA alone; each NMDA response is matched to a response from the same cell to co-application of NMDA plus 10 μM modulator (right panels). Note that MQ189 potentiated and MQ235 inhibited the response to NMDA. (B) Summary of multiple sulfated neurosteroids screened on NMDA responses (*n* = 3–5). The dashed line illustrates the initial NMDA response, to which co-application responses were normalized. (C) The same paradigm was used to examine modulation of GABA_A_R responses. Left panels are representative traces of the response to 2 μM GABA elicited from two different rat hippocampal neurons. This response was inhibited by co-application of 5 μM MQ189 at the steady-state (upper panel). In contrast, the GABA response was enhanced by co-application of 5 μM MQ235 (lower panel). (D) Summary of sulfated neurosteroids screened for GABA_A_R modulation (*n* = 5). The dashed line illustrates the initial GABA response to which co-application responses were normalized. The *p* values for SEM differences from control are shown above each compound.

To determine the allosteric mode of action of the PAL reagents the compounds were evaluated using electrophysiological methods on cultured rat hippocampal neurons. The PAL reagents were screened at 10 μM at NMDARs and at 5 μM at GABA_A_Rs (Fig. 3). The lower concentration for the GABA_A_R determinations was chosen to avoid saturation (complete inhibition) observed by some of the PALs at 10 μM and thereby facilitate meaningful comparisons. We were also interested in how test compounds compared with the published reference sulfated neurosteroid PS. NMDA and GABA agonist concentrations were set below EC_50_ to facilitate detection of either inhibition or potentiation. The screening protocol was designed to compare activity across compounds on either receptor class at equimolar modulator concentration but not to identify differences in PAL potency or efficacy. Future photolabeling studies of either receptor will require additional dose–response determinations. At 10 μM, and based on a criterion of a ratio of *t* test *p* value < 0.05, five PALs (MQ235, MQ236, MQ237, MQ271, MQ273) have NMDAR NAM activity, three PALs (KK238, MQ189, MQ231) have PAM activity and two PALs do not have significant NAM or PAM activity (MQ234, YX33). At 5 μM, six PALs have GABA_A_R NAM activity (MQ189, MQ231, MQ234, MQ271, MQ273, YX33), three compounds have clear PAM activity (MQ235, MQ236, MQ237) and KK238 has slight but statistically consistent GABA_A_R PAM activity. The control comparator PS was reported previously to have NMDAR PAM activity and GABA_A_R NAM activity.^4,5^ At the screening concentration of 10 μM, PS NMDAR activity was expected to be at the threshold of biological activity; also expected, the GABA_A_ NAM activity was more potent.^4^MQ234 and YX33 may also have the PS activity profile as each has the same PS activity at one of the two receptors, but not the PS activity at the other receptor under the chosen screening conditions. In general, GABA_A_R NAM activity of neurosteroids exhibit apparent greater potency than either effect on NMDARs.^4^ Therefore, actions of MQ231, with little or no GABA_A_R activity but strong NMDAR activity, or like MQ237, with potentiating GABA_A_ and robust NMDAR NAM activity, may be particularly surprising.

MQ231, YX33 and KK238, like PS, have a 5,6-double bond and the 3β-sulfate group. MQ231 has the same reported activity profile as PS even though the substituents at the C-17 position on the steroid D-ring are different. The lack of significant activity at the screening concentrations chosen in this study makes it impossible to conclude that YX33 has the PS activity profile. However, the similarity of the C-17 substituent in YX33 to that of it in PS suggests that their activity profiles are similar. By contrast, the C-17 substituent (TPD group) in KK238 is the same as that of MQ235, MQ236 and MQ237 and these four compounds do not have the PS activity profile, as unlike PS, these PALs are GABA_A_R PAMs. In results not shown, KK238 also was found to have greater GABA_A_R PAM activity when evaluated at 10 μM (71 ± 7% increase, *n* = 4) thereby confirming the statistical results shown in Fig. 3. It appears that the TPD group is responsible for the GABA_A_R PAM activity since this activity is found regardless of other structural differences in the four PALs. The finding that these four steroids have GABA_A_R PAM activity indicates that unlike PS and DHEAS these analogues are unlikely to bind in the ion channel pore as previously reported in a cryo-EM study^7^ because binding within the ion channel would be expected to produce NAM, not PAM, activity.

MQ271 and MQ273 have the sulfate group in the C-17 side chain. As screened, both are NMDAR NAMs but only MQ271 showed GABA_A_R NAM activity at 5 μM. In a previous study, we reported that a series of non-photolabeling androstane analogues containing a sulfate group in the same 17β-side-chain found in MQ271 and MQ273 had a variety of actions at GABA_A_Rs and NMDARs depending on the stereochemistry at the steroid 3 and 5 positions.^19^ One of those analogues (MQ221) was a GABA_A_R PAM and NMDAR NAM. Since MQ221 does not contain the 17β-TPD group found in KK238, MQ235, MQ236 and MQ237, it may be possible that different PAL analogues of MQ271 and MQ273 could also have GABA_A_R PAM and NMDAR NAM activity. The binding orientation of steroids having the sulfate group in the 17β-side-chain may be opposite to that of the C-3 sulfated steroid modulators of these receptors and this may explain why GABA_A_R PAM and NMDAR NAM activity exists for both series of sulfated steroids.

### Photolabeling of GABA_A_Rs and NMDARs

**Fig. 4:**
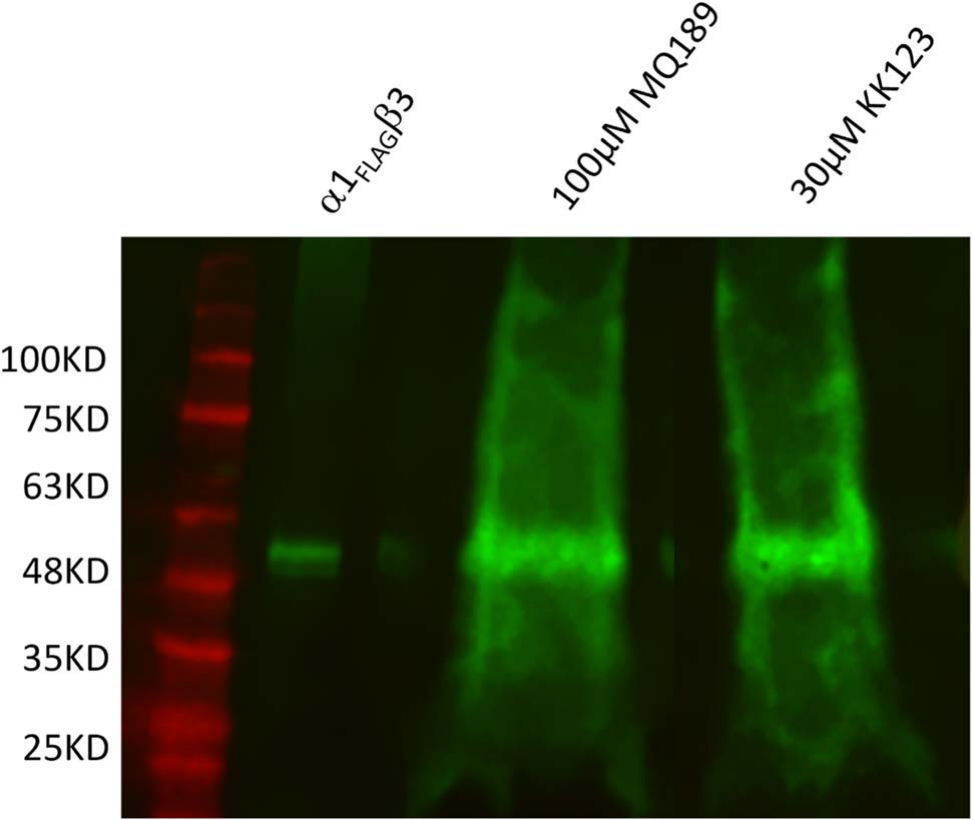
Enrichment of α1_FLAG_β3 GABA_A_Rs following photolabeling with MQ189, a sulfated NAM neurosteroid PAL, and KK123, a non-sulfated PAM neurosteroid PAL. The alkyne moiety of the covalently attached PAL reagent was coupled to a cleavable biotin-azide linker MQ112 using click chemistry. Photolabeled receptors were then enriched on streptavidin-agarose beads and eluted by cleavage of the linker. The figure shows an anti-FLAG-western demonstrating enrichment of photolabeled GABA_A_Rs. Lane 1 lysate of membranes expressing α1_FLAG_β3 GABA_A_Rs and shows the mass (∼52 kDa) of the α1_FLAG_ subunit. The bands at 52 kDa indicates that both MQ189 (Lane 2) and KK123 (Lane 3) photolabel the α1_FLAG_ subunit of the GABA_A_R.

Having determined the activity profiles of the PAL reagents we next selected two of them for a preliminary study of receptor photolabeling. To determine if the sulfated neurosteroid analogue PAL reagents labeled GABA_A_ receptors, we used MQ189, a GABA_A_ NAM containing a diazirine moiety for photolabeling and an alkyne that could be used for enrichment of photolabeled proteins. Membranes containing human 8x His-α1_FLAG_β_3_ GABA_A_Rs were photolabeled with either MQ189 or as a positive control, KK123, a non-sulfated neurosteroid PAL previously shown to label the α1 and β3 subunits of GABA_A_Rs.^10^ Photolabeled proteins in the membranes were solubilized, clicked to a cleavable biotin-azide linker, immobilized on streptavidin beads and eluted by chemical cleavage of the linker. Photolabeled GABA_A_Rs were then identified by the FLAG-tag on the α1 subunit. Fig. 4 shows a western blot illustrating that the α1 subunit of the GABA_A_R was identified as a ∼53 kDa band and that MQ189- and KK123-labeled FLAG-tagged proteins of the same molecular weight. These data indicate that MQ189 (100 μM) and KK123 (30 μM) label the α1 subunit of GABA_A_Rs to a similar extent.

**Fig. 5:**
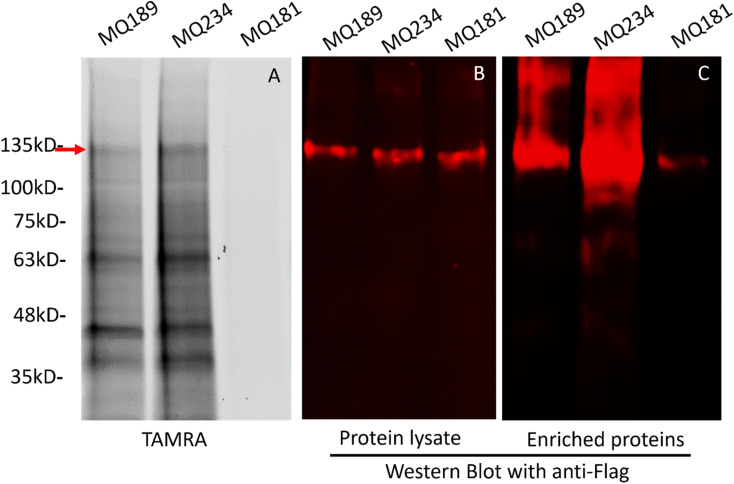
Photolabeling of GluN1_FLAG_/GluN2b NMDAR by MQ189 (a PAM PAL analogue) and MQ234 (a neutral NAM PAL analogue at the screening concentration). Membranes containing GluN1_FLAG_/GluN2b NMDA receptors were photolabeled with either MQ189, MQ234 or MQ181 [(3β,17β)-17-ethoxyandrost-5-en-3ol, 3-sulfate ammonium salt, a non-diazirine containing NMDAR PAM analogue] and lysed in SDS. Photolabeled receptors were then coupled *via* click chemistry to either TAMRA (a fluorescent dye; panel A) or to a cleavable biotin-azide linker (panels B and C). (A) Fluorescent image of an SDS-PAGE gel showing TAMRA-coupled photolabeled proteins. MQ234 labeled multiple proteins including a band at ∼135 kDA (arrow) corresponding to the GluN1_FLAG_ subunit. No TAMRA staining was observed with MQ181 indicating that the diazirine is required for photolabeling. (B) Anti-FLAG western blot of lysate of photolabeled membranes showing that the MQ189, MQ234 and MQ181 lysates contain equal amounts of GluN1_FLAG_ subunit. (C) Photolabeled receptors were enriched on streptavidin-agarose beads, eluted by cleavage of the linker and analyzed by SDS-PAGE and anti-FLAG western blot. MQ189- and MQ234-photolabeled GluN1_FLAG_ subunits were strongly enriched, whereas no enrichment was observed with MQ181.

To determine if the sulfated neurosteroid analogue PAL reagents labeled NMDARs, we used two sulfated neurosteroid PAL reagents, MQ189 and MQ234, both containing a diazirine photolabeling group and an alkyne for enrichment but differing by their stereochemistry at C5. MQ189 is an NMDAR PAM and MQ234 has neither NMDAR NAM nor PAM activity under the chosen screening concentrations. As a negative control, we also used MQ181 [(3β,17β)-17-ethoxyandrost-5-en-3-ol, 3-sulfate, ammonium salt], a sulfated neurosteroid that acts as an NMDAR PAM but contains neither a diazirine nor an alkyne moiety. We photolabeled membranes containing GluN1_FLAG_/GluN2b NMDA receptors with either 15 μM MQ189, MQ234 or MQ181. The photolabeled proteins were then attached to either TAMRA dye or to MQ112,^10^ a cleavable biotin-azide linker molecule, using a cycloaddition reaction. TAMRA-labeled proteins were analyzed by SDS-PAGE with fluorescent imaging. The fluorescent images show that MQ189 and MQ234 label multiple protein bands, with similar but non-identical labeling patterns. Both reagents label a band at ∼135 kDa (red arrow) with the approximate mass of the GluN1_FLAG_ subunit. No photolabeled proteins were observed with MQ181 due to its lack of the functional groups required for photolabeling and cycloaddition (Fig. 5A). The MQ112-coupled proteins were analyzed by western blot with anti-FLAG antibody. The protein lysate (prior to enrichment) shows that GluN1_FLAG_ migrates to the same location as the TAMRA-labeled 135 kDa protein (red arrow in Fig. 5A), and that each of the labeled samples contain an equivalent amount of GLuN1_FLAG_ subunit. Following enrichment on streptavidin beads, the GluN1_FLAG_ band is observed for the MQ189 and MQ234, but not the MQ181 labeled samples (Fig. 5C). These data indicate that MQ189 and MQ234 photolabel the GluN1_FLAG_ subunit of the NMDAR.

### Conclusions and future directions

In conclusion, we report the synthesis and electrophysiological evaluation for a panel of sulfated steroid PALs for photolabeling of NMDARs and GABA_A_Rs. To our knowledge, these are the first sulfated PALs reported for use in photolabeling experiments to identify binding sites for DHEAS and/or PS on either of these receptors. Some of these PALs may also find use in identifying sulfated steroid binding sites on other type of receptors. For example, transient receptor potential melastatin 3 (TRPM3) receptors are activated by PS.^20^

The finding of PAM activity on GABA_A_Rs for PALs containing the TPD group on the steroid D-ring is particularly noteworthy as only one sulfated steroid has previously be shown to have this activity.^19^ No previous structural studies have characterized a binding site for sulfated steroids with PAM activity on GABA_A_Rs. Sulfated steroids with modified D-rings have been prepared and shown to be NAMs at GluN1/GluN2A-D types of NMDARs and when a subset of the reported compounds was evaluated on GABA_A_Rs, the compounds were found to be GABA_A_R NAMs.^16,21^

Herein we showed that selected PALs photolabeled both receptors. These neurosteroid PALs will now be used to identify PAM and NAM binding sites for sulfated steroids on exogenously expressed GABA_A_ and NMDA receptors using photolabeling coupled with mass spectrometric sequencing followed by validation of functional significance using site-directed mutagenesis of residues in the putative binding sites coupled with electrophysiologic analysis of ion channel function. We anticipate that the full panel of sulfated steroid PALs will be useful, particularly since the finding of both PAM and NAM activity suggests the possibility of more than one binding site for sulfated steroids on the both GABA_A_ and NMDA receptors and different PALs may discriminate between multiple binding sites as was found for non-sulfated GABA_A_R PALs.^10–12^

## Experimental

### Chemistry

#### General methods

Extraction solvents were dried over anhydrous Na_2_SO_4_ and removed under aspirator vacuum on a rotary evaporator. ^1^H NMR and ^13^C NMR were recorded at 400 MHz and 100 Mhz, respectively, in CDCl_3_ unless indicated otherwise.

##### (3β,17β)-17-[[4-[3-(Trifluoromethyl)-3*H*-diazirin-3-yl]phenyl]methoxy]-androst-5-en-3-ol, 3-sulfate ammonium salt (KK238)

To stirred (3β,17β)-17-[[4-[3-(trifluoromethyl)-3*H*-diazirin-3-yl]phenyl]methoxy]-androst-5-en-3-ol^14^ (KK226, 28 mg, 0.057 mmol) in pyridine (4 mL) was added trimethylamine sulfur trioxide complex (139 mg, 1 mmol) and the reaction was stirred at 55 °C for 15 hours. The reaction was cooled, pyridine was removed under reduced pressure and the residue was acidified with 6 N HCl in MeOH (4 mL) to pH-3. The acidic solution was extracted with CH_2_Cl_2_ (5 × 30 mL). Solvent was removed, and to the residue was added 7 N NH_3_ in MeOH (6 mL) at room temperature. After 1 h, MeOH was removed under reduced pressure. The resulting crude ammonium salt was purified by flash column chromatography (silica gel eluted with 5–20% MeOH in CH_2_Cl_2_) to yield KK238 (34 mg, 99%): ^1^H NMR (CD_3_OD) *δ* 7.43 (d, 2H, *J* = 8.2 Hz), 7.21 (d, 2H, *J* = 8.2 Hz), 5.38–5.37 (m, 1H), 4.56 (s, 2H), 4.16–4.11 (m, 1H), 3.46 (t, 1H, *J* = 8.1 Hz), 2.60–0.92 (m, 23H), 1.03 (s, 3H), 0.83 (s, 3H); ^13^C NMR (CD_3_OD) *δ* 143.2, 141.7, 129.1 (2 × C), 128.9, 127.6 (2 × C), 123.3, 90.3, 80.0, 72.0, 52.8, 51.8, 44.2, 40.5, 39.0, 38.6, 37.8, 33.2, 32.7, 30.1, 29.0, 24.6, 22.0, 19.9, 12.3.

##### (3β,17β)-17-(2-(3-(But-3-yn-1-yl)-3*H*-diazirin-3-yl)ethoxy)-androst-5-en-3-ol, 3-sulfate ammonium salt (MQ231)

Using the sulfation and salt formation procedure described for the preparation of KK238, (3β,17β)-17-(2-(3-(but-3-yn-1-yl)-3*H*-diazirin-3-yl)ethoxy)-androst-5-en-3-ol^15^ (MQ182, 52 mg, 0.126 mmol) was converted to MQ231 (41 mg, 64%). Purification was by flash column chromatography (silica gel eluted with 10% MeOH in CH_2_Cl_2_): ^1^H NMR (CD_3_OD/CDCl_3_) *δ* 5.43–5.16 (m, 1H), 4.23–4.14 (m, 1H), 3.70–3.31 (m, 3H), 2.42–0.93 (m, 28H), 1.08 (s, 3H), 0.85 (s, 3H), 0.50–0.39 (m, 2H); ^13^C NMR (CD_3_OD/CDCl_3_) *δ* 141.7, 123.2, 90.8, 83.8, 79.9, 70.2, 65.7, 52.8, 51.8, 44.0, 40.5, 39.0, 38.5, 37.8, 34.6, 34.2, 33.1, 32.7, 30.0, 28.9, 28.2, 24.5, 21.9, 19.9, 14.0, 12.2.

##### (3β,17β)-(20-(Cyclohexylimino))-pregn-5-en-3ol (2)

To a solution of pregnenolone (1, 500 mg, 1.58 mmol) in cyclohexylamine (9.2 mL) was added CF_3_CO_2_H (8 μL) at 23 °C. The mixture was refluxed 16 h under N_2_. The solvent was removed to give steroid 2 as a light-brown semi-solid (620 mg) which was converted to steroid 3 without purification or characterization.

##### (3β,17β)-17-(3-Methyl-3*H*-diazirin3-yl)-androst-5-en-3-ol (3)

To a solution of crude compound 2 (620 mg) in MeOH (30 mL) was added NH_3_ in MeOH solution (7 N, 20 mL). NH_2_OSO_3_H (1.0 g) in MeOH (3 mL) was slowly added and the reaction was stirred for 72 h. The solvent was removed under reduced pressure and water was added to the residue. The steroid was extracted into EtOAc (50 mL). The organic layer was washed with water (20 mL), brine (20 mL), dried and filtered. The solvent was removed to yield the intermediate diaziridine product as a light yellow solid residue (520 mg) which was converted to steroid 3 without purification or characterization.

To a solution of the crude diaziridine intermediate (520 mg) in dry MeOH (20 mL) was added Et_3_N (3 mL) at 23 °C. The mixture was cooled 0 °C. I_2_ was dissolved in MeOH and added dropwise until a brown color persisted and the reaction was stirred at 23 °C for 20 min. Aqueous Na_2_S_2_O_3_ was added and the steroid product was extracted into EtOAc (2 × 50 mL). The combined organic layers were dried, filtered, the solvent was removed and the residue was purified by flash column chromatography (silica gel eluted with 10% EtOAc in hexanes) to give steroid 3 (100 mg, 19%, 3 steps): ^1^H NMR *δ* 5.32 (s, 1H), 3.52–3.48 (m, 1H), 2.26–0.62 (m, 21H), 1.00 (s, 3H), 0.98 (s, 3H), 0.85 (s, 3H); ^13^C NMR *δ* 140.7, 121.3, 71.5, 55.8, 53.6, 50.0, 43.8, 42.1, 38.6, 37.2, 36.4, 31.6, 31.5, 31.4, 26.0, 23.9, 23.4, 21.2, 20.6, 19.3, 13.0.

##### (3β,17β)-17-(3-Methyl-3*H*-diazirin3-yl)-androst-5-en-3-ol, 3-sulfate ammonium salt (YX33)

Using the sulfation procedure described for the preparation of KK238, steroid 3 (65 mg, 0.2 mmol) was converted to YX33 (40 mg, 47%). Purification was by flash column chromatography (silica gel eluted with 10% MeOH in CH_2_Cl_2_) followed by HPLC purification on a C18 reverse phase column: ^1^H NMR (CD_3_OD) *δ* 5.36–5.35 (m, 1H), 4.11–4.09 (m, 1H), 2.53–2.49 (m, 1H), 2.35–2.32 (m, 1H), 2.06–0.91 (m, 22H), 1.02 (s, 3H), 0.96 (s, 3H), 0.86 (s, 3H); ^13^C NMR (CD_3_OD) *δ* 140.2, 121.7, 78.3, 55.7, 53.6, 50.1, 43.6, 38.9, 38.5, 37.0, 36.3, 31.4, 31.4, 28.6, 25.3, 23.6, 23.0, 20.4, 20.1, 18.3, 12.2.

##### (1,1-Dimethylethyl)[[(3β,17β)-3-(methoxymethoxy)-androst-5-en-17-yl]oxy]dimethylsilane (5)

To a solution of (3β,17β)-3-(methoxymethoxy)-androst-5-en-17-ol^15^ (4, 7.33 g, 21.9 mmol) in DMF (50 mL) was added imidazole (2.8 g, 40 mmol) and TBSCl (4.0 g, 26.3 mmol) at 23 °C. After 16 h, water (50 mL) was added the product was extracted into EtOAc (2 × 150 mL). The combined organic layers were dried, filtered and the solvents removed. The residue was purified by flash column chromatography (silica gel eluted with 10% EtOAc in hexanes) to give steroid 5 (9.44 g, 96%): ^1^H NMR *δ* 5.31–5.30 (m, 1H), 4.63 (s, 2H), 3.54 (t, *J* = 8.2, 1H), 3.40–3.30 (m, 1H), 3.32 (s, 3H), 2.30–0.88 (m, 19H), 0.98 (s, 3H), 0.85 (s, 9H), 0.69 (s, 3H), −0.02 (s, 6H); ^13^C NMR *δ* 140.7, 121.3, 94.5, 81.7, 76.7, 54.9, 50.9, 50.4, 43.0, 39.5, 37.2, 36.9, 36.7, 31.9, 31.5, 30.8, 28.8, 25.8 (3 × C), 23.5, 20.6, 19.3, 18.0, 11.1, −4.6, −4.9.

##### (3β,5α,6α,17β)-17-((*tert*-Butyldimethylsilyl)oxy)-3-(methoxymethoxy)-androstan-6-ol (6) and (3β,5β,6β,17β)-17-((*tert*-butyldimethylsilyl)oxy)-3-(methoxymethoxy)-androstan-6-ol (7)

To a solution of steroid 5 (9.44 g, 21.1 mmol) in THF (150 mL) was slowly added BH_3_-THF (1.0 M in THF, 40 mL, 40 mmol) at 23 °C. After 2 h, 3 N NaOH (40 mL) and H_2_O_2_ (20 mL) were added. The reaction was stirred for 1 h at 23 °C and the mixture of steroids 6 and 7 was extracted into EtOAc (2 × 150 mL). The combined extracts were washed with brine (2 × 100 mL), dried, filtered and the solvents removed. The residue was purified by flash column chromatography (silica gel eluted with 25% EtOAc in hexanes) to give the major product, steroid 6 (6.89 g, 73%) and the minor product steroid 7 (1.2 g, 13%).

Steroid 6: ^1^H NMR *δ* 4.70 (q, 2H, *J* = 6.7 Hz), 3.56–3.39 (m, 3H), 3.36 (s, 3H), 2.25–0.60 (m, 21H), 0.87 (s, 9H), 0.81 (s, 3H), 0.68 (s, 3H), 0.00 (s, 6H); ^13^C NMR *δ* 94.3, 81.6, 76.0, 69.3, 55.1, 54.1, 51.7, 50.4, 43.3, 41.2, 37.3, 36.9, 36.4, 34.3, 30.8, 29.2, 28.4, 25.8 (3 × C), 23.5, 20.7, 18.0, 13.4, 11.3, −4.6, −4.9.

Steroid 7: ^1^H NMR *δ* 4.65 (s, 2H), 3.86 (s, 1H), 3.69 (s, 1H), 3.58 (t, 3H, *J* = 8.6 Hz), 1.88–0.91 (m, 22H), 1.14 (s, 3H), 0.87 (s, 9H), 0.72 (s, 3H), 0.00 (d, 6H, *J* = 1.2 Hz); ^13^C NMR *δ* 94.4, 81.8, 73.2, 71.1, 55.2, 50.7, 44.4, 43.4, 40.6, 37.3, 34.7, 34.0, 31.5, 30.9, 30.8, 30.7, 26.1, 25.8 (3 × C), 24.8, 23.5, 20.5, 18.1, 11.4, −4.5, −4.9.

##### (3′β,5′α,17′β)-17-((*tert*-Butyldimethylsilyl)oxy)-3-(methoxymethoxy)-androstan-6-one (8)

To a solution of steroid 6 (4.4 g, 9.44 mmol) in CH_2_Cl_2_ (60 mL) was added Dess–Martin periodinane (6.36 g, 15 mmol) and NaHCO_3_ (8.0 g) at 23 °C. After 1 h, water (50 mL) was added and the product was extracted into CH_2_Cl_2_ (150 mL × 3) and washed with brine (50 mL × 2). The organic layer was dried, filtered and the solvents removed. The residue was purified by flash column chromatography (silica gel eluted with 20% EtOAc in hexanes) to give steroid 8 (3.86 g, 88%): ^1^H NMR *δ* 4.71 (q, *J* = 6.7, 2H), 3.59 (t, 1H, *J* = 8.2 Hz), 3.52–3.36 (m, 1H), 3.45 (s, 3H), 2.32–1.02 (m, 20H), 0.86 (s, 9H), 0.75 (s, 3H), 0.69 (s, 3H), −0.00 (s, 6H); ^13^C NMR *δ* 210.4, 94.3, 81.4, 75.1, 56.8, 55.1, 54.2, 51.0, 46.2, 43.7, 41.0, 37.9, 36.7, 36.6, 30.7, 28.1, 26.9, 25.8 (3 × C), 23.3, 21.1, 18.0, 13.1, 11.3, −4.5, −4.9.

##### (3′β,5′β,17′β)-17-((*tert*-Butyldimethylsilyl)oxy)-3-(methoxymethoxy)-androstan-6-one (9)

Using the procedure described for the preparation of 5α-steroid 8, the 5β-steroid 7 (830 mg, 1.78 mmol) was converted to steroid 9. Purification was by flash column chromatography (silica gel eluted with 10% EtOAc in hexanes). Steroid 9 (811 mg, 98%): ^1^H NMR *δ* 4.61 (s, 2H), 3.88 (s, 1H), 3.60 (t, 1H *J* = 7.8 Hz), 3.34 (s, 3H), 2.43–1.02 (m, 20H), 0.86 (s, 12H), 0.68 (s, 3H), −0.01 (s, 6H); ^13^C NMR *δ* 214.8, 94.4, 81.4, 69.4, 55.2, 55.0, 51.2, 43.8, 42.5, 39.8, 37.9, 37.0, 36.9, 30.7, 30.4, 29.3, 25.8 (3 × C), 25.2, 23.8, 23.2, 20.8, 18.0, 11.3, −4.6, −4.9.

##### (3′β,5′α,17′β)-17-Hydroxy-3-(methoxymethoxy)-androstan-6-one (10)

To a solution of steroid 8 (3.86 g, 8.32 mmol) in THF (50 mL) was added TBAF (1.0 M in THF, 15 mL, 15 mmol) at 23 °C. The reaction was refluxed for 16 h. THF was removed and the residue was purified by flash column chromatography (silica gel eluted with 30% EtOAc in hexanes) to give steroid 10 (2.80 g, 96%): ^1^H NMR *δ* 4.70 (q, 2H, *J* = 6.6 Hz), 3.69 (t, 1H, *J* = 7.4 Hz), 3.51–3.43 (m, 1H), 3.36 (s, 3H), 2.34–1.11 (m, 21H), 0.77 (s, 3H), 0.74 (s, 3H); ^13^C NMR *δ* 210.4, 94.4, 81.5, 75.1, 56.8, 55.2, 54.0, 51.3, 46.2, 43.4, 41.0, 37.9, 36.7, 36.3, 30.3, 28.1, 26.9, 23.1, 21.1, 13.1, 11.1.

##### (3′β,5′β,17′β)-17′-((*tert*-Butyldimethylsilyl)oxy)-3′-(methoxymethoxy)-spiro[3*H*-diazirine-3,6′-androstane] (11)

NH_3_ (6 mL, 7.0 N in MeOH, 42 mmol) was added to a solution of steroid 9 (710 mg, 1.53 mmol) in MeOH (20 mL) at 23 °C. After 24 h, hydroxylamine-*O*-sulfonic acid (234 mg, 1.68 mmol) in MeOH (5 mL) was added and the reaction was stirred for 72 h. The reaction was filtered and the precipitate was washed with EtOAc (100 mL). Solvents were removed and the residue was dissolved in MeOH (10 mL). Et_3_N (1.0 mL) was added followed by I_2_ in MeOH until a brown color persisted. Aqueous Na_2_S_3_O_3_ (50 mL) was added and the product was extracted into CH_2_Cl_2_ (2 × 100 mL) and the combined extracts were washed with brine (2 × 50 mL), dried, filtered and the solvents removed. The residue was purified by flash column chromatography (silica gel eluted with 5% EtOAc in hexanes) to give steroid 11 (30 mg, 4%) and steroid 12 (310 mg, 43%). Steroid 11 had: ^1^H NMR *δ* 4.58 (s, 2H), 3.91 (s, 1H), 3.58 (t, 1H, *J* = 8.7 Hz), 3.31 (s, 3H), 1.88–0.94 (m, 18H), 1.20 (s, 3H), 0.88 (s, 9H), 0.73 (s, 3H), 0.52–0.47 (m, 1H), 0.28–0.24 (m, 1H), 0.01 (d, 6H, *J* = 4.3 Hz); ^13^C NMR *δ* 94.5, 81.6, 70.3, 55.2, 50.6, 44.1, 43.5, 39.6, 37.1, 36.7, 34.0, 32.7, 30.8, 29.7, 29.3, 26.6, 25.8 (2 × C), 24.8, 23.6, 23.2, 20.6, 18.1, 11.3, −4.5, −4.8.

##### (3′β,5′α,17′β)-3′-(Methoxymethoxy)-spiro[3*H*-diazirine-3,6′-androstan]-17′-ol (12)

Using the procedure described for the preparation of diazirine 11, steroid 10 (2.8 g, 8 mmol) was converted to steroid 12 (2.5 g, 86%). Purification was by flash column chromatography (silica gel eluted with 25% EtOAc in hexanes): ^1^H NMR *δ* 4.53 (q, 2H, *J* = 7.4 Hz), 3.57 (t, 1H, *J* = 8.6 Hz), 3.33–3.27 (m, 1H), 3.23 (s, 3H), 2.05–0.72 (m, 19H), 1.08 (s, 3H), 0.70 (s, 3H), 0.43–0.33 (m, 2H); ^13^C NMR *δ* 94.2, 81.3, 75.5, 54.9, 53.7, 50.5, 45.1, 42.9, 37.6, 36.8, 36.3, 36.2, 33.8, 30.2, 30.1, 29.1, 27.8, 22.9, 20.6, 12.8, 11.0.

##### (3′β,5′β,17′β)-3′-(Methoxymethoxy)-spiro[3*H*-diazirine-3,6′-androstan]-17′-ol (13)

Using the procedure described for the preparation of compound 10, steroid 11 (30 mg, 0.063 mmol) was converted into steroid 13 (23 mg, 74%). Purification was by flash column chromatography (silica gel eluted with 25% EtOAc in hexanes): ^1^H NMR *δ* 4.57 (s, 2H), 3.90 (s, 1H), 3.66 (t, 1H, *J* = 8.2 Hz), 3.29 (s, 3H), 2.06–1.00 (m, 19H), 1.20 (s, 3H), 0.76 (s, 3H), 0.52–0.47 (m, 1H), 0.28–0.23 (m, 1H); ^13^C NMR *δ* 94.5, 81.7, 70.3, 55.2, 50.9, 44.0, 43.2, 39.5, 36.7, 36.6, 34.0, 32.7, 30.4, 29.6, 29.2, 26.6, 24.8, 23.6, 23.0, 20.5, 11.1.

##### (3′β,5′α,17′β)-17′-(Prop-2-yn-1yloxy)-spiro[3*H*-diazirine-3,6′-androstan]-3′-ol (14)

To a solution of steroid 12 (2.5 g, 6.9 mmol) in THF (60 mL) was added NaH (2.24 g, 56 mmol) at 23 °C. The mixture was refluxed for 1 h and then propargyl bromide (10 mL, 80% in toluene) was added. The mixture was refluxed 16 h. After cooling to 23 °C, water was added and the product was extracted into EtOAc (3 × 100 mL). The combined extracts were dried, filtered and the solvents removed. The residue was dissolved in MeOH (50 mL) and acetyl chloride (4 mL) was added at 23 °C. After 1 h, water was added and the product was extracted into CH_2_Cl_2_ (3 × 100 mL). The combined extracts were washed with aqueous NaHCO_3_ (2 × 50 mL), dried, filtered and the solvents removed. The residue was purified by flash column chromatography (silica gel eluted with 25% EtOAc in hexanes) to give steroid 14 (1.52 g, 62%, 2 steps): ^1^H NMR *δ* 4.18–4.08 (m, 2H), 3.53 (t, 1H, *J* = 8.2 Hz), 3.47–3.40 (m, 1H), 2.38 (t, 1H, *J* = 2.4 Hz), 2.02–0.71 (m, 19H), 1.12 (s, 3H), 0.78 (s, 3H), 0.45–0.36 (m, 2H); ^13^C NMR *δ* 87.8, 80.4, 73.8, 70.8, 57.1, 53.7, 50.7, 45.2, 42.9, 37.5, 37.3, 36.8, 36.3, 33.6, 33.0, 30.6, 29.1, 27.4, 23.0, 20.8, 12.9, 11.6.

##### (3′β,5′α,17′β)-17′-(Prop-2-yn-1-yloxy)-spiro[3*H*-diazirine-3,6′-androstan]-3′-ol, 3′-sulfate ammonium salt (MQ189)

Using the sulfation procedure described for the preparation of KK238, steroid 14 (250 mg, 0.70 mmol) was converted to MQ189 (283 mg, 89%). Purification was by flash column chromatography (silica gel eluted with 10% MeOH in CH_2_Cl_2_): ^1^H NMR (CD_3_OD) *δ* 4.22–4.11 (m, 3H), 3.63 (t, 1H, *J* = 7.9, Hz), 3.38 (s, br, 1H), 2.82 (s, 1H), 2.09–0.92 (m, 21H), 1.20 (s, 3H), 0.84 (s, 3H), 0.50–0.39 (m, 2H); ^13^C NMR (CD_3_OD) *δ* 89.2, 81.4, 79.4, 75.4, 58.1, 55.0, 51.9, 46.4, 44.3, 38.7, 38.6, 38.0, 37.5, 35.1, 31.6, 30.2, 29.2, 28.5, 24.2, 22.0, 13.4, 12.3.

##### (3′β,5′β,17′β)-3′-(Methoxymethoxy)-17′-(prop-2-yn-1-yloxy)-spiro[3*H*-diazirine-3,6′-androstane] (15)

Using the procedure described for the preparation of compound 3, steroid 13 (23 mg, 0.063 mmol) was converted to steroid 15 (21 mg, 83%). Purification was by flash column chromatography (silica gel eluted with 10% EtOAc in hexanes): ^1^H NMR *δ* 4.57 (s, 2H), 4.20–4.11 (m, 2H), 3.91 (s, 1H), 3.57 (t, 1H, *J* = 8.3 Hz), 3.30 (s, 3H), 2.39 (s, 1H), 2.03–1.02 (m, 18H), 1.20 (s, 3H), 0.79 (s, 3H), 0.52–0.48 (m, 1H), 0.28–0.23 (m, 1H); ^13^C NMR *δ* 94.5, 87.9, 80.5, 73.8, 70.3, 57.2, 55.2, 51.1, 44.0, 43.1, 39.5, 37.6, 36.7, 33.7, 32.7, 29.6, 29.2, 27.5, 26.6, 24.8, 23.6, 23.1, 20.5, 11.7.

##### (3′β,5′β,17′β)-17′-(prop-2-yn-1-yloxy)-spiro[3*H*-diazirine-3,6′-androstan]-3′-ol (16)

To steroid 15 (21 mg, 0.053 mmol) in THF (5 mL) was added 6 N HCl (5 mL) at 23 °C. After 2 h, the product was extracted into CH_2_Cl_2_ (2 × 100 mL). Solvent was removed and the residue was purified by flash column chromatography (silica gel eluted with 25% EtOAc in hexanes) to give steroid 16 (16 mg, 74%): ^1^H NMR *δ* 4.20–4.10 (m, 3H), 3.57 (t, 1H, *J* = 8.2 Hz), 2.40 (d, 1H *J* = 1.5 Hz), 2.03–1.01 (m, 19H), 1.21 (s, 3H), 0.79 (s, 3H), 0.56–0.52 (m, 1H), 0.27–0.24 (m, 1H); ^13^C NMR *δ* 87.9, 80.5, 73.8, 73.7, 65.3, 57.2, 51.1, 43.3, 43.1, 39.3, 37.5, 36.9, 33.7, 32.7, 29.2, 29.0, 28.8, 27.5, 23.6, 23.0, 20.5, 11.7.

##### (3′β,5′β,17′β)-17′-(prop-2-yn-1-yloxy)-spiro[3*H*-diazirine-3,6′-androstan]-3′-ol, 3′-sulfate ammonium salt (MQ234)

Using the sulfation procedure described for the preparation of KK238, steroid 16 (16 mg, 0.045 mmol) was converted to MQ234 (19 mg, 93%). Purification was by flash column chromatography (silica gel eluted with 10–25% MeOH in CH_2_Cl_2_): ^1^H NMR (CD_3_OD) *δ* 4.71 (s, 1H), 4.21–4.12 (m, 2H), 3.64 (t, 1H, *J* = 8.3 Hz), 2.81 (s, 1H), 2.07–1.12 (m, 22H), 1.22 (s, 3H), 0.81 (s, 3H), 0.54–0.49 (m, 1H), 0.25–9.21 (m, 1H); ^13^C NMR (CD_3_OD) *δ* 89.3, 81.5, 75.4, 75.2, 75.2, 58.1, 52.1, 45.5, 44.4, 40.8, 38.7, 37.8, 35.2, 33.7, 30.7, 30.1, 28.6, 28.0, 26.2, 24.2, 21.8, 12.2.

##### 3-(4-(((3β,5β,17β)-3-(Methyoxymethoxy)-androstan-17-yl)oxymethyl)phenyl)-3-(trifluoromethyl)-3*H*-diazirine (18)

To a solution of (3β,5β,17β)-3-(methoxymethoxy)-androstan-17-ol^16^ (17, 110 mg, 0.327 mmol) in THF (20 mL) was added 3-[4-(iodomethyl)phenyl]-3-(trifluoromethyl)-3*H*-diazirine (326 mg, 1 mmol) in THF (10 mL), Bu_4_NI (80 mg) and NaH (160 mg, 60% in mineral oil, 4 mmol) at 23 °C. The reaction was gently refluxed for 16 h. After cooling to 23 °C, water was added and the product was extracted into EtOAc (2 × 150 mL). Solvent was removed and the residue was purified by flash column chromatography (silica gel eluted with 10% EtOAc in hexanes) to give steroid 18 (104 mg, 59%): ^1^H NMR *δ* 7.41 (d, 2H, *J* = 8.2 Hz), 7.36 (d, 2H, *J* = 8.2 Hz), 4.67 (s, 2H), 4.55 (s, 2H), 3.90 (s, 1H), 3.38 (s, 3H), 2.02–0.84 (m, 23H), 0.98 (s, 3H), 0.82 (s, 3H); ^13^C NMR *δ* 141.3, 127.9, 127.5 (2 × C), 126.4 (2 × C), 94.5, 88.7, 72.1, 71.5, 70.7, 51.3, 43.3, 40.2, 38.2, 37.1, 35.5, 35.0, 31.2, 30.6, 27.9, 26.6, 25.8, 25.3, 23.9, 23.4, 20.7, 11.8.

##### (3β,5β,17β)-17-[[4-[3-(Trifluoromethyl)-3*H*-diazirin-3-yl]phenyl]methoxy]-androstan-3-ol (19)

Using the procedure described for the preparation of steroid 16, diazirine 18 (104 mg, 0.195 mmol) was converted to diazirine 19 (54 mg, 57%). Purification was by flash column chromatography (silica gel eluted with 25% EtOAc in hexanes): ^1^H NMR *δ* 7.39 (d, 2H, *J* = 8.2 Hz), 7.28 (d, 2H, *J* = 8.2 Hz), 4.55 (s, 2H), 4.12 (s, 1H), 3.41 (t, 1H, *J* = 8.2 Hz), 2.06–0.90 (m, 23H), 0.99 (s, 3H), 0.83 (s, 3H); ^13^C NMR *δ* 141.3, 127.9, 127.5 (2 × C), 126.4 (2 × C), 86.7, 70.7, 67.1, 51.3, 43.3, 40.0, 38.2, 36.6, 35.4, 35.2, 33.5, 30.0, 27.9, 27.8, 26.5, 25.8, 23.9, 23.4, 20.7, 11.8.

##### (3β,5β,17β)-17-[[4-[3-(Trifluoromethyl)-3*H*-diazirin-3-yl]phenyl]methoxy]-androstan-3-ol, 3-sulfate ammonium salt (MQ235)

Using the sulfation procedure described for the preparation of KK238, steroid 19 (23 mg, 0.047 mmol) was converted to MQ235 (32 mg, 74%). Purification was by flash column chromatography (silica gel eluted with 10–25% MeOH in CH_2_Cl_2_): ^1^H NMR (CD_3_OD) *δ* 7.45 (d, 2H, *J* = 7.8 Hz), 7.24 (d, 2H, *J* = 7.8 Hz), 4.69 (s, 1H), 4.57 (s, 2H), 3.49 (t, 1H, *J* = 8.2 Hz), 2.07–0.97 (m, 26H), 1.00 (s, 3H), 0.84 (s, 3H); ^13^C NMR (CD_3_OD) *δ* 143.3, 129.0 (2 × C), 128.9, 127.6 (2 × C), 90.4, 77.22, 77.20, 72.0, 52.6, 44.6, 41.6, 39.4, 38.5, 37.0, 36.0, 32.6, 31.7, 29.1, 27.7, 27.0, 26.9, 24.6, 24.5, 22.0, 12.4.

##### 3-(4-(((3α,5β,17β)-3-(Methyoxymethoxy)-androstan-17-yl)oxymethyl)phenyl)-3-(trifluoromethyl)-3*H*-diazirine (21)

Using the procedure described for the preparation of steroid 18, (3α,5β,17β)-3-(methoxymethoxy)-androstan-17-ol (20, 150 mg, 0.45 mmol) was converted to diazirine 21 (151 mg, 63%). Purification was by flash column chromatography (silica gel eluted with 10% EtOAc in hexanes): ^1^H NMR *δ* 7.37 (d, 2H, *J* = 8.6 Hz), 7.17 (d, 2H, *J* = 8.6 Hz), 4.68 (s, 2H), 4.53 (s, 2H), 3.54–3.38 (m, 1H), 3.36 (s, 3H), 1.99–0.82 (m, 23H), 0.93 (s, 3H), 0.80 (s, 3H); ^13^C NMR *δ* 141.3, 127.9, 127.4 (2 × C), 126.4 (2 × C), 94.5, 88.8, 70.8, 55.1, 51.1, 51.2, 43.2, 42.1, 40.6, 38.1, 35.6, 34.8, 33.5, 27.9, 27.7, 27.0, 25.9, 25.3, 23.4, 23.3, 20.4, 11.8.

##### (3α,5β,17β)-17-[[4-[3-(Trifluoromethyl)-3*H*-diazirin-3-yl]phenyl]methoxy]-androstan-3-ol (22)

Using the procedure described for the preparation of steroid 16, diazirine 21 (151 mg, 0.28 mmol) was converted to diazirine 22 (125 mg, 91%). Purification was by flash column chromatography (silica gel eluted with 25% EtOAc in hexanes): ^1^H NMR *δ* 7.38 (d, 2H, *J* = 8.6 Hz), 7.18 (d, 2H, *J* = 8.6, Hz), 4.54 (s, 2H), 3.66–3.58 (m, 1H), 3.41 (t, 1H, *J* = 8.2 Hz), 2.04–0.83 (m, 23H), 0.93 (s, 3H), 0.81 (s, 3H); ^13^C NMR *δ* 141.2, 127.9, 127.4 (2 × C), 126.3 (2 × C), 88.7, 71.7, 70.7, 51.2, 43.2, 42.0, 40.6, 38.1, 36.3, 35.6, 35.3, 34.6, 30.4, 27.9, 27.0, 25.9, 23.4, 23.3, 20.4, 11.8.

##### (3α,5β,17β)-17-[[4-[3-(Trifluoromethyl)-3*H*-diazirin-3-yl]phenyl]methoxy]-androstan-3-ol, 3-sulfate ammonium salt (MQ236)

Using the sulfation procedure described for the preparation of KK238, diazirine 22 (23 mg, 0.047 mmol) was converted to MQ236 (26 mg, 93%). Purification was by flash column chromatography (silica gel eluted with 10–25% MeOH in CH_2_Cl_2_): ^1^H NMR (CD_3_OD) *δ* 7.45 (d, 2H, *J* = 8.2 Hz), 7.23 (d, 2H, *J* = 8.2 Hz), 4.87 (s, 2H), 4.32–4.26 (m, 1H), 3.50 (t, 1H, *J* = 8.2 Hz), 2.07–0.97 (m, 26H), 0.97 (s, 3H), 0.82 (s, 3H); ^13^C NMR (CD_3_OD) *δ* 143.3, 129.1 (2 × C), 128.9, 127.6 (2 × C), 90.4, 80.5, 72.1, 52.5, 44.5, 43.8, 42.1, 39.4, 37.2, 36.6, 35.8, 34.7, 29.1, 29.0, 28.3, 27.2, 24.6, 23.9, 21.7, 19.5, 12.4.

##### (3β,5α)-3-Hydroxyandrostan-17-one, 3-mesylate (24)

To a solution of (3β,5α)-3-hydroxyandrostan-17-one (23, 1.5 g, 5.2 mmol) in CH_2_Cl_2_ (30 mL) was added mesyl chloride (0.62 mL, 8 mmol) and Et_3_N (1.54 mL, 11 mmol) at 0 °C. After 2 h, 5% HCl (20 mL) was added and the product was extracted into CH_2_Cl_2_ (2 × 100 mL). The solvent was removed and the residue was purified by flash column chromatography (silica gel eluted with 25% EtOAc in hexanes) to give mesylate 24 (1.88 g, 98%): ^1^H NMR *δ* 4.62–4.54 (m, 1H), 2.97 (s, 3H), 2.44–2.42 (m, 1H), 2.08–0.65 (m, 21H), 0.83 (s, 6H); ^13^C NMR *δ* 220.9, 81.7, 54.0, 51.1, 47.6, 44.6, 38.7, 36.6, 35.7, 35.3, 34.9, 34.8, 31.3, 30.6, 28.5, 28.0, 21.6, 20.3, 13.7, 12.0.

##### (5α)-Androst-2-en-17-one and (5α)-androst-3-en-17-one mixture (25)

To mesylate 24 (1.88 g, 5.16 mmol) in dry DMF (40 mL) was added LiBr (1.8 g, 20.7 mmol) at 23 °C. The reaction was heated to 130 °C for 90 min. After cooling, water (30 mL) was added and the product was extracted into EtOAc (2 × 150 mL). The solvent was removed and the residue was purified by flash column chromatography (silica gel eluted with 10% EtOAc in hexanes) to give the inseparable Δ^2^ and Δ^3^ double bond products 25 in a 5 : 1 ratio (1.26 g, 91%). The major Δ^2^ product had: ^1^H NMR *δ* 5.61–5.52 (m, 2H), 2.43–0.95 (m, 20H), 0.84 (s, 3H), 0.77 (s, 3H); ^13^C NMR *δ* 221.2, 125.6, 125.6, 54.0, 51.3, 47.5, 41.3, 39.6, 35.7, 35.0, 34.6, 31.5, 30.5, 30.1, 28.3, 21.6, 20.1, 13.6, 11.5.

##### (5α,17β)-Androst-2-en-17-ol and (5α,17β)-androst-3-en-17-ol mixture (26)

To a solution of the inseparable steroids 25 (1.26 g, 4.7 mmol) in EtOH (50 mL) was added NaBH_4_ (378 mg, 10 mmol) at 23 °C. After 1 h, aqueous NH_4_Cl (30 mL) was added and the reaction was stirred for 10 min. EtOH was removed under reduced pressure and the product was extracted into EtOAc (2 × 150 mL). The solvent was removed and the residue was purified by flash column chromatography (silica gel eluted with 25% EtOAc in hexanes) to give unseparated Δ^2^ and Δ^3^ double bond products 26 in a 5 : 1 ratio product (1.20 g, 95%): The major Δ^2^ product had: ^1^H NMR *δ* 5.63–5.56 (m, 2H), 3.66 (t, 1H, *J* = 8.6 Hz), 2.04–0.68 (m, 21H), 0.78 (s, 3H), 0.75 (s, 3H); ^13^C NMR *δ* 125.8, 125.8, 82.0, 54.2, 51.0, 42.9, 41.5, 36.7, 35.6, 31.4, 30.5, 30.3, 28.6, 23.4, 21.0, 20.5, 14.2, 11.7, 11.0.

##### (2α,3α,5α,17β)-2,3-Epoxyandrostan-17-ol (27)

To a solution of the unseparated steroids 26 (1.20 g, 4.44 mmol) in CH_2_Cl_2_ (35 mL) was added aqueous formic acid (10 mL) and aqueous 30% H_2_O_2_ (10 mL) at 23 °C. After 2 h, the product was extracted into CH_2_Cl_2_ (2 × 100 mL) washed with brine (2 × 50 mL), dried, the solvent was removed and the residue was purified by flash column chromatography (silica gel eluted with 25% EtOAc in hexanes) to give the 2,3-epoxide 27 which also contained the unseparated 3,4-epoxide (1.04 g, 81%). 2,3-Epoxide 27 had: ^1^H NMR *δ* 3.59 (t, 1H, *J* = 8.6 Hz), 3.11–3.06 (m, 2H), 1.98–0.54 (m, 21H), 0.72 (s, 3H), 0.67 (s, 3H); ^13^C NMR *δ* 81.6, 53.7, 52.4, 51.0, 50.7, 38.1, 36.5, 36.2, 35.5, 33.6, 33.1, 31.1, 30.1, 28.9, 28.1, 23.2, 20.3, 12.8, 10.9.

##### (1,1-Dimethylethyl)[[(2α,3α,5α,17β)-2,3-epoxyandrostan-17-yl]oxy]dimethylsilane (28)

Using the procedure described for the preparation of steroid 5, 2,3-epoxide 27 containing the 3,4-epoxide (1.04 g, 3.61 mmol) was converted to 2,3-epoxide 28 containing the 3,4-epoxide (1.24 g, 86%). Purification was by flash column chromatography (silica gel eluted with 10% EtOAc in hexanes). 2,3-Epoxide 28 had: ^1^H NMR *δ* 3.54 (t, 1H, *J* = 8.6 Hz), 3.13–3.08 (m, 2H), 1.93–0.56 (m, 20H), 0.87 (s, 9H), 0.75 (s, 3H), 0.67 (s, 3H), 0.00 (s, 3H), −0.01 (s, 3H); ^13^C NMR *δ* 81.7, 53.9, 52.3, 50.9, 50.4, 43.0, 38.3, 37.0, 36.2, 35.7, 33.6, 31.2, 30.8, 29.0, 28.3, 25.8 (3 × C), 23.4, 20.5, 18.0, 12.9, 11.2, −4.6, −3.9.

##### (2β,3α,5α,17β)-2-(But-3-yn-yloxy)-17-[[(1,1-dimethylethyl)dimethylsilyl]oxy]-androstan-3-ol (29)

To a solution of 2,3-epoxide 28 containing the 3,4-epoxide epoxide (856 mg, 2.13 mmol) in 3-butyn-1-ol (3 mL) was added tetracyanoethylene (800 mg) at 23 °C. After 72 h, 3-butyn-1-ol was removed under reduced pressure and the residue was purified by flash column chromatography (silica gel eluted with 25% EtOAc in hexanes) to give steroid 29 (440 mg, 44%): ^1^H NMR *δ* 4.66 (t, 1H, *J* = 5.8 Hz), 3.90 (s, 1H), 3.63–3.43 (m, 3H), 2.79–2.75 (m, 1H), 2.42–2.38 (m, 2H), 2.21–0.59 (m, 21H), 0.92 (s, 3H), 0.86 (s, 9H), 0.67 (s, 3H), −0.02 (s, 6H); ^13^C NMR *δ* 81.7, 79.0, 75.2, 73.1, 69.1, 68.4, 67.0, 55.2, 50.6, 43.2, 39.0, 37.1, 36.2, 35.9, 34.9, 32.1, 31.5, 30.8, 28.0, 25.8, 23.4, 20.5, 20.1, 19.8, 18.0, 13.3, 11.3, −4.6, −4.8.

##### (1,1-Dimethylethyl)[[(2β,3α,5α,17β)-2-(but-3-yn-yloxy)-3-(methoxymethoxy)-androstan-17-yl]oxy]dimethylsilane (30)

To a solution of steroid 29 (440 mg, 0.93 mmol) in CH_2_Cl_2_ (15 mL) was added ClCH_2_OMe (0.2 mL) and (i-Pr)_2_NEt (0.42 mL, 3 mmol) at 23 °C. The reaction was stirred at 23 °C for 16 h. Water was added and the product was extracted into EtOAc (2 × 150 mL). Solvent was removed and the residue was purified by flash column chromatography (silica gel eluted with 10% EtOAc in hexanes) to give steroid 30 (378 mg, 78%): ^1^H NMR *δ* 4.68–4.63 (m, 2H), 3.75 (s, 1H), 3.64–3.45 (m, 4H), 3.36 (s, 3H), 2.43–2.39 (m, 2H), 1.96–1.20 (m, 18H), 0.96–0.84 (m, 3H), 0.93 (s, 3H), 0.87 (s, 9H), 0.68 (s, 3H), 0.00 (s, 6H); ^13^C NMR *δ* 95.3, 81.8, 81.2 (2 × C), 77.4, 73.9, 69.1, 67.0, 55.3, 50.7, 43.3, 39.7, 37.2, 36.9, 35.7, 35.0, 31.6, 30.9, 29.8, 28.1, 25.8 (3 × C), 23.4, 20.5, 20.2, 18.1, 13.3, 11.4, −4.5, −4.8.

##### (2β,3α,5α,17β)-2-(But-3-yn-1-yloxy)-3-(methoxymethoxy)-androstan-17-ol (31)

Using the procedure described for the preparation of compound 10, steroid 30 (378 mg, 0.732 mmol) was converted into steroid 31 (212 mg, 71%). Purification was by flash column chromatography (silica gel eluted with 25% EtOAc in hexanes): ^1^H NMR *δ* 4.65–4.61 (m, 2H), 3.72 (s, 1H), 3.62–3.56 (m, 4H), 3.35 (s, 3H), 2.41–2.38 (m, 2H), 2.03–0.64 (m, 22H), 0.92 (s, 3H), 0.69 (s, 3H); ^13^C NMR *δ* 95.3, 81.8, 81.4, 77.3, 73.8, 69.1, 67.0, 55.3, 55.1, 51.0, 42.9, 39.6, 36.7, 36.7, 35.6, 34.9, 31.5, 30.4, 29.7, 28.0, 23.3, 20.4, 20.1, 13.2, 11.1.

##### 3-(4-(((2β,3α,5β,17β)-2-(But-3-yn-1-yloxy)-3-(methyoxymethoxy)-androstan-17-yl)oxymethyl)phenyl)-3-(trifluoromethyl)-3*H*-diazirine (32)

Using the procedure described for the preparation of compound 18, steroid 31 (210 mg, 0.52 mmol) was converted to steroid 32 (164 mg, 52%). Purification was by flash column chromatography (silica gel eluted with 10% EtOAc in hexanes): ^1^H NMR *δ* 7.37 (d, 2H, *J* = 8.8 Hz), 7.17 (d, 2H, *J* = 8.8 Hz), 4.73–4.68 (s, 2H), 4.67 (s, 2H), 4.73 (s, 1H), 3.59–3.39 (m, 4H), 3.36 (s, 3H), 2.44–2.37 (m, 2H), 2.02–0.66 (m, 21H), 0.93 (s, 3H), 0.80 (s, 3H); ^13^C NMR *δ* 141.3, 127.8, 127.4 (2 × C), 126.3 (2 × C), 95.3, 88.7, 81.4, 77.4, 73.9, 70.7, 69.1, 67.0, 55.3, 55.1, 51.2, 43.1, 39.6, 38.0, 36.8, 35.6, 34.7, 31.5, 29.8, 28.0, 27.8, 23.3, 20.5, 20.2, 13.3, 11.8.

##### (2β,3α,5β,17β)-2-(But-3-yn-1-yloxy)-17-[[4-[3-(trifluoromethyl)-3*H*-diazirin-3-yl]phenyl]methoxy]-androstan-3-ol (33)

Using the procedure described for the preparation of steroid 16, diazirine 32 (164 mg, 0.327 mmol) was converted to diazirine 33 (104 mg, 68%). Purification was by flash column chromatography (silica gel eluted with 25% EtOAc in hexanes): ^1^H NMR *δ* 7.38 (d, 2H, *J* = 8.8 Hz), 7.18 (d, 2H, *J* = 8.8 Hz), 4.54 (s, 2H), 4.96 (s, 1H), 3.68–3.38 (m, 3H), 3.36 (t, 1H, *J* = 8.6 Hz), 2.45–2.41 (m, 2H), 2.00–0.66 (m, 22H), 0.95 (s, 3H), 0.81 (s, 3H); ^13^C NMR *δ* 141.3, 127.9, 127.4 (2 × C), 126.4 (2 × C), 99.7, 81.5, 79.1, 70.7, 69.1, 68.5, 67.0, 55.2, 51.2, 43.1, 39.0, 38.0, 36.3, 35.9, 34.7, 32.1, 31.5, 28.0, 27.8, 23.3, 20.5, 20.2, 13.3, 11.9.

##### (2β,3α,5β,17β)-2-(But-3-yn-1-yloxy)-17-[[4-[3-(trifluoromethyl)-3*H*-diazirin-3-yl]phenyl]methoxy]-androstan-3-ol 3-sulfate, ammonium salt (MQ237)

Using the sulfation procedure described for the preparation of KK238, diazirine 33 (60 mg, 0.108 mmol) was converted to MQ237 (21 mg, 30%). Purification was by flash column chromatography (silica gel eluted with 10–25% MeOH in CH_2_Cl_2_): ^1^H NMR (400 MHz, CD_3_OD) *δ* 7.44 (d, 2H, *J* = 7.8 Hz), 7.22 (d, 2H, *J* = 7.8 Hz), 4.55 (s, 2H), 4.41 (s, 1H), 3.61–3.42 (m, 4H), 2.41–0.67 (m, 27H), 0.96 (s, 3H), 0.81 (s, 3H); ^13^C NMR (100 MHz, CD_3_OD) *δ* 141.7, 127.5 (2 × C), 127.3, 126.0 (2 × C), 88.9, 80.9, 76.7, 74.6, 70.4, 68.8, 67.2, 55.2, 51.0, 42.9, 39.2, 37.7, 36.9, 35.1, 34.7, 31.3, 29.4, 27.6, 27.4, 22.9, 20.2, 19.5, 12.6, 10.9.

##### (3β,5α,17β)-Spiro[androstane-3,17-diol-6,2′[1,3]dioxolane] (34)

To a solution of steroid 10 (1.65 g, 4.7 mmol) in benzene (150 mL) wad added ethylene glycol (5.0 mL) and PTSA (200 mg) at 23 °C. The reaction was refluxed in a flask equipped with a Dean–Stark trap for 16 h. After cooling, solid NaHCO_3_ (2 g) was added and stirred for 15 min. The product was extracted into EtOAc (250 mL). The organic layer was washed with brine (5 × 100 mL), solvent was removed and the residue was purified by flash column chromatography (silica gel eluted with 40% EtOAc in hexanes) to give steroid 34 (934 mg, 57%): ^1^H NMR *δ* 3.96–3.56 (m, 6H), 1.96–0.67 (m, 22H), 0.95 (s, 3H), 0.75 (s, 3H); ^13^C NMR *δ* 109.5, 81.8, 71.5, 65.5, 64.3, 53.7, 50.7, 50.6, 43.0, 41.0, 38.2, 36.9, 36.5, 33.4, 31.1, 30.4, 29.1, 23.3, 20.7, 14.2, 11.1.

##### (5α)-Spiro[androstane-3,17-dione-6,2′[1,3]dioxolane] (35)

Using the procedure described for the preparation of compound 8, steroid 34 (164 mg, 0.327 mmol) was converted to steroid 35 (913 mg, 98%). Purification was by flash column chromatography (silica gel eluted with 25% EtOAc in hexanes): ^1^H NMR *δ* 3.95–3.76 (m, 4H), 2.51–0.78 (m, 20H), 1.09 (s, 3H), 0.86 (s, 3H); ^13^C NMR *δ* 220.5, 212.0, 108.6, 65.6, 64.2, 53.0, 51.3, 50.7, 47.6, 39.8, 38.8, 37.5, 36.9, 36.2, 35.6, 32.9, 31.2, 21.6, 20.4, 13.7, 13.3.

##### (3β,5α)-Spiro[3-(methoxymethoxy)-androstan-17-one-6,2′[1,3]dioxolane] (36)

To a solution of steroid 35 (913 mg, 2.64 mmol) in THF (40 mL) was slowly added lithium tri-*tert* butoxide aluminum hydride (1.0 M in THF, 5 mL, 5 mmol) at −40 °C. After 1 h, water (30 mL) was added at −40 °C and then warmed to 23 °C. The product was extracted into EtOAc (2 × 200 mL). The combined extracts were dried, filtered and the solvents removed. The crude product was dissolved in CH_2_Cl_2_ (50 mL), (i-Pr)_2_EtN (1.3 mL, 7.5 mmol) and ClCH_2_OMe (0.45 mL, 6.0 mmol) were added and the reaction was stirred at 23 °C for 16 h. Water was added and the product extracted into CH_2_Cl_2_. The combined extracts were washed with brine, dried and solvent removed to give a viscous liquid that was purified by flash column chromatography (silica gel eluted with 25–35% EtOAc in hexanes) to give steroid 36 as a colorless liquid (859 mg, 2 steps, 84%): ^1^H NMR *δ* 4.69 (s, 2H), 3.99–3.81 (m, 4H), 3.54–3.47 (m, 1H), 3.37 (s, 3H), 2.49–0.73 (m, 20H), 0.97 (s, 3H), 0.88 (s, 3H); ^13^C NMR *δ* 221.0, 109.3, 94.4, 76.0, 65.6, 64.4, 55.2, 53.7, 50.9, 50.8, 47.8, 40.3, 38.1, 37.1, 35.8, 33.0, 31.3, 28.2, 26.2, 21.7, 20.3, 14.2, 13.8.

##### (3β,5α,17*E*)-Spiro[3-(methoxymethoxy)-pregn-17(20)-ene-21-oic acid-6,2′[1,3]dioxolane], ethyl ester (37)

Steroid 36 (859 mg, 2.19 mmol) and triethyl phosphonoacetate (4.4 mL, 22 mmol) in anhydrous EtOH (25 mL) under N_2_ was stirred at 35–40 °C. NaOEt solution (sodium 460 mg, 20 mmol in ethanol 20 mL) was slowly added dropwise and then the reaction was refluxed for 16 h. EtOH was removed and water was added. The product was extracted into CH_2_Cl_2_ (2 × 150 mL) and the combined extracts were washed with brine (2 × 50 mL). The extract was dried, filtered, solvents removed and the residue was purified by flash column chromatography (silica gel eluted with 20% EtOAc in hexanes) to give steroid 37 (748 mg, 74%): ^1^H NMR *δ* 5.48 (s, 1H), 4.64 (s, 2H), 4.12–4.07 (q, 2H, *J* = 6.6 Hz), 3.92–3.74 (m, 4H), 3.46–3.44 (m, 1H), 3.32 (s, 3H), 2.80–2.78 (m, 2H), 2.00–0.68 (m, 21H), 0.92 (s, 3H), 0.79 (s, 3H); ^13^C NMR *δ* 175.9, 167.2, 109.2, 108.4, 94.3, 76.0, 65.4, 64.2, 59.4, 55.0, 53.5, 52.9, 50.5, 46.1, 41.1, 38.0, 36.9, 35.0, 33.0, 30.2, 28.1, 26.1, 24.2, 20.8, 18.4, 14.3, 14.1.

##### (3β,5α,17β)-Spiro[3-(methoxymethoxy)-pregnane-21-oic acid-6,2′[1,3]dioxolane], ethyl ester (38)

To a solution of steroid 37 (748 mg, 1.62 mmol) in EtOH (100 mL) in a hydrogenation flask was added PtO_2_ (30 mg) at 23 °C. The flask was evacuated and filled three times with H_2_. The hydrogenation was carried out under 50 psi of H_2_. After 3 h, the reaction mixture was filtered through Celite and washed with EtOAc (200 mL). The solvent was removed and the residue was purified by flash column chromatography (silica gel eluted with 25% EtOAc in hexanes) to afford steroid 38 (650 mg, 87%): ^1^H NMR *δ* 4.63 (s, 2H), 4.09 (q, 2H, *J* = 7.0 Hz), 3.93–3.66 (m, 4H), 3.49–3.33 (m, 1H), 3.31 (s, 3H), 2.33–0.63 (m, 26H), 0.90 (s, 3H), 0.56 (s, 3H); ^13^C NMR *δ* 173.6, 109.4, 94.3, 76.2, 65.4, 64.1, 60.0, 55.0, 54.7, 53.7, 50.7, 46.7, 42.1, 41.3, 38.1, 37.1, 36.9, 35.2, 33.4, 28.2, 28.0, 26.2, 24.4, 20.7, 14.1, 14.0, 12.5.

##### (3β,5α,17β)-2*R*-(3-(Methoxymethoxy)-spiro[androstane-6,2′-[1,3]dioxolan]-17-yl)-5-((benzyl)oxy)pentanoate, ethyl ester (39)

To a solution of steroid 38 (642 mg, 1.38 mmol) in THF (30 mL) was added LDA (1.5 mL, 2.0 M, 3 mmol) and HMPA (4 mmol, 0.72 mL) at −78 °C. After 45 min, ((3-iodopropoxy)methyl)benzene (828 mg, 3 mmol) in THF (5 mL) was added and the reaction was warmed to 23 °C for 16 h. Water was added and the product was extracted into EtOAc (2 × 150 mL). The combined extracts were dried, filtered and the solvents removed. The residue was purified by flash column chromatography (silica gel eluted with 10–25% EtOAc in hexanes) to afford steroid 39 (830 mg, 98%): ^1^H NMR (CDCl_3_) *δ* 7.35–7.28 (m, 5H), 4.70 (s, 2H), 4.49 (d, 2H, *J* = 1.8 Hz), 4.13 (q, 2H, *J* = 7.0 Hz), 3.97–3.77 (m, 4H), 3.51–3.43 (m, 3H), 3.37 (s, 3H), 2.24–0.78 (m, 29H), 0.94 (s, 3H), 0.70 (s, 3H); ^13^C NMR (CDCl_3_) *δ* 175.9, 138.5, 128.3 (2 × C), 127.5 (2 × C), 127.4, 109.5, 94.4, 76.3, 72.7 (2 × C), 69.9, 65.4, 64.2, 59.8, 55.2, 55.1, 53.6, 52.6, 50.7, 47.1, 42.2, 41.3, 38.1, 37.5, 36.9, 33.4, 28.5, 28.3, 27.3, 26.9, 26.2, 23.7, 20.9, 14.2, 12.2.

##### (3β,5α,17β)-2*R*-(3-(Methoxymethoxy)-spiro[androstane-6,2′-[1,3]dioxolan]-17-yl)-5-((benzyl)oxy)pentanol (40)

To a solution of steroid 39 (830 mg, 1.36 mmol) in diethyl ether (50 mL) was added LiAlH_4_ (2.0 M in THF, 5 mL, 10 mmol) at 23 °C. After 2 h, 1.2 M NaOH (2 mmol, 1.7 mL) was slowly added. After stirring for 45 min, the mixture was filter through Celite and washed with CH_2_Cl_2_ (200 mL). Solvent was removed under reduced pressure and the residue was purified by flash column chromatography (silica gel eluted with 35% EtOAc in hexanes) to afford steroid 40 (637 mg, 82%): ^1^H NMR (CDCl_3_) *δ* 7.35–7.28 (m, 5H), 4.69 (s, 2H), 4.51 (s, 2H), 3.95–3.45 (m, 9H), 3.37 (s, 3H), 1.99–0.70 (m, 27H), 0.95 (s, 3H), 0.69 (s, 3H); ^13^C NMR (CDCl_3_) *δ* 138.4, 128.3 (2 × C), 127.6 (2 × C), 127.5, 109.6, 94.4, 76.2, 72.9, 70.8, 65.4, 64.2, 62.5, 55.8, 55.1, 53.6, 50.6, 50.3, 42.3, 42.1, 41.3, 39.1, 38.1, 36.9, 33.3, 28.2, 27.5, 26.2, 25.9, 25.5, 24.0, 21.0, 14.1, 12.2.

##### 17-((*R*)-5-(Benzyloxy)pentan-2-yl)-3((3β,5α,17β)-(methoxymethoxy))-spiro[androstane-6,2′-[1,3]dioxolane] (41)

To a solution of steroid 40 (637 mg, 1.12 mmol) in CH_2_Cl_2_ (30 mL) was added MsCl (0.20 mL, 2.5 mmol) and Et_3_N (0.56 mL, 4 mmol) at 0 °C. After 1 h, aqueous NaHCO_3_ (50 mL) was added and the product was extracted into CH_2_Cl_2_ (2 × 100 mL). The combined extracts were dried, filtered and the solvents removed. To a solution of the crude mesylate (724 mg, 1.12 mmol) in diethyl ether (50 mL) was added LiAlH_4_ (2.0 M in THF, 5 mL, 10 mmol) at 23 °C. After 2 h, 1.2 M NaOH (2 mmol, 1.7 mL) was slowly added. After stirring for 45 min, the mixture was filtered through Celite and the Celite was washed with CH_2_Cl_2_ (250 mL). Solvent was removed and the residue was purified by flash column chromatography (silica gel eluted with 25% EtOAc in hexanes) to afford steroid 41 (600 mg, 97%): ^1^H NMR (CDCl_3_) *δ* 7.35–7.25 (m, 5H), 4.69 (s, 2H), 44.50 (s, 2H), 3.95–3.41 (m, 7H), 3.36 (s, 3H), 1.99–0.72 (m, 29H), 0.94 (s, 3H), 0.67 (s, 3H); ^13^C NMR (CDCl_3_) *δ* 138.7, 128.3 (2 × C), 127.6 (2 × C), 127.4, 109.7, 94.4, 76.3, 72.8, 71.0, 65.5, 64.2, 56.0, 56.0, 55.1, 53.6, 50.7, 42.6, 41.4, 39.8, 38.2, 37.0, 35.5, 33.4, 32.2, 28.3, 28.1, 26.3, 26.2, 24.1, 21.0, 18.6, 14.2, 12.0.

##### (*R*)-4-((3β,5α,17β)-3-(Methoxymethoxy)-spiro[androstane-6,2′-[1,3]dioxolan]-17-yl)pentan-1-ol (42)

To a solution of steroid 41 (600 mg, 1.08 mmol) in EtOAc (100 mL) in a hydrogenation flask was added 10% Pd/C (300 mg) at 23 °C. The flask was evacuated and flushed three times with H_2_. Hydrogenation was carried out at 50 psi of H_2_. After 1 h, the reaction mixture was filtered through Celite and the Celite was washed with EtOAc (200 mL). The solvent was removed and the residue was purified by flash column chromatography (silica gel eluted with 35% EtOAc in hexanes) to afford steroid 42 (470 mg, 93%): ^1^H NMR (CDCl_3_) *δ* 4.69 (s, 2H), 3.97–3.66 (m, 4H), 3.49–3.46 (m, 7H), 3.37 (s, 3H), 1.99–0.65 (m, 26H), 0.95 (s, 3H), 0.68 (s, 3H); ^13^C NMR (100 MHz, CDCl_3_) *δ* 109.6, 94.4, 76.3, 65.5, 64.2, 63.5, 56.0, 56.0, 55.1, 53.6, 50.7, 42.6, 41.4, 39.7, 38.2, 37.0, 35.5, 33.4, 31.8, 29.4, 28.3, 28.1, 26.2, 24.1, 21.0, 18.6, 14.2, 12.0.

##### (3β,5α,17β)-17-((*R*)-5-Hydroxypent-2-yl)-3-(methoxymethoxy)-androstan-6-one (43)

To a solution of the ketal (470 mg, 1 mmol) in acetone (50 mL) and water (0.5 mL) was added *p*-toluenesulfonic acid monohydrate (50 mg) at 23 °C. After 3 h, solid NaHCO_3_ (200 mg) was added. Acetone was removed and the residue was purified by flash column chromatography (silica gel eluted with 30% EtOAc in hexanes) to afford steroid 43 (325 mg, 84%): ^1^H NMR (CDCl_3_) *δ* 4.64–4.56 (m, 2H), 3.52–3.37 (m, 3H), 3.29 (s, 3H), 2.26–0.80 (m, 30H), 0.69 (s, 3H), 0.60 (s, 3H); ^13^C NMR (100 MHz, CDCl_3_) *δ* 210.7, 94.2, 75.1, 63.1, 56.6, 56.5, 55.8, 55.0, 53.7, 46.5, 42.8, 40.9, 39.3, 37.7, 36.5, 35.3, 31.7, 29.2, 28.0, 27.9, 26.8, 23.8, 21.3, 18.4, 12.9, 11.9.

##### (*R*)-4-((3β,5α,17β)-3-(Methoxymethoxy)-spiro[androstane-6,3′-diazirin]-17-yl)pentan-1-ol (44)

Using the procedure described for the preparation of compound 11, steroid 43 (375 mg, 0.89 mmol) was converted to diazirine 44 (325 mg, 84%). Purification was by flash column chromatography (silica gel eluted with 25–40% EtOAc in hexanes): ^1^H NMR (CDCl_3_) *δ* 4.56 (s, 2H), 3.59 (q, 2H, *J* = 5.2 Hz), 3.54–3.23 (m, 1H), 3.28 (s, 3H), 2.02–0.73 (m, 28H), 1.12 (s, 3H), 0.68 (s, 3H), 0.49–0.36 (m, 2H); ^13^C NMR (CDCl_3_) *δ* 94.3, 75.9, 63.4, 56.0, 55.9, 55.1, 53.6, 45.2, 42.6, 39.6, 37.6, 37.3, 36.3, 35.4, 33.8, 31.7, 30.2, 29.3, 29.3, 28.0, 27.9, 23.8, 21.1, 18.6, 12.8, 12.0.

##### (*R*)-4-((3β,5α,17β)-3-Hydroxy-spiro[androstane-6,3′-diazirin]-17-yl)pentan-1-ol, 1-sulfate ammonium salt (MQ271)

Using the sulfation procedure described for the preparation of KK238, diazirine 44 (325 mg, 0.75 mmol) was converted to MQ271 (287 mg, 79%). Purification was by flash column chromatography (silica gel eluted with 10–25% MeOH in CH_2_Cl_2_): ^1^H NMR (CD_3_OD) *δ* 3.91 (t, 2H, *J* = 6.4 Hz), 3.42–2.29 (m, 1H), 2.10–0.64 (m, 32H), 1.08 (s, 3H), 0.68 (s, 3H), 0.37–0.26 (m, 2H); ^13^C NMR (CD_3_OD) *δ* 71.7, 68.8, 57.6, 57.4, 55.1, 46.5, 44.0, 41.2, 38.8, 38.5, 37.6, 36.9, 35.3, 34.1, 33.2, 31.5, 29.2, 27.2, 25.1, 22.4, 19.2, 13.5, 12.6.

##### (3β,5α,17β)-2*R*-(3-(Methoxymethoxy)-spiro[androstanane-6,2′-[1,3]dioxolan]-17-yl)-5-((triisopropylsilyl)oxy)pentanoate, ethyl ester (45)

To a solution of steroid 38 (650 mg, 1.4 mmol) in THF (30 mL) was added LDA (2.1 mL, 2.0 M, 4.2 mmol) and HMPA (5 mmol, 0.90 mL) at −78 °C. After 45 min, (3-iodopropoxy)(triisopropyl)silane (1.44 g, 4.2 mmol) in THF (5 mL) was added and the reaction was warmed to 23 °C for 16 h. Water was added and the product was extracted into EtOAc (2 × 150 mL). The combined extracts were dried, filtered, solvents removed and the residue was purified by flash column chromatography (silica gel eluted with 25% EtOAc in hexanes) to afford steroid 45 (902 mg, 95%): ^1^H NMR *δ* 4.63 (s, 2H), 4.10 (q, 2H, *J* = 7.4 Hz), 3.90–3.81 (m, 3H), 3.78–3.56 (m, 3H), 3.48–3.33 (m, 1H), 3.31 (s, 3H), 2.22–0.61 (m, 32H), 1.00 (s, 18H), 0.88 (s, 3H), 0.66 (s, 3H); ^13^C NMR *δ* 175.9, 109.4, 94.3, 76.2, 65.4, 64.2, 62.8, 59.6, 55.2, 55.0, 53.5, 52.5, 50.6, 47.0, 42.1, 41.3, 38.0, 37.4, 36.9, 33.3, 30.4, 28.2, 26.8, 26.1, 23.6, 20.8, 17.9 (7 × C), 14.1 (2 × C), 12.1, 11.8 (3 × C).

##### (3β,5α,17β)-2*R*-(3-(Methoxymethoxy)-spiro[androstanane-6,2′-[1,3]dioxolan]-17-yl)-5-((triisopropylsilyl)oxy)pentanol (46)

To a solution of the steroid 45 (902 mg, 1.33 mmol) in diethyl ether (60 mL) was added LiAlH_4_ (2.0 M in THF, 4 mL, 8 mmol) at 23 °C. After 2 h, 1.2 M NaOH (1.6 mmol, 1.33 mL) was slowly added. After stirring for 45 min, the mixture was filter through Celite and the Celite was washed with CH_2_Cl_2_ (200 mL). Solvent was removed and the residue was purified by flash column chromatography (silica gel eluted with 40% EtOAc in hexanes) to afford steroid 46 (813 mg, 96%): ^1^H NMR *δ* 4.66 (s, 2H), 3.93–3.35 (m, 9H), 1.96–0.64 (m, 30H), 1.04 (s, 21H), 0.91 (s, 3H), 0.67 (s, 3H); ^13^C NMR *δ* 109.6, 94.3, 76.2, 65.4, 64.2, 63.7, 62.8, 55.8, 55.1, 53.6, 50.6, 50.4, 42.3, 42.0, 41.3, 39.1, 38.1, 36.9, 33.3, 28.9, 28.2, 27.4, 26.2, 25.1, 24.0, 21.0, 18.0 (6 × C), 14.1, 12.2, 11.9 (3 × C).

##### Triisopropyl(((R)-4-((3β,5α,17β)-3-(methoxymethoxy)-spiro[androstane-6,2′[1,3]dioxolan]-17-yl)hex-5-yn-1-yl)oxy)silane (47)

To a solution of steroid 46 (813 mg, 1.28 mmol) in CH_2_Cl_2_ (30 mL) was added NaHCO_3_ (800 mg) and Dess–Martin periodinane (1.27 g, 3 mmol) at 23 °C. After 2 h, water was added and the product was extracted into CH_2_Cl_2_ (2 × 100 mL). The solvent was removed and the residue was purified by flash chromatography (silica gel, eluted with 25% EtOAc in hexanes) to give an aldehyde intermediate (687 mg, 1.08 mmol) which was dissolved in MeOH/THF (6 mL/6 mL) and (dimethyl-1-diazo-2-oxopropyl)phosphonate (0.46 mL, 3 mmol) and K_2_CO_3_ (437 mg, 4 mmol) were added at 23 °C. After 48 h, water was added and the product was extracted into EtOAc (3 × 100 mL). The solvent was removed and the residue was purified by flash column chromatography (silica gel eluted with 10–25% EtOAc in hexanes) to give recovered aldehyde (120 mg) and steroid 47 (438 mg, 64%): ^1^H NMR *δ* 4.68 (s, 2H), 3.95–3.37 (m, 7H), 3.35 (s, 3H), 2.23–0.66 (m, 30H), 1.05 (s, 18H), 0.94 (s, 3H), 0.73 (s, 3H); ^13^C NMR *δ* 109.5, 94.3, 87.5, 76.2, 70.4, 65.4, 64.2, 63.1, 55.6, 55.0, 53.7, 53.3, 50.7, 42.7, 41.4, 38.8, 38.1, 36.9, 33.4, 32.7, 30.1, 29.9, 28.3, 27.8, 26.2, 23.7, 20.8, 18.0 (6 × C), 14.1, 12.3, 11.9 (3 × C).

##### (*R*)-4-((3β,5α,17β)-3-(Methoxymethoxy)-spiro[androstane-6,2′-[1,3]dioxolan]-17-yl)hex-5-yn-1-ol (48)

To a solution of steroid 47 (438 mg, 0.70 mmol) in THF (10 mL) was added TBAF (2 mL, 1.0 M in THF, 2.0 mmol) at 23 °C. After 4 h, THF was removed and the residue was purified by flash column chromatography (silica gel eluted with 30% EtOAc in hexanes) to give steroid 48 (330 mg, ∼100%): ^1^H NMR *δ* 4.62 (s, 2H), 3.89–3.31 (m, 7H), 3.29 (s, 3H), 2.48–0.61 (m, 28H), 0.88 (s, 3H), 0.68 (s, 3H); ^13^C NMR *δ* 109.4, 94.2, 87.2, 76.2, 70.5, 65.3, 64.1, 62.3, 55.5, 54.9, 53.5, 53.1, 50.5, 42.6, 41.3, 38.7, 38.0, 36.8, 33.2, 32.7, 30.0, 29.7, 28.1, 27.8, 26.0, 23.6, 14.0, 13.8, 12.2.

##### (3β,5α,17β)-17-((*R*)-6-Hydroxyhex-1-yn-3-yl)-3-(methoxymethoxy)-androstan-6-one (49)

To a solution of the steroid 48 (320 mg, 0.68 mmol) in acetone/water (50 mL/0.5 mL) was added *p*-toluenesulfonic acid (100 mg) at 23 °C. After 3 h, solid NaHCO_3_ (200 mg) was added, solvent was removed and the residue was purified by flash column chromatography (silica gel eluted with 10–25% EtOAc in hexanes) to give steroid 49 (278 mg, 96%): ^1^H NMR *δ* 4.65 (q, 2H, *J* = 6.6 Hz), 3.61 (t, 2H, *J* = 6.2 Hz), 3.46–3.39 (m, 1H), 3.31 (s, 3H), 2.28–0.66 (m, 28H), 0.71 (s, 3H), 0.68 (s, 3H); ^13^C NMR *δ* 210.7, 94.2, 87.1, 75.1, 70.7, 62.4, 56.6, 56.2, 55.0, 53.9, 53.1, 46.5, 43.0, 40.9, 38.4, 37.7, 36.5, 32.7, 30.0, 29.7, 28.0, 27.7, 26.8, 23.4, 21.1, 12.9, 12.2.

##### (*R*)-4-((3β,5α,17β)-3-(Methoxymethoxy)-spiro[androstane-6,3′-diazirin]-17-yl)hex-5-yn-1-ol (50)

Using the procedure described for the preparation of compound 11, steroid 49 (278 mg, 0.65 mmol) was converted to diazirine 50 (203 mg, 73%). Reaction times: step 1, 72 h; step 2, 20 min. Purification was by flash column chromatography (silica gel eluted with 25–40% EtOAc in hexanes): ^1^H NMR *δ* 4.59 (s, 2H), 3.69 (t, 2H, *J* = 6.2 Hz), 3.40–3.33 (m, 1H), 3.31 (s, 3H), 2.47–0.74 (m, 26H), 1.15 (s, 3H), 0.77 (s, 3H), 0.51–0.39 (m, 2H); ^13^C NMR *δ* 94.4, 87.3, 75.7, 70.8, 62.8, 55.6, 55.1, 53.8, 53.2, 45.3, 42.8, 38.8, 37.7, 37.4, 36.3, 33.8, 32.9, 30.3, 30.0, 29.9, 29.4, 28.0, 27.9, 23.4, 21.0, 12.9, 12.3.

##### (*R*)-4-((3β,5α,17β)-3-Hydroxy-spiro[androstane-6,3′-diazirin]-17-yl)hex-5-yn-1-ol, 1-sulfate ammonium salt (MQ273)

Using the sulfation procedure described for the preparation of KK238 followed by removal of the MOM protecting group using 6 N HCl in THF at 23 °C for 2 h, converted diazirine 50 (148 mg, 0.34 mmol) to MQ273 (137 mg, 83%). Purification was by flash column chromatography (silica gel eluted with 10–25% MeOH in CH_2_Cl_2_): ^1^H NMR (400 MHz, CD_3_OD) *δ* 3.96 (q, 2H, *J* = 6.2 Hz), 3.37–2.31 (m, 1H), 2.47–0.60 (m, 30H), 1.09 (s, 3H), 0.74 (s, 3H), 0.36–0.26 (m, 2H); ^13^C NMR (100 MHz, CD_3_OD) *δ* 87.9, 72.6, 71.6, 69.0, 57.1, 55.2, 54.9, 50.0, 46.5, 44.1, 40.4, 38.8, 38.6, 37.6, 35.3, 34.0, 31.5, 31.4, 30.3, 29.0, 27.8, 24.7, 22.3, 13.6, 12.9.

#### Physiological methods

##### Materials

GABA, NMDA and salts were purchased from Sigma-Aldrich (St. Louis, MO). Channel blockers NBQX, D-APV and gabazine were from Tocris Bioscience (Minneapolis, MN).

##### Rat hippocampal cultures

Rat primary cultures of hippocampal cells were prepared from 1 to 3 days postnatal Sprague Dawley rats, as described previously.^22^ Tissue was prepared according to protocols approved by the Institutional Animal Care and Use Committee.

##### Whole-cell patch-clamp recording in cell cultures

Experiments on hippocampal neurons were conducted using standard whole-cell techniques. The bath solution contained (in mM): 138 NaCl, 4 KCl, 2 CaCl_2_, 1 MgCl_2_, 10 glucose, and 10 HEPES; pH 7.25 with NaOH. Patch pipettes were filled with an internal solution containing (in mM): 130 CsCl, 4 NaCl, 4 MgCl_2_, 0.5 CaCl_2_, 5 EGTA, 10 HEPES, pH 7.25 with CsOH. When filled with this solution, pipette open tip resistance was 3–6 MΩ. Cells were clamped at −70 mV. Agonist was applied alone or was co-applied with putative modulator with a multibarrel, gravity-driven local perfusion system. Agonist-alone trials were interleaved with co-applications to ensure reversibility. Culture recordings were performed at room temperature.

Currents were recorded under voltage clamp with an Axopatch 200B amplifier (Molecular Devices, San Jose, CA) and were filtered at 2 kHz and recorded at 5 kHz using pClamp 9.0 software (Clampex, Molecular Devices). Data were analyzed with Clampfit 9.0 (Molecular Devices). The peak current was measured to estimate potentiation, and the ending current was measured to estimate inhibition. Data are presented normalized to the preceding agonist-alone trial and are given in the text and figures as mean ± SEM.

#### Photolabeling methods

##### Cell culture and membrane preparation

HEK293 cells expressing high density human α_1_-8x His-FLAG and human β_3_ GABA_A_ receptors were generated as previously described.^10^ Full-length rat GluN1_FLAG_/GluN2B receptors were expressed in GNTI^−^ cells using the BacMam expression system.^23^ HEK cell membranes were prepared as previously described (Chen *et al.* 2019). Briefly, HEK cells at 80% confluency were harvested in phosphate-buffered saline (PBS) containing proteinase inhibitors. The cells were homogenized with a glass mortar and Teflon pestle and the cell membranes were collected as a pellet after centrifugation at 34 000*g* for 30 minutes at 4 °C. The membranes were resuspended in 10 mM potassium phosphate and 100 mM KCl and stored at −80 °C.

##### Photolabeling and cycloaddition reactions

HEK cell membranes were resuspended at 1.25 mg mL^−1^ in 10 mM potassium phosphate and 100 mM KCl. Photolabeling reagents were added to the membrane suspensions and incubated on ice for 1 hour before UV light exposure. Photolabeling was performed as previously described.^24^ The photolabeled membranes were collected by centrifugation at 21 000*g* for 15 min. Membranes were then solubilized in 2% SDS/PBS for two hours at room temperature. Following centrifugation at 21 000*g* for 30 minutes, the supernatant (protein lysate) containing solubilized membrane protein was collected. TAMRA or MQ112 (biotin-azide linker) was attached to the photolabeled proteins by incubation overnight at room temperature in PBS buffer containing 2% SDS, TAMRA or MQ112, 2.5 mM sodium ascorbate, 250 μM Tris[(1-benzyl-1*H*-1,2,3triazol4-yl)methyl]amine, and 2.5 mM CuSO4. TAMRA and MQ112 were added to the incubations at a 3 : 1 molar ratio to the concentration of the PAL used for labeling.

##### Enrichment of photolabeled proteins

Photolabeled proteins coupled to MQ112 were enriched by diluting SDS to 0.5% and then loading the lysate onto a column containing streptavidin agarose beads. The column was washed with 0.5% SDS/PBS and photolabeled proteins were eluted by cleaving the linker with 100 mM sodium dithionite/0.5% SDS/PBS. The yellow color of MQ112 was eliminated following dithionite elution, indicating complete cleavage of the linker. The eluted proteins were concentrated to 100 μL using a 30 kDa cutoff Centricon filter. The concentrated proteins were analyzed by SDS-PAGE followed by western blot with an anti-Flag antibody.

## Data availability

ESI materials† contain the carbon-13 and proton NMR spectra of the 10 reported photoaffinity labeling reagents.

## Author contributions

DFC and SM conceptualized the study. DFC, SM and ASE designed and had oversight for the experiments. MQ and YX carried out the chemical syntheses and compound characterizations. H-JS performed the electrophysiology experiments. Z-WC and LW performed the photolabeling experiments. DFC wrote the original manuscript. SM, AES and CFZ edited the manuscript. All authors contributed to the final version of the manuscript.

## Conflicts of interest

CFZ is a member of the Scientific Advisory Board of Sage Therapeutics and has equity in the company. DFC has equity in Sage Therapeutics and receives income from a license agreement between Washington University in St. Louis and Sage Therapeutics. Sage Therapeutics was not involved in this study. The other authors have no conflicts to declare.

## Supplementary Material

RA-014-D4RA07074G-s001
