# Supplementary material for: Synthesis and evaluation of photoaffinity labeling reagents for identifying binding sites of sulfated neurosteroids on NMDA and GABAA receptors
Source: RSC Adv. 2024 Nov 13;14(49):36352–69. doi: 10.1039/d4ra07074g (PMC11559520; doi:10.1039/d4ra07074g)
Supplement: RA-014-D4RA07074G-s001 [file RA-014-D4RA07074G-s001.pdf]

Supplementary Information (SI) for RSC Advances.
This journal is © The Royal Society of Chemistry 2024

Supplemental Information

Synthesis and evaluation of photoaffinity labeling reagents for identifying binding sites of sulfated neurosteroids on NMDA and $GABA_A$ receptors.

Mingxing Qian,[a] Yuanjian Xu,[a] Hong-Jin Shu,[b] Zi-Wei Chen,[c,d] Lei Wang,[cef] Charles F. Zorumski,[bd] Alex S. Evers,[acd] Steven Mennerick,[bd] Douglas F. Covey[*abcd]

[a]*Department of Developmental Biology, Washington University in St. Louis, 660 S. Euclid Ave., St. Louis, MO, 63110, USA*

[b]*Department of Psychiatry, Washington University in St. Louis, 660 S. Euclid Ave., St. Louis, MO, 63110, USA*

[c]*Department of Anesthesiology, Washington University in St. Louis, 660 S. Euclid Ave., St. Louis, MO, 63110, USA*

[d]*Taylor Family Institute for Innovative Psychiatric Research, Washington University in St. Louis, 660 S. Euclid Ave., St. Louis, MO, 63110, USA*

[e]*Department of Anesthesiology, Union Hospital, Tongji Medical College, Huazhong University of Science and Technology, Wuhan, 430022, China*

[f]*Key Laboratory of Anesthesiology and Resuscitation (Huazhong University of Science and Technology), Ministry of Education, China*

Table of Contents

^{1}H NMR (400 MHz) spectra for compounds **KK238, YX33, MQ189, MQ231, MQ234, MQ235, MQ236, MQ237, MQ271, MQ273**………………………..…..S2-S11

^{13}C NMR (100 MHz) spectra for compounds **KK238, YX33, MQ189, MQ231, MQ234, MQ235, MQ236, MQ237, MQ271, MQ273**…………………………..S12-S21

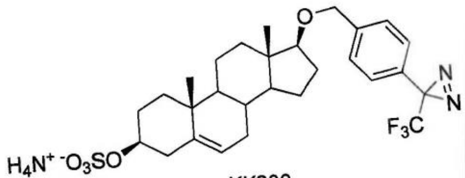

KK238

INDEX	FREQUENCY	PPM	HEIGHT	INDEX	FREQUENCY	PPM	HEIGHT	INDEX	FREQUENCY	PPM	HEIGHT
1	2969.8	7.434	29.4	35	923.9	2.313	3.8	69	584.5	1.463	4.1
2	2961.6	7.414	35.6	36	827.7	2.072	7.1	70	526.9	1.319	7.7
3	2881.1	7.212	30.8	37	822.7	2.059	7.2	71	521.0	1.304	7.2
4	2872.9	7.192	25.4	38	819.2	2.051	9.8	72	514.4	1.288	8.9
5	2190.5	5.484	12.1	39	814.1	2.038	11.6	73	509.0	1.274	11.1
6	2150.8	5.384	13.1	40	807.1	2.020	7.1	74	502.7	1.258	3.7
7	2145.8	5.371	12.7	41	804.8	2.015	7.1	75	489.9	1.226	5.1
8	1951.1	4.884	408.4	42	800.5	2.004	9.6	76	485.2	1.215	5.2
9	1844.1	4.616	6.4	43	795.1	1.990	6.5	77	477.4	1.195	8.6
10	1816.8	4.548	68.9	44	791.9	1.982	6.9	78	472.7	1.183	8.3
11	1804.0	4.516	4.2	45	787.7	1.972	10.6	79	465.0	1.164	4.8
12	1662.7	4.162	5.0	46	784.2	1.963	13.2	80	459.9	1.151	4.3
13	1656.1	4.146	6.1	47	780.6	1.954	9.7	81	454.5	1.138	5.3
14	1651.0	4.133	10.0	48	775.2	1.941	8.6	82	451.0	1.129	5.9
15	1646.4	4.121	6.0	49	771.7	1.932	10.7	83	440.4	1.103	8.9
16	1639.7	4.105	5.3	50	768.2	1.923	8.4	84	436.9	1.094	8.7
17	1382.0	3.460	9.2	51	763.1	1.910	9.5	85	426.8	1.068	5.1
18	1373.9	3.439	15.7	52	760.0	1.903	5.7	86	422.9	1.059	5.2
19	1365.3	3.418	8.4	53	753.4	1.886	6.1	87	419.4	1.050	4.2
20	1334.9	3.342	4.9	54	749.9	1.877	9.8	88	412.4	1.032	132.3
21	1320.9	3.307	29.1	55	746.4	1.868	5.2	89	405.0	1.014	7.8
22	1319.4	3.303	38.7	56	656.5	1.643	9.4	90	399.6	1.000	7.5
23	1317.8	3.299	28.7	57	653.0	1.635	8.0	91	394.9	0.989	9.7
24	1020.4	2.554	5.2	58	644.0	1.612	13.4	92	391.4	0.980	9.7
25	1018.5	2.550	5.7	59	640.5	1.603	13.9	93	385.9	0.966	9.3
26	1015.8	2.543	5.8	60	637.4	1.596	11.6	94	382.1	0.956	8.4
27	1013.8	2.538	5.3	61	632.7	1.584	12.5	95	374.3	0.937	5.2
28	1007.2	2.521	7.3	62	627.7	1.571	17.8	96	369.2	0.924	3.9
29	1005.3	2.516	7.9	63	624.2	1.562	19.1	97	332.6	0.833	130.9
30	1002.5	2.510	7.8	64	619.9	1.552	13.2				
31	1000.6	2.505	6.9	65	613.7	1.536	21.3				
32	948.8	2.375	5.1	66	604.3	1.513	24.0				
33	946.9	2.370	5.0	67	598.1	1.497	14.3				
34	935.6	2.342	8.3	68	594.2	1.487	12.5				

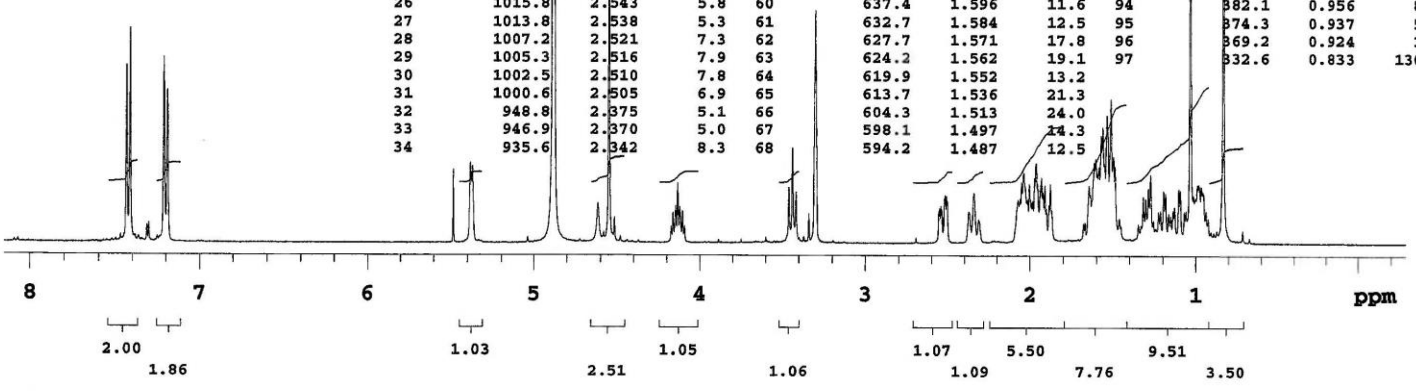

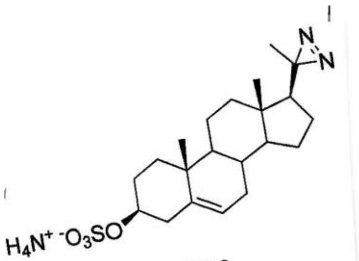

YX33

$C_{21}H_{35}N_2O_4S$

13 mg
7 mg

INDEX	FREQUENCY	PPM	HEIGHT	INDEX	FREQUENCY	PPM	HEIGHT
1	2141.2	5.360	8.3	35	592.4	1.483	6.1
2	2136.6	5.348	8.0	36	579.9	1.452	4.8
3	1945.4	4.870	132.3	37	575.3	1.440	4.7
4	1644.2	4.116	3.9	38	569.4	1.425	4.7
5	1639.5	4.104	5.7	39	564.7	1.414	5.4
6	1634.4	4.091	3.8	40	557.0	1.394	4.3
7	1463.9	3.665	3.3	41	553.1	1.384	5.4
8	1312.9	3.287	35.3	42	548.8	1.374	4.1
9	1150.2	2.879	15.0	43	543.7	1.361	4.3
10	1009.7	2.527	3.9	44	539.1	1.349	3.9
11	1006.6	2.520	3.9	45	488.1	1.222	3.4
12	996.4	2.494	5.4	46	483.4	1.210	3.4
13	993.7	2.488	5.1	47	475.6	1.191	5.8
14	937.7	2.347	3.4	48	470.9	1.179	6.2
15	926.4	2.319	5.4	49	463.5	1.160	3.9
16	821.3	2.056	4.4	50	449.9	1.126	3.9
17	806.1	2.018	7.3	51	446.4	1.118	4.6
18	803.4	2.011	7.4	52	436.3	1.092	7.0
19	790.1	1.978	6.5	53	432.4	1.082	8.7
20	787.0	1.970	5.5	54	425.8	1.066	5.2
21	780.8	1.955	4.4	55	419.6	1.050	9.4
22	763.3	1.911	8.1	56	407.9	1.021	71.1
23	760.2	1.903	9.5	57	396.2	0.992	7.6
24	746.5	1.869	6.2	58	391.5	0.980	8.5
25	667.9	1.672	5.4	59	383.4	0.960	75.8
26	657.8	1.647	11.1	60	375.2	0.939	11.3
27	648.0	1.622	10.3	61	363.5	0.910	7.0
28	636.4	1.593	9.4	62	344.4	0.862	61.9
29	625.5	1.566	14.7				
30	622.7	1.559	15.5				
31	613.0	1.535	9.2				
32	609.5	1.526	9.5				
33	600.9	1.504	5.7				
34	595.1	1.490	6.3				

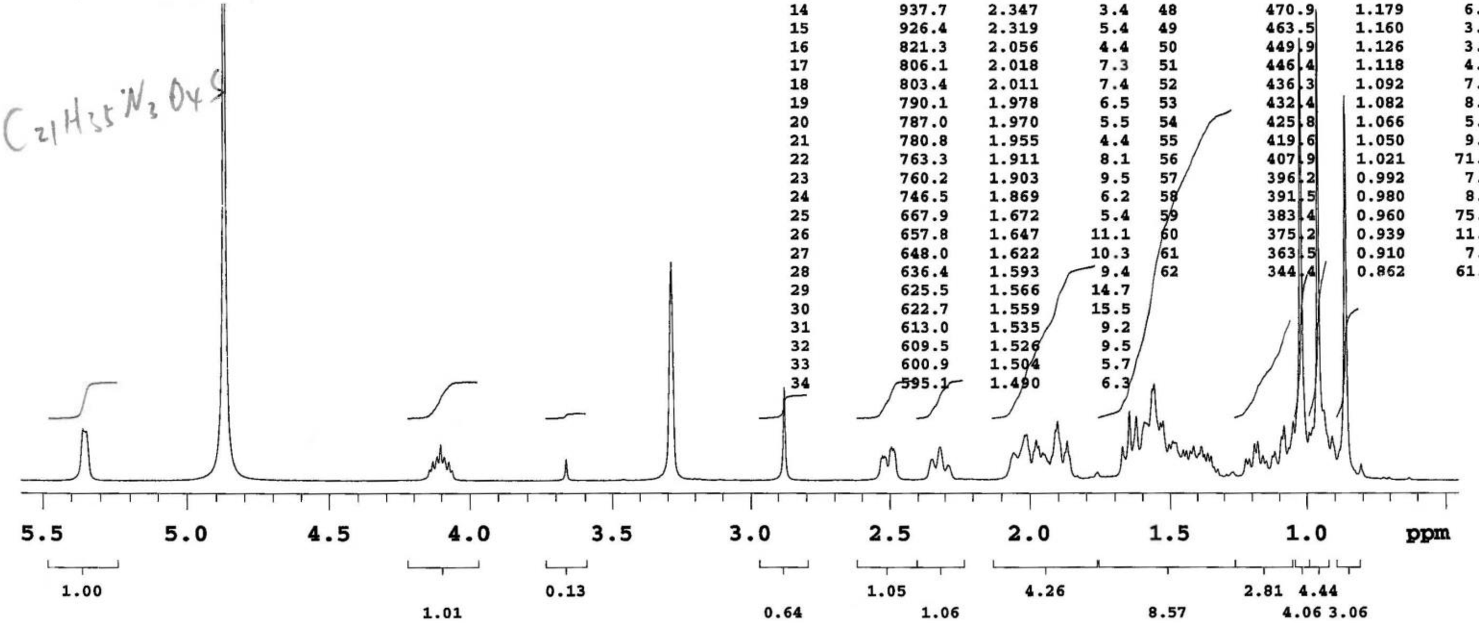

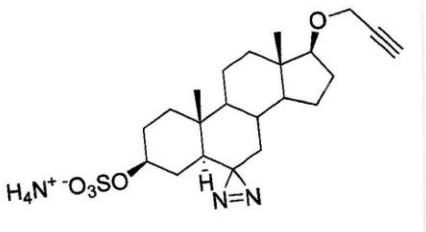

MQ189

INDEX	FREQUENCY	PPM	HEIGHT	INDEX	FREQUENCY	PPM	HEIGHT
1	1946.8	4.870	64.1	38	516.0	1.291	18.6
2	1685.0	4.215	11.6	39	509.3	1.274	36.8
3	1678.4	4.199	11.1	40	501.9	1.255	19.7
4	1670.9	4.180	44.3	41	494.4	1.237	13.8
5	1664.7	4.164	44.9	42	479.6	1.200	153.9
6	1656.9	4.145	16.0	43	468.2	1.171	19.6
7	1649.4	4.126	17.3	44	465.1	1.163	18.1
8	1642.4	4.109	4.9	45	454.9	1.138	15.3
9	1494.1	3.738	23.9	46	449.1	1.123	12.9
10	1450.3	3.628	11.4	47	444.4	1.112	12.3
11	1442.4	3.608	22.0	48	437.3	1.094	11.1
12	1433.8	3.587	11.9	49	418.9	1.048	12.5
13	1352.1	3.382	24.4	50	406.0	1.016	13.4
14	1337.2	3.345	14.8	51	392.3	0.981	7.8
15	1125.5	2.816	27.3	52	389.6	0.975	8.4
16	835.2	2.089	3.5	53	386.5	0.967	8.4
17	819.2	2.049	51.9	54	377.9	0.945	14.1
18	812.1	2.032	16.7	55	366.9	0.918	7.8
19	806.7	2.018	18.6	56	333.6	0.835	141.4
20	790.6	1.978	21.2	57	236.6	0.592	6.7
21	776.9	1.944	15.1	58	224.5	0.562	15.4
22	765.2	1.914	4.0	59	212.0	0.530	14.9
23	760.5	1.902	4.4	60	199.0	0.498	5.6
24	748.8	1.873	23.4	61	173.6	0.434	13.7
25	732.3	1.832	27.9	62	169.3	0.424	13.3
26	716.3	1.792	14.2	63	159.5	0.399	13.1
27	678.7	1.698	9.5	64	155.6	0.389	12.4
28	667.0	1.669	13.4				
29	624.3	1.562	14.6				
30	611.4	1.530	35.1				
31	599.3	1.499	44.6				
32	591.1	1.479	34.5				
33	579.3	1.449	12.6				
34	544.1	1.361	12.4				
35	537.9	1.346	12.8				
36	528.9	1.323	11.7				
37	522.2	1.306	10.4				

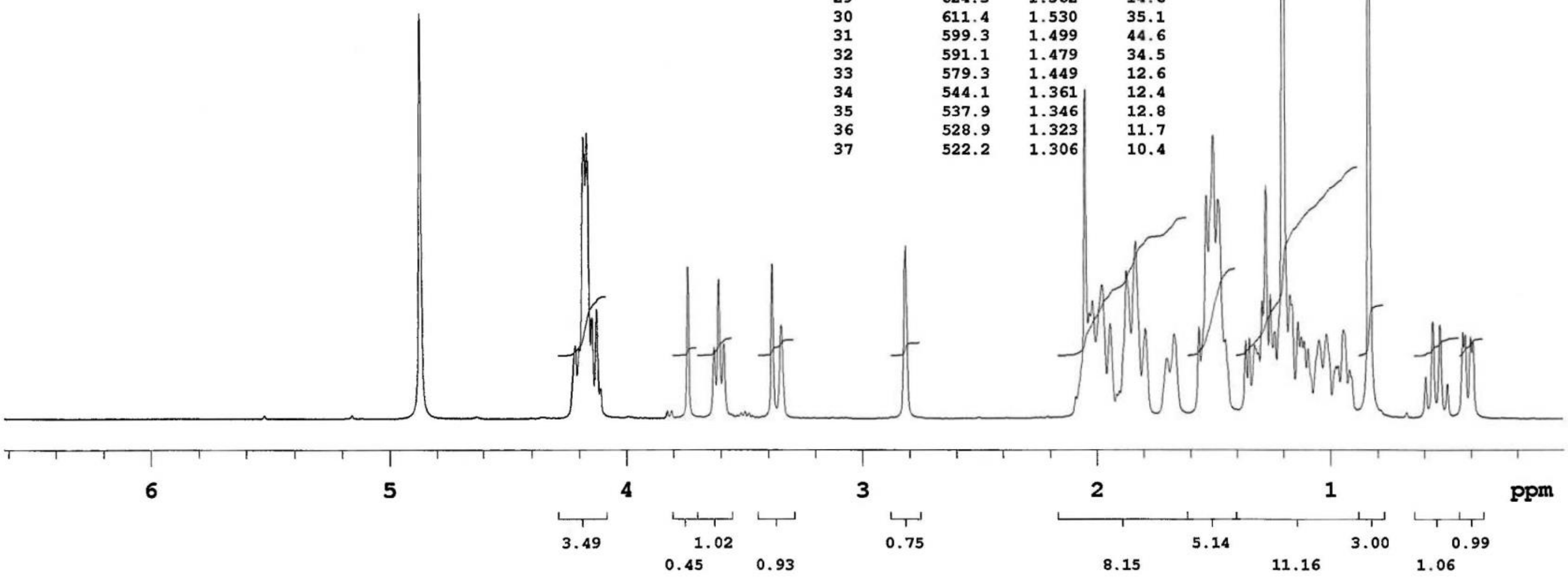

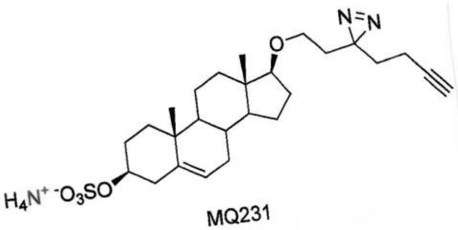

MQ231

INDEX	FREQUENCY	PPM	HEIGHT	INDEX	FREQUENCY	PPM	HEIGHT	INDEX	FREQUENCY	PPM	HEIGHT
1	2171.0	5.431	17.3	37	810.6	2.028	13.3	73	463.1	1.159	12.3
2	2166.3	5.419	16.4	38	807.1	2.019	12.7	74	459.6	1.150	11.8
3	2063.8	5.163	2.0	39	801.2	2.004	15.1	75	449.5	1.124	6.9
4	1946.8	4.870	111.0	40	797.7	1.995	14.6	76	445.9	1.116	6.6
5	1688.9	4.225	3.2	41	784.8	1.963	23.0	77	432.6	1.082	149.2
6	1684.2	4.213	6.1	42	771.1	1.929	13.0	78	424.0	1.061	11.5
7	1677.6	4.197	7.5	43	690.5	1.727	4.7	79	418.9	1.048	14.5
8	1672.9	4.185	12.2	44	686.9	1.718	4.9	80	413.8	1.035	14.9
9	1667.8	4.172	7.8	45	673.2	1.684	43.9	81	408.4	1.022	15.0
10	1661.2	4.156	6.5	46	665.8	1.666	69.1	82	402.1	1.006	13.0
11	1656.5	4.144	3.3	47	658.0	1.646	61.9	83	386.1	0.966	4.0
12	1478.4	3.698	2.6	48	651.3	1.629	67.4	84	379.8	0.950	5.3
13	1354.0	3.387	16.4	49	645.5	1.615	44.9	85	373.5	0.934	4.9
14	1339.9	3.352	104.3	50	632.9	1.583	19.7	86	337.9	0.845	150.3
15	1338.8	3.349	100.6	51	623.2	1.559	40.1				
16	1334.9	3.339	75.9	52	616.1	1.541	21.2				
17	1331.3	3.330	33.7	53	612.6	1.532	22.3				
18	1328.6	3.324	33.5	54	602.0	1.506	9.7				
19	1323.1	3.310	14.0	55	587.6	1.470	2.5				
20	1039.1	2.599	7.4	56	581.3	1.454	2.5				
21	1036.3	2.592	7.7	57	571.9	1.431	2.4				
22	1026.2	2.567	10.5	58	564.9	1.413	2.8				
23	1023.0	2.559	10.5	59	552.7	1.383	6.4				
24	968.2	2.422	7.3	60	546.5	1.367	8.2				
25	955.3	2.390	11.5	61	541.0	1.353	16.0				
26	943.6	2.360	5.4	62	534.4	1.337	16.6				
27	903.7	2.261	17.8	63	528.9	1.323	27.0				
28	901.3	2.255	30.9	64	522.6	1.307	11.7				
29	898.6	2.248	17.7	65	513.2	1.284	9.6				
30	848.5	2.123	8.6	66	509.7	1.275	9.5				
31	836.4	2.092	26.3	67	500.7	1.253	12.0				
32	833.7	2.085	26.3	68	496.4	1.242	11.3				
33	829.0	2.074	41.2	69	488.2	1.221	6.5				
34	826.2	2.067	37.8	70	483.5	1.209	5.9				
35	821.1	2.054	31.8	71	477.2	1.194	7.6				
36	818.8	2.048	29.4	72	473.7	1.185	8.0				

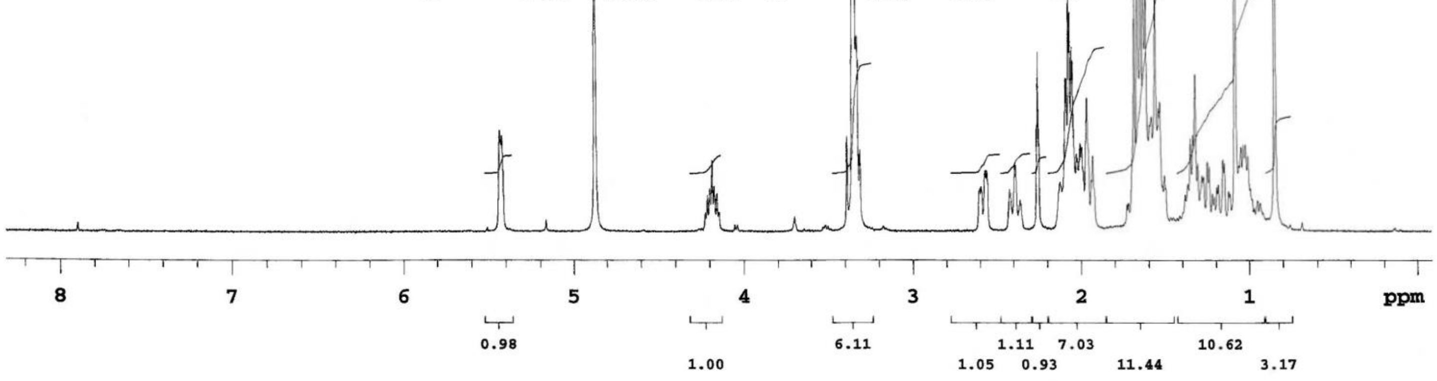

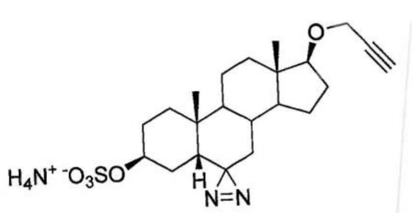

MQ234

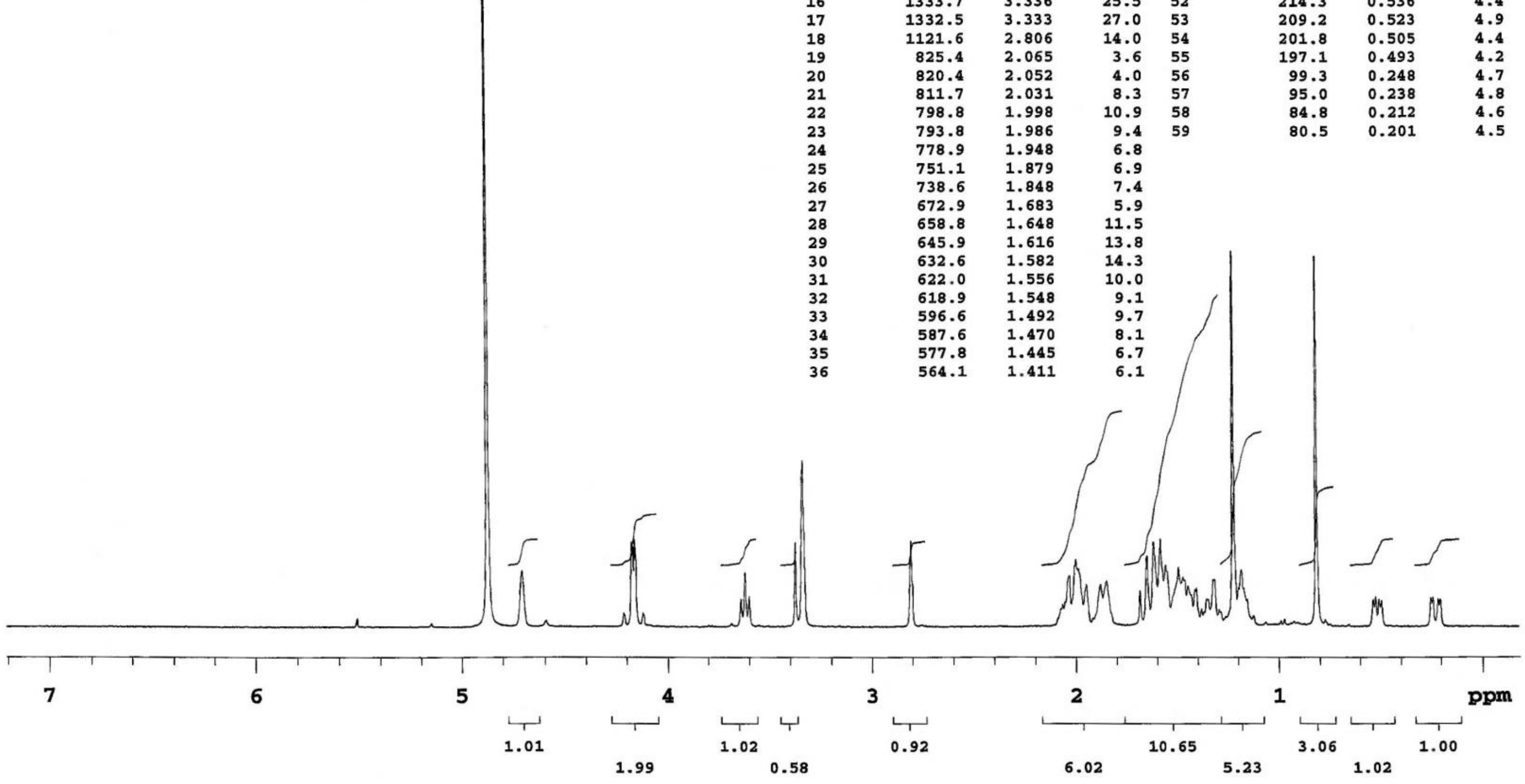

INDEX	FREQUENCY	PPM	HEIGHT	INDEX	FREQUENCY	PPM	HEIGHT
1	2204.2	5.514	1.5	37	561.3	1.404	6.3
2	1946.8	4.870	150.3	38	550.8	1.378	2.8
3	1881.0	4.706	9.2	39	540.6	1.352	4.5
4	1835.3	4.591	1.0	40	537.1	1.344	4.5
5	1684.6	4.214	2.2	41	525.4	1.314	7.7
6	1682.3	4.208	1.9	42	516.0	1.291	2.8
7	1668.6	4.174	13.8	43	488.2	1.221	61.2
8	1666.2	4.168	13.1	44	478.4	1.197	6.4
9	1662.7	4.159	14.2	45	472.5	1.182	9.3
10	1660.8	4.155	11.8	46	467.4	1.169	6.3
11	1647.1	4.120	2.3	47	461.2	1.154	4.4
12	1455.0	3.640	4.5	48	448.7	1.122	1.8
13	1446.7	3.619	8.8	49	388.0	0.971	1.2
14	1438.5	3.599	4.9	50	325.4	0.814	60.3
15	1347.8	3.372	13.7	51	309.0	0.773	1.1
16	1333.7	3.336	25.5	52	214.3	0.536	4.4
17	1332.5	3.333	27.0	53	209.2	0.523	4.9
18	1121.6	2.806	14.0	54	201.8	0.505	4.4
19	825.4	2.065	3.6	55	197.1	0.493	4.2
20	820.4	2.052	4.0	56	99.3	0.248	4.7
21	811.7	2.031	8.3	57	95.0	0.238	4.8
22	798.8	1.998	10.9	58	84.8	0.212	4.6
23	793.8	1.986	9.4	59	80.5	0.201	4.5
24	778.9	1.948	6.8				
25	751.1	1.879	6.9				
26	738.6	1.848	7.4				
27	672.9	1.683	5.9				
28	658.8	1.648	11.5				
29	645.9	1.616	13.8				
30	632.6	1.582	14.3				
31	622.0	1.556	10.0				
32	618.9	1.548	9.1				
33	596.6	1.492	9.7				
34	587.6	1.470	8.1				
35	577.8	1.445	6.7				
36	564.1	1.411	6.1				

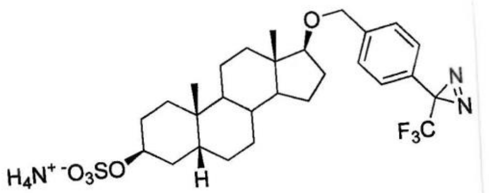

MQ235

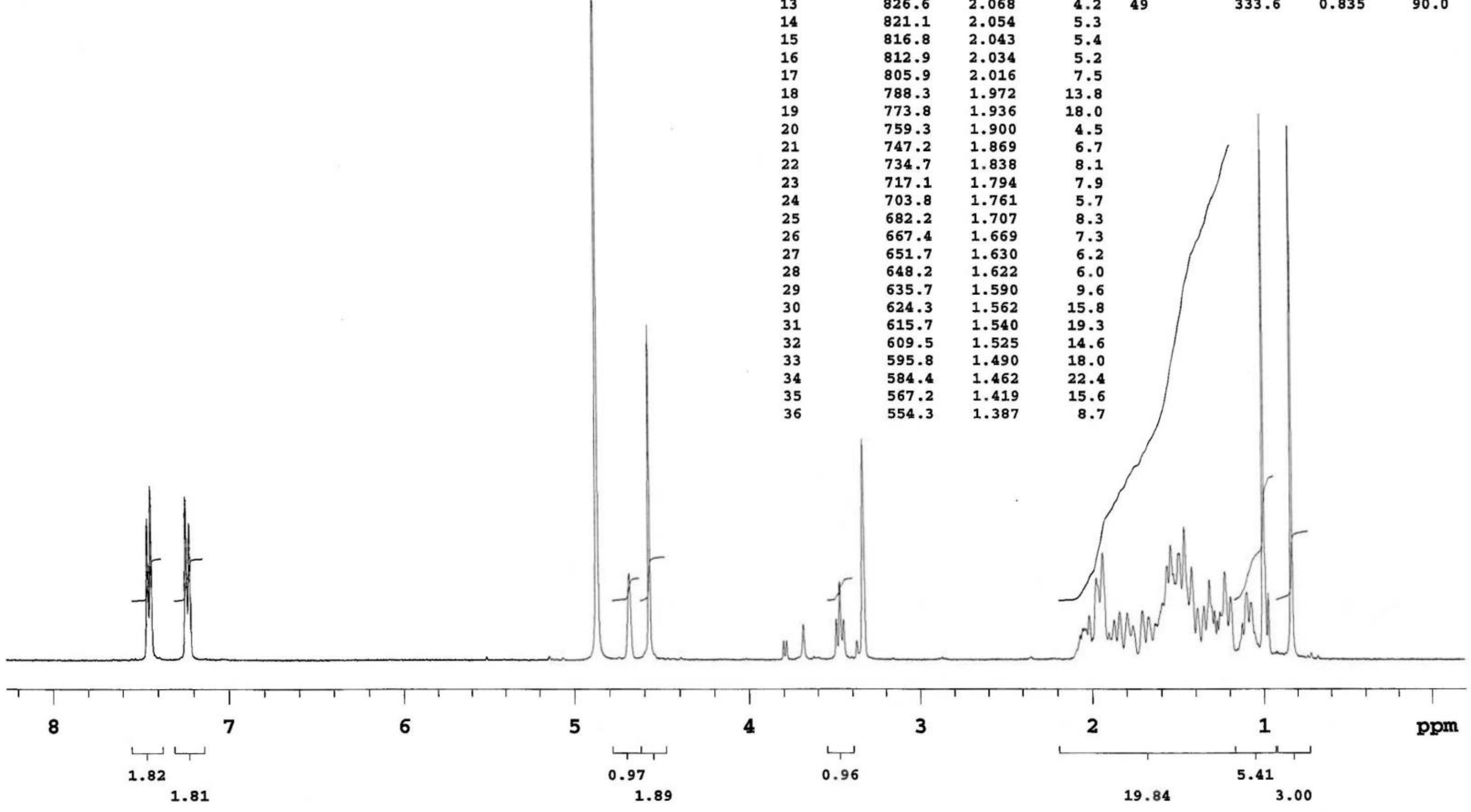

INDEX	FREQUENCY	PPM	HEIGHT	INDEX	FREQUENCY	PPM	HEIGHT
1	2981.2	7.458	23.8	37	538.7	1.347	9.1
2	2973.4	7.438	29.4	38	526.1	1.316	13.4
3	2894.8	7.242	27.5	39	514.4	1.287	8.2
4	2886.9	7.222	23.0	40	507.7	1.270	6.6
5	1946.8	4.870	150.3	41	501.5	1.255	8.0
6	1872.8	4.685	14.6	42	490.1	1.226	14.8
7	1827.4	4.571	56.6	43	477.6	1.195	10.6
8	1472.6	3.684	5.9	44	450.2	1.126	6.1
9	1395.1	3.490	6.9	45	438.9	1.098	11.4
10	1386.9	3.469	13.1	46	429.5	1.074	9.6
11	1378.7	3.449	6.8	47	399.8	1.000	92.1
12	1332.5	3.333	37.1	48	388.0	0.971	11.2
13	826.6	2.068	4.2	49	333.6	0.835	90.0
14	821.1	2.054	5.3				
15	816.8	2.043	5.4				
16	812.9	2.034	5.2				
17	805.9	2.016	7.5				
18	788.3	1.972	13.8				
19	773.8	1.936	18.0				
20	759.3	1.900	4.5				
21	747.2	1.869	6.7				
22	734.7	1.838	8.1				
23	717.1	1.794	7.9				
24	703.8	1.761	5.7				
25	682.2	1.707	8.3				
26	667.4	1.669	7.3				
27	651.7	1.630	6.2				
28	648.2	1.622	6.0				
29	635.7	1.590	9.6				
30	624.3	1.562	15.8				
31	615.7	1.540	19.3				
32	609.5	1.525	14.6				
33	595.8	1.490	18.0				
34	584.4	1.462	22.4				
35	567.2	1.419	15.6				
36	554.3	1.387	8.7				

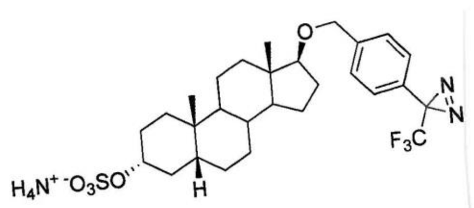

MQ236

INDEX	FREQUENCY	PPM	HEIGHT
1	2978.5	7.451	14.3
2	2970.3	7.430	17.1
3	2890.9	7.232	15.6
4	2882.6	7.211	13.0
5	2019.9	5.053	2.9
6	1946.8	4.870	150.3
7	1834.9	4.590	1.6
8	1824.3	4.564	35.3
9	1726.5	4.319	2.0
10	1720.2	4.303	2.8
11	1715.1	4.291	4.1
12	1710.5	4.279	2.8
13	1704.2	4.263	2.2
14	1513.3	3.786	6.8
15	1507.0	3.770	6.6
16	1479.6	3.701	2.4
17	1398.6	3.499	4.3
18	1390.4	3.478	7.9
19	1382.2	3.458	4.2
20	1341.1	3.355	1.7
21	1327.4	3.321	20.9
22	825.8	2.066	2.3
23	820.4	2.052	2.8
24	816.4	2.042	2.9
25	813.3	2.035	2.7
26	806.7	2.018	2.7
27	797.7	1.995	1.6
28	781.6	1.955	6.1
29	777.3	1.945	6.7
30	770.7	1.928	10.1
31	766.8	1.918	8.8
32	763.2	1.909	11.3
33	758.1	1.897	6.6
34	751.5	1.880	12.1
35	738.6	1.848	5.7
36	735.8	1.841	5.1

INDEX	FREQUENCY	PPM	HEIGHT
37	708.8	1.773	4.2
38	695.9	1.741	2.8
39	659.9	1.651	1.7
40	650.6	1.627	3.2
41	647.4	1.620	3.1
42	643.1	1.609	3.4
43	632.2	1.581	3.9
44	625.1	1.564	2.2
45	621.6	1.555	3.3
46	609.1	1.524	3.9
47	600.1	1.501	6.7
48	584.0	1.461	17.4
49	521.4	1.304	11.2
50	517.5	1.295	12.3
51	509.7	1.275	8.6
52	503.1	1.258	6.4
53	488.6	1.222	4.3
54	476.8	1.193	2.0
55	473.7	1.185	1.9
56	450.2	1.126	4.5
57	440.5	1.102	5.3
58	428.7	1.072	5.7
59	416.6	1.042	5.0
60	414.2	1.036	4.5
61	402.5	1.007	2.7
62	399.4	0.999	2.6
63	389.6	0.975	28.8
64	386.8	0.968	60.6
65	382.9	0.958	25.2
66	363.0	0.908	1.6
67	327.4	0.819	56.2

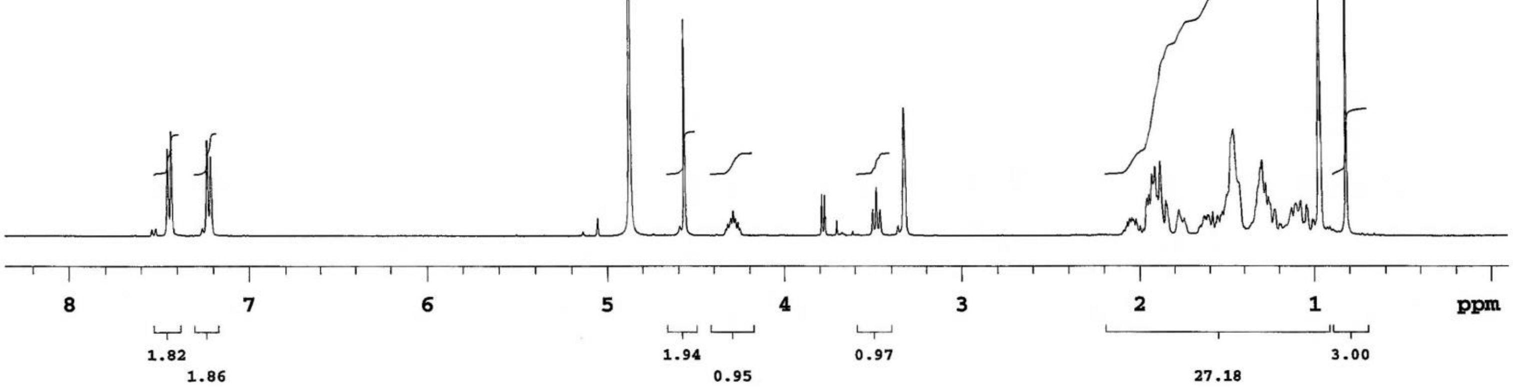

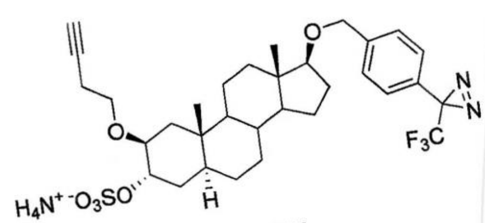

MQ237

INDEX	FREQUENCY	PPM	HEIGHT	INDEX	FREQUENCY	PPM	HEIGHT	INDEX	FREQUENCY	PPM	HEIGHT
1	3277.8	8.200	1.9	37	1338.4	3.348	6.0	73	525.4	1.314	16.9
2	2973.4	7.438	16.1	38	1336.8	3.344	4.8	74	514.8	1.288	30.6
3	2965.6	7.419	19.6	39	1324.3	3.313	39.2	75	502.7	1.257	15.3
4	2955.0	7.392	1.9	40	1323.1	3.310	44.3	76	495.2	1.239	13.1
5	2946.4	7.371	1.7	41	961.2	2.405	7.1	77	473.3	1.184	4.8
6	2947.2	7.373	1.7	42	955.4	2.390	14.3	78	469.8	1.175	4.7
7	2892.8	7.237	2.1	43	954.2	2.387	14.3	79	460.8	1.153	5.6
8	2887.0	7.222	18.4	44	949.9	2.376	9.7	80	457.3	1.144	6.0
9	2879.1	7.202	15.0	45	948.7	2.373	10.0	81	448.3	1.121	3.4
10	2194.8	5.491	3.8	46	937.0	2.344	2.8	82	412.3	1.031	3.4
11	2048.5	5.125	1.8	47	888.4	2.223	10.0	83	399.4	0.999	7.2
12	1947.2	4.871	191.0	48	886.1	2.217	15.2	84	393.1	0.983	6.4
13	1831.8	4.582	2.6	49	883.4	2.210	8.0	85	384.1	0.961	61.8
14	1818.5	4.549	38.8	50	814.5	2.038	3.3	86	371.2	0.929	10.3
15	1807.9	4.523	3.5	51	808.6	2.023	4.0	87	364.2	0.911	12.1
16	1795.0	4.490	9.8	52	804.7	2.013	4.2	88	359.5	0.899	8.4
17	1604.8	4.015	3.0	53	795.3	1.990	3.5	89	353.2	0.884	6.1
18	1599.4	4.001	3.8	54	785.9	1.966	1.9	90	323.1	0.808	63.2
19	1556.3	3.893	5.6	55	773.8	1.936	5.5	91	285.9	0.715	3.5
20	1536.0	3.842	1.7	56	758.6	1.898	9.7	92	276.1	0.691	5.5
21	1501.9	3.757	8.2	57	744.1	1.861	6.7	93	266.4	0.666	3.1
22	1498.0	3.747	12.1	58	722.2	1.807	2.8				
23	1459.7	3.651	1.7	59	707.7	1.770	6.9				
24	1451.8	3.632	2.1	60	708.5	1.772	7.0				
25	1444.8	3.614	5.0	61	694.4	1.737	5.0				
26	1442.5	3.608	6.0	62	672.1	1.681	5.0				
27	1435.8	3.592	10.3	63	659.6	1.650	6.4				
28	1431.9	3.582	6.4	64	625.1	1.564	17.7				
29	1429.2	3.575	6.3	65	613.4	1.534	11.3				
30	1424.9	3.564	9.9	66	591.5	1.480	4.2				
31	1418.2	3.548	5.9	67	579.0	1.448	5.4				
32	1408.8	3.524	2.1	68	565.7	1.415	6.3				
33	1393.6	3.486	1.9	69	561.0	1.403	4.3				
34	1381.4	3.456	5.9	70	557.5	1.395	5.3				
35	1373.6	3.436	17.7	71	550.4	1.377	5.9				
36	1365.4	3.416	5.2	72	535.9	1.341	8.2				

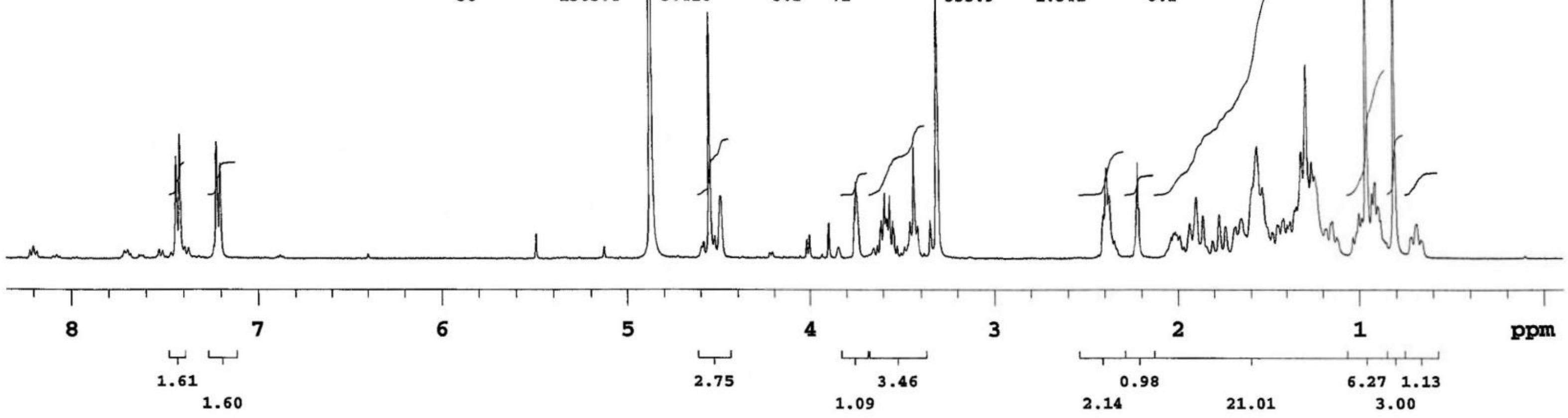

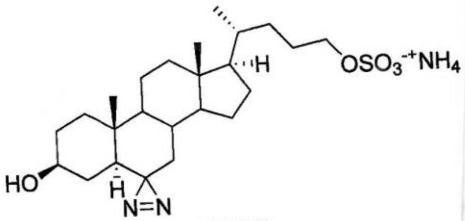

MQ271

INDEX	FREQUENCY	PPM	HEIGHT	INDEX	FREQUENCY	PPM	HEIGHT	INDEX	FREQUENCY	PPM	HEIGHT
1	1432.0	4.776	118.6	35	453.4	1.512	11.3	69	231.4	0.772	4.7
2	1172.6	3.910	14.4	36	446.9	1.490	9.1	70	203.3	0.678	108.3
3	1166.2	3.889	25.4	37	435.8	1.453	23.0	71	195.1	0.651	4.9
4	1160.3	3.869	12.3	38	426.4	1.422	17.4	72	190.4	0.635	6.0
5	1086.5	3.623	53.4	39	422.9	1.410	25.8	73	178.1	0.594	7.0
6	1008.6	3.364	4.3	40	413.6	1.379	18.5	74	110.2	0.367	5.0
7	1002.2	3.342	5.4	41	410.0	1.367	22.5	75	97.3	0.324	17.7
8	997.5	3.326	8.4	42	405.4	1.352	10.6	76	93.2	0.311	10.0
9	992.8	3.311	8.1	43	401.3	1.338	8.5	77	83.8	0.280	13.7
10	984.0	3.282	169.1	44	397.7	1.326	6.2	78	79.1	0.264	8.6
11	975.8	3.254	24.8	45	389.0	1.297	4.7				
12	974.0	3.248	37.0	46	375.5	1.252	10.6				
13	972.3	3.242	38.3	47	366.7	1.223	12.3				
14	971.1	3.239	24.6	48	364.4	1.215	12.6				
15	602.7	2.010	7.4	49	358.5	1.196	8.1				
16	599.2	1.998	4.8	50	353.8	1.180	10.4				
17	590.4	1.969	7.8	51	345.6	1.153	8.9				
18	547.1	1.824	3.7	52	340.9	1.137	8.5				
19	541.2	1.805	4.9	53	336.2	1.121	8.9				
20	537.1	1.791	6.5	54	322.8	1.076	109.5				
21	531.9	1.774	6.7	55	317.5	1.059	21.3				
22	524.8	1.750	8.3	56	304.6	1.016	16.0				
23	521.3	1.739	8.5	57	299.3	0.998	14.1				
24	512.5	1.709	18.4	58	294.7	0.983	11.6				
25	509.0	1.698	20.7	59	290.0	0.967	11.8				
26	504.9	1.684	17.2	60	284.7	0.949	9.1				
27	501.4	1.672	21.0	61	278.8	0.930	8.9				
28	499.1	1.664	24.7	62	271.8	0.906	56.6				
29	495.5	1.653	20.9	63	265.4	0.885	49.1				
30	492.6	1.643	20.2	64	254.2	0.848	6.3				
31	487.9	1.627	11.6	65	249.6	0.832	5.4				
32	470.9	1.571	4.9	66	241.9	0.807	8.0				
33	468.0	1.561	5.2	67	239.6	0.799	8.5				
34	457.5	1.526	11.3	68	233.2	0.778	5.0				

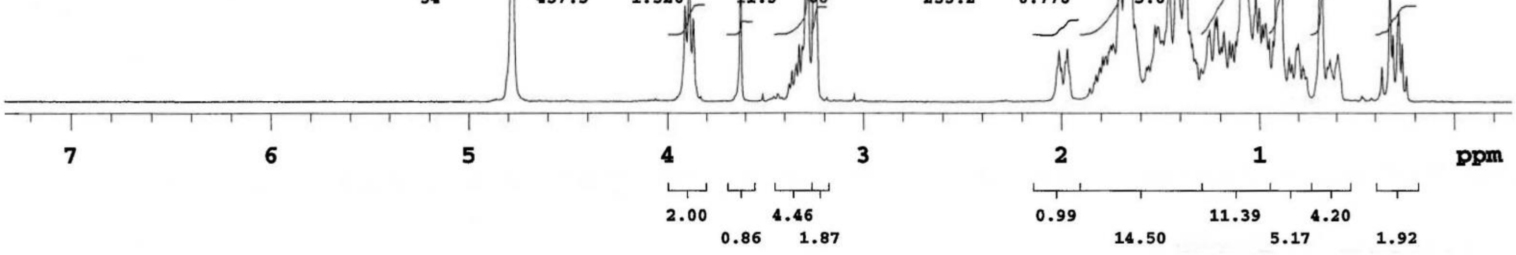

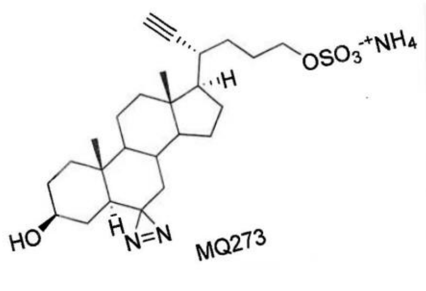

MQ273

INDEX	FREQUENCY	PPM	HEIGHT	INDEX	FREQUENCY	PPM	HEIGHT	INDEX	FREQUENCY	PPM	HEIGHT
1	1907.7	4.776	104.8	35	634.0	1.587	7.2	69	339.4	0.850	7.2
2	1579.9	3.955	21.1	36	627.4	1.571	8.8	70	330.8	0.828	11.7
3	1574.5	3.941	40.2	37	623.5	1.561	8.8	71	319.1	0.799	11.7
4	1568.3	3.926	22.2	38	612.6	1.534	11.0	72	293.4	0.735	122.6
5	1448.0	3.625	3.9	39	598.2	1.498	2.2	73	287.6	0.720	12.8
6	1345.2	3.367	5.9	40	585.8	1.466	13.0	74	253.4	0.634	9.9
7	1339.0	3.352	8.1	41	572.5	1.433	22.7	75	240.9	0.603	11.5
8	1333.9	3.339	11.6	42	559.7	1.401	25.7	76	142.4	0.357	6.9
9	1329.3	3.328	8.2	43	552.7	1.384	26.8	77	129.2	0.323	23.0
10	1323.0	3.312	6.6	44	542.6	1.358	28.1	78	118.3	0.296	23.6
11	1310.6	3.281	109.4	45	532.8	1.334	12.6	79	105.4	0.264	5.2
12	1295.4	3.243	10.6	46	514.5	1.288	12.6				
13	986.3	2.469	10.9	47	504.0	1.262	16.5				
14	973.5	2.437	11.5	48	493.1	1.234	15.8				
15	923.7	2.312	34.1	49	490.8	1.229	16.0				
16	907.7	2.272	2.5	50	484.2	1.212	11.6				
17	880.1	2.203	5.9	51	473.3	1.185	9.4				
18	869.9	2.178	12.1	52	460.4	1.153	9.2				
19	860.2	2.153	6.9	53	456.9	1.144	7.7				
20	758.2	1.898	7.7	54	446.8	1.119	12.4				
21	751.2	1.881	9.3	55	442.9	1.109	13.3				
22	745.0	1.865	9.1	56	434.4	1.087	132.3				
23	738.4	1.848	5.5	57	418.8	1.048	13.1				
24	731.4	1.831	4.8	58	414.5	1.038	12.8				
25	715.0	1.790	10.2	59	407.9	1.021	20.0				
26	707.6	1.771	15.0	60	396.6	0.993	12.4				
27	703.3	1.761	14.2	61	391.2	0.979	9.7				
28	697.1	1.745	12.5	62	385.3	0.965	5.9				
29	692.4	1.733	13.4	63	380.3	0.952	8.6				
30	683.5	1.711	22.8	64	373.6	0.935	8.5				
31	681.1	1.705	23.3	65	368.2	0.922	6.6				
32	670.6	1.679	37.8	66	362.0	0.906	6.0				
33	661.3	1.655	31.0	67	355.7	0.890	3.2				
34	639.5	1.601	8.5	68	342.9	0.858	7.0				

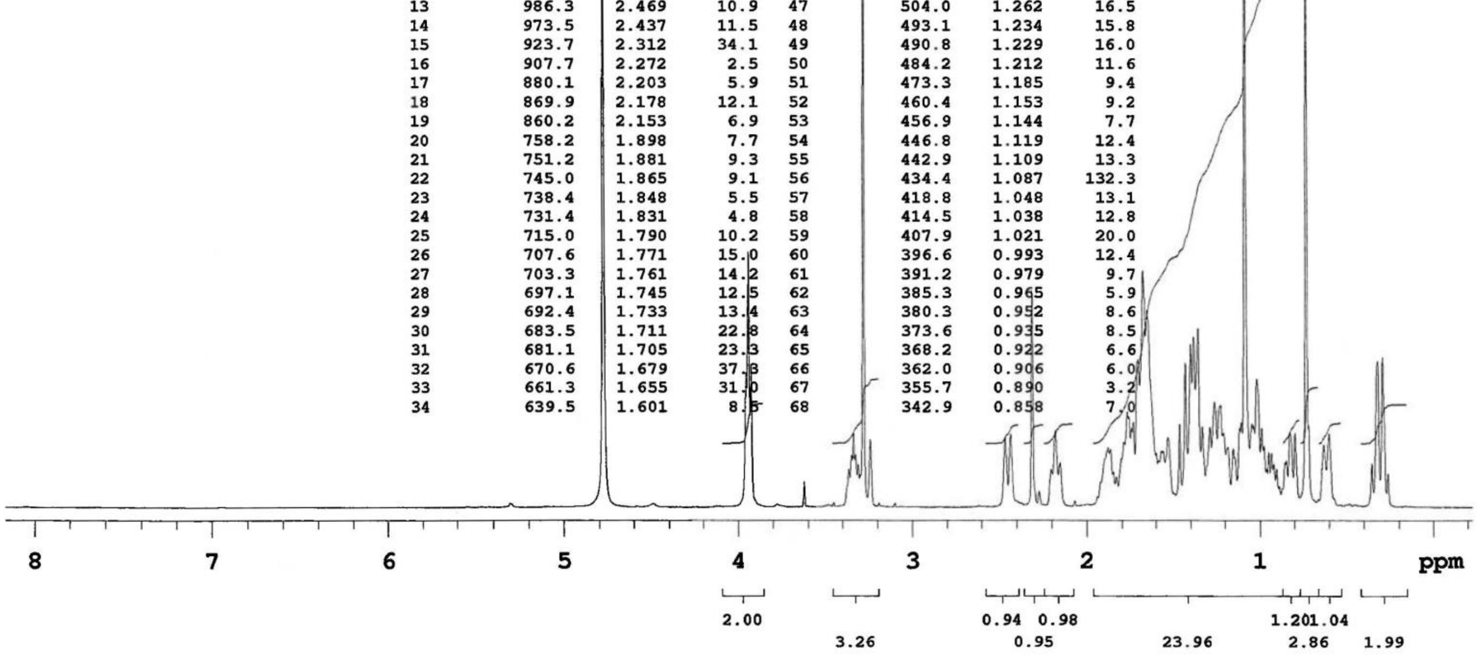

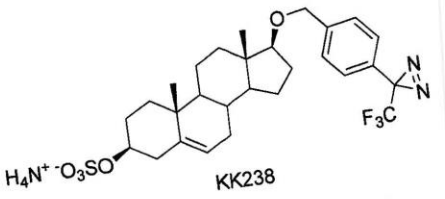

KK238

INDEX	FREQUENCY	PPM	HEIGHT
1	22309.9	222.105	-29.1
2	17246.3	171.694	16.1
3	14388.3	143.242	25.2
4	14234.9	141.715	28.2
5	12964.6	129.069	86.5
6	12951.7	128.940	18.2
7	12815.9	127.588	41.4
8	12381.0	123.258	35.1
9	9066.8	90.264	37.5
10	8036.8	80.010	32.8
11	7235.0	72.027	40.9
12	5307.8	52.841	38.0
13	5207.1	51.839	34.5
14	5001.1	49.788	131.5
15	4979.7	49.575	413.7
16	4958.4	49.363	818.7
17	4937.0	49.150	952.3
18	4915.6	48.937	772.3
19	4894.3	48.725	368.0
20	4872.9	48.512	110.6
21	4438.8	44.190	39.2
22	4068.8	40.506	32.4
23	3919.2	39.018	34.7
24	3875.7	38.585	35.6
25	3801.0	37.840	41.5
26	3331.0	33.162	38.2
27	3285.2	32.706	35.9
28	3023.5	30.101	31.8
29	2908.3	28.954	37.0
30	2468.1	24.571	33.3
31	2206.4	21.966	34.2
32	2003.5	19.946	52.1
33	1233.7	12.282	38.0

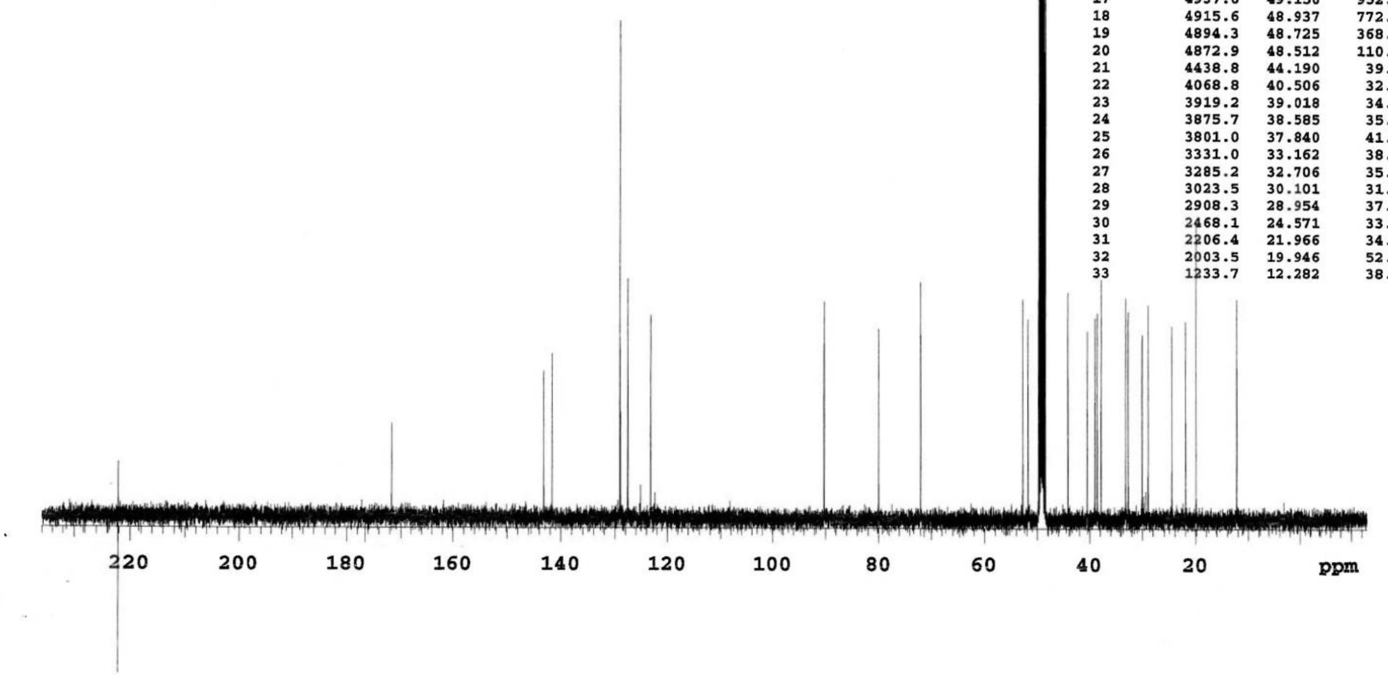

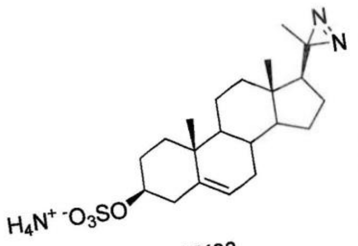

YX33

INDEX	FREQUENCY	PPM	HEIGHT
1	14080.4	140.177	33.4
2	12223.4	121.690	51.3
3	7869.3	78.343	53.9
4	5599.6	55.746	55.6
5	5381.4	53.574	51.3
6	5035.8	50.133	52.6
7	4844.3	48.227	205.2
8	4822.9	48.014	600.0
9	4801.6	47.802	1166.8
10	4780.2	47.589	1299.0
11	4758.8	47.376	1052.2
12	4737.5	47.164	492.9
13	4715.4	46.943	170.8
14	4382.0	43.624	45.6
15	3912.0	38.945	55.4
16	3866.2	38.490	57.2
17	3719.0	37.024	55.4
18	3641.9	36.257	39.9
19	3158.2	31.441	54.4
20	3150.6	31.365	48.5
21	2868.3	28.555	48.2
22	2544.0	25.327	27.7
23	2371.6	23.610	49.0
24	2312.9	23.026	50.8
25	2047.4	20.382	55.6
26	2019.9	20.109	40.9
27	1842.9	18.347	52.1
28	1223.4	12.179	64.5

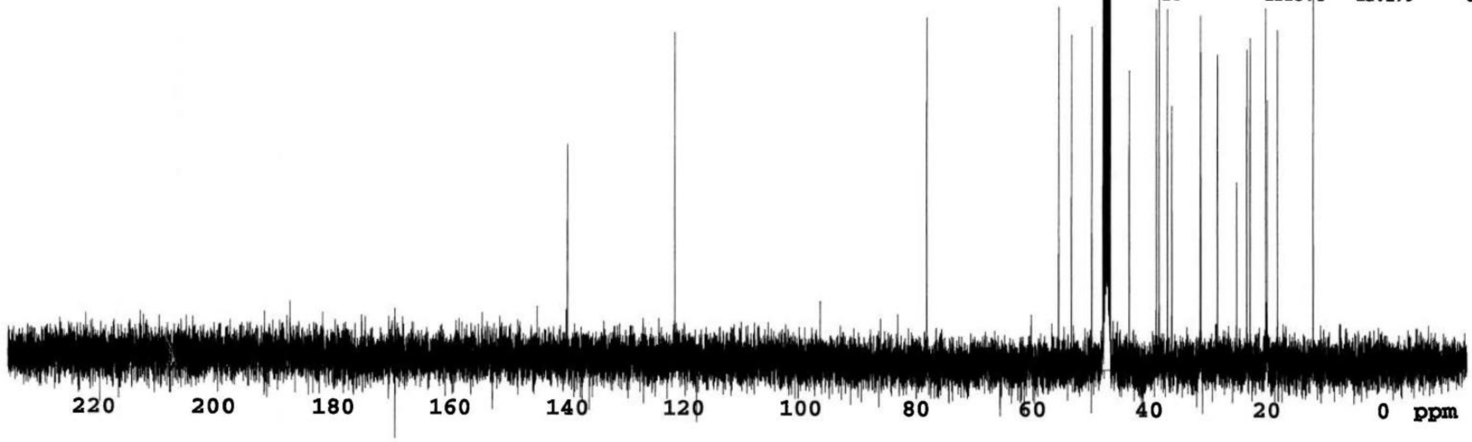

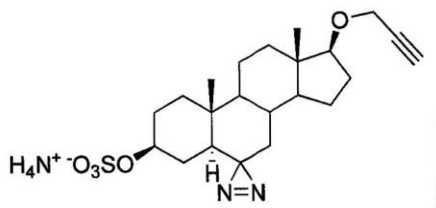

MQ189

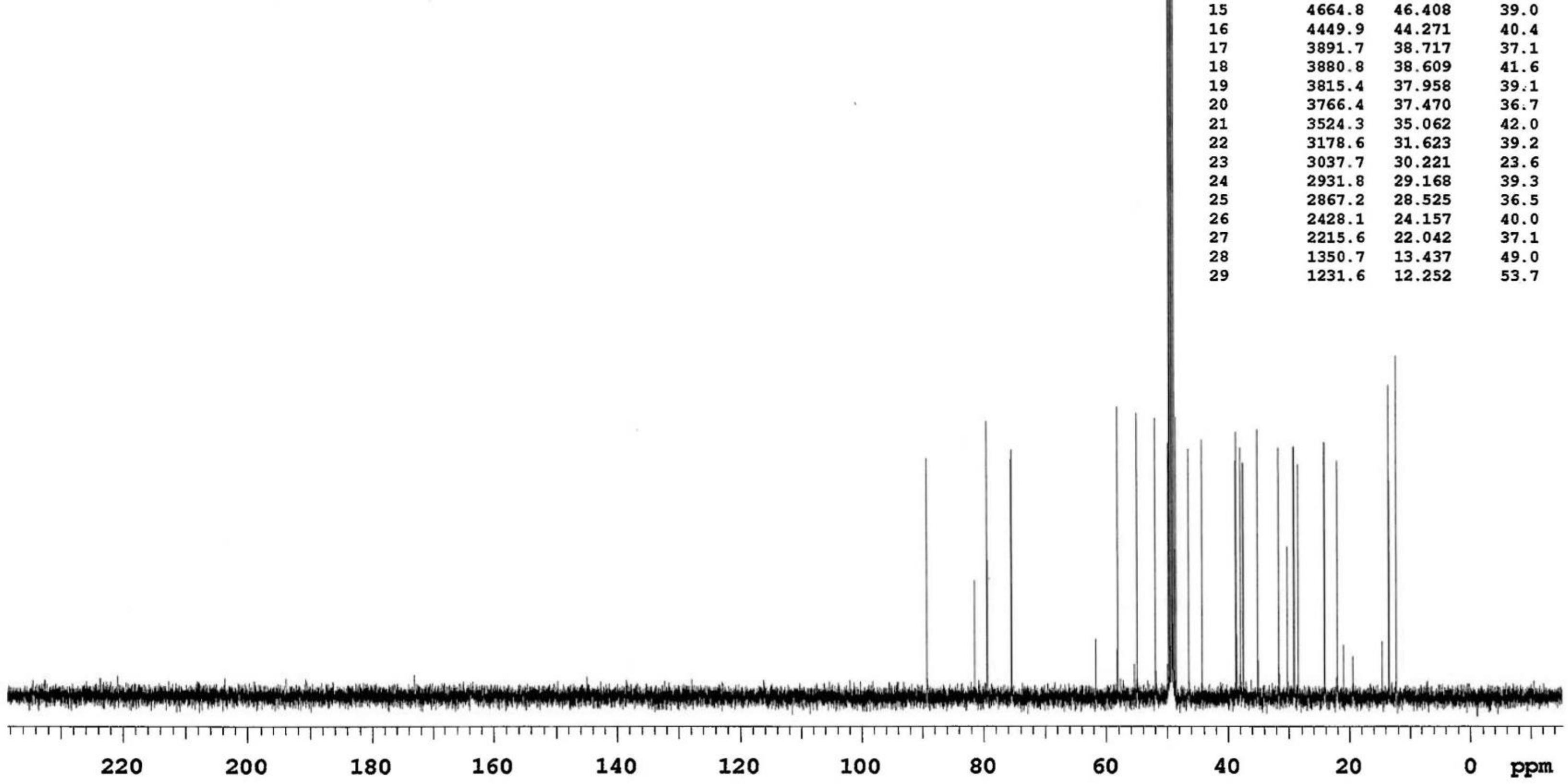

INDEX	FREQUENCY	PPM	HEIGHT
1	8967.6	89.215	37.5
2	8186.0	81.439	18.2
3	7982.8	79.418	43.3
4	7581.1	75.421	38.8
5	5841.9	58.119	45.6
6	5526.6	54.982	44.6
7	5220.6	51.938	43.8
8	5004.2	49.785	39.9
9	4983.2	49.576	144.6
10	4961.4	49.359	249.9
11	4940.4	49.150	338.9
12	4918.6	48.933	256.6
13	4897.6	48.724	142.8
14	4875.8	48.507	43.9
15	4664.8	46.408	39.0
16	4449.9	44.271	40.4
17	3891.7	38.717	37.1
18	3880.8	38.609	41.6
19	3815.4	37.958	39.1
20	3766.4	37.470	36.7
21	3524.3	35.062	42.0
22	3178.6	31.623	39.2
23	3037.7	30.221	23.6
24	2931.8	29.168	39.3
25	2867.2	28.525	36.5
26	2428.1	24.157	40.0
27	2215.6	22.042	37.1
28	1350.7	13.437	49.0
29	1231.6	12.252	53.7

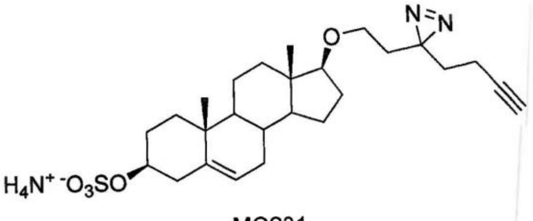

MQ231

INDEX	FREQUENCY	PPM	HEIGHT
1	14241.2	141.681	35.5
2	12381.4	123.178	15.5
3	12377.5	123.139	16.5
4	9123.3	90.764	19.3
5	8427.3	83.840	10.9
6	8029.5	79.883	27.8
7	8003.8	79.627	16.0
8	7971.1	79.302	16.5
9	7938.4	78.976	16.3
10	7054.0	70.178	11.2
11	6604.8	65.709	22.6
12	5307.1	52.798	36.8
13	5205.8	51.791	41.3
14	5005.0	49.793	135.0
15	4983.2	49.576	441.5
16	4962.2	49.367	808.8
17	4940.4	49.150	1058.3
18	4919.4	48.941	799.3
19	4897.6	48.724	464.6
20	4876.5	48.515	131.2
21	4422.7	44.000	38.9
22	4066.1	40.452	23.1
23	3918.2	38.981	35.5
24	3873.8	38.539	40.6
25	3799.1	37.796	43.8
26	3478.3	34.605	46.3
27	3440.2	34.225	56.8
28	3328.1	33.110	49.3
29	3286.0	32.692	37.4
30	3019.8	30.043	33.3
31	2899.9	28.850	34.6
32	2829.8	28.153	13.5
33	2465.5	24.528	34.7
34	2203.1	21.918	42.5
35	2004.6	19.943	28.1
36	1410.6	14.034	28.4
37	1230.8	12.245	30.7

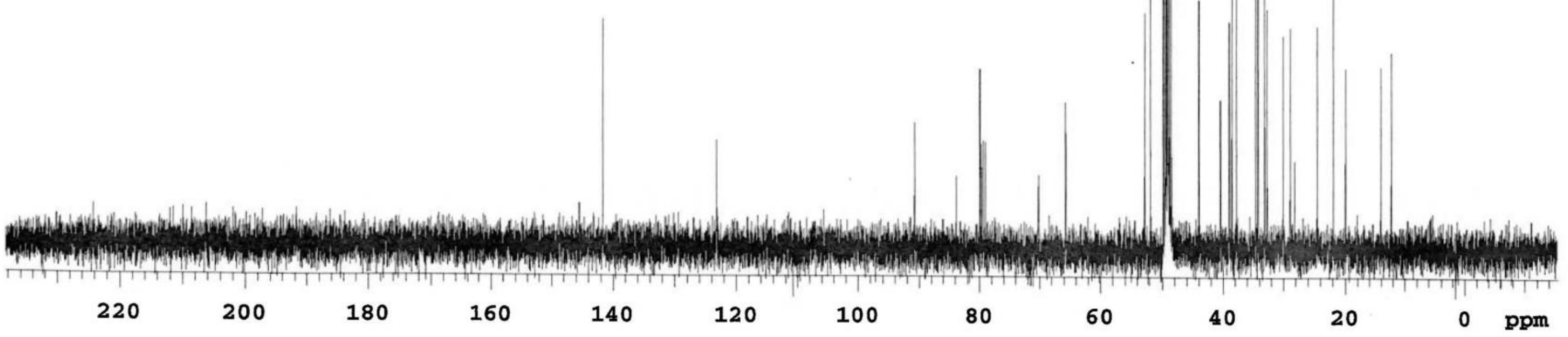

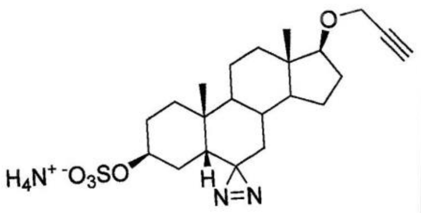

MQ234

INDEX	FREQUENCY	PPM	HEIGHT
1	8979.3	89.332	12.1
2	8188.3	81.463	14.5
3	7581.1	75.421	16.7
4	7576.4	75.375	18.2
5	7560.8	75.220	20.5
6	7554.6	75.158	18.6
7	5844.2	58.142	28.7
8	5236.2	52.093	46.9
9	5004.2	49.785	148.3
10	4983.2	49.576	444.8
11	4961.4	49.359	909.3
12	4940.4	49.150	1028.4
13	4918.6	48.933	927.6
14	4897.6	48.724	435.1
15	4875.8	48.507	157.2
16	4576.0	45.525	54.5
17	4460.0	44.371	37.5
18	4096.5	40.754	62.9
19	3893.3	38.733	54.2
20	3800.6	37.811	50.6
21	3537.5	35.193	58.8
22	3390.4	33.729	47.0
23	3090.6	30.748	42.9
24	3026.0	30.105	34.2
25	2878.9	28.641	43.1
26	2809.6	27.952	30.3
27	2630.5	26.170	54.8
28	2429.7	24.172	45.7
29	2426.6	24.141	31.0
30	2191.5	21.802	49.9
31	1226.9	12.206	27.0

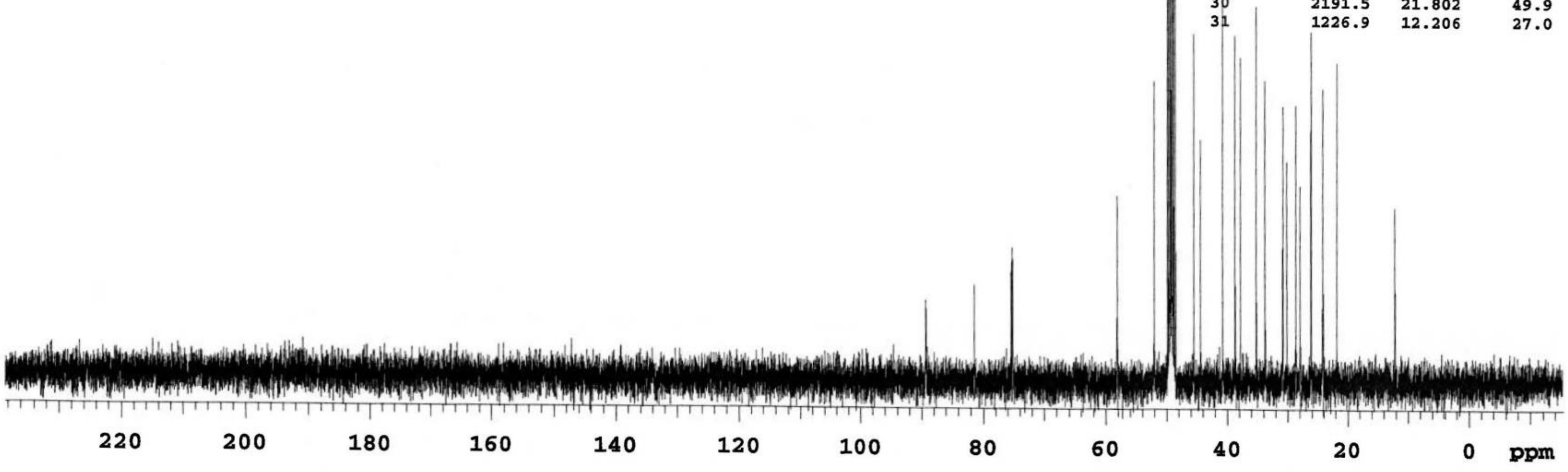

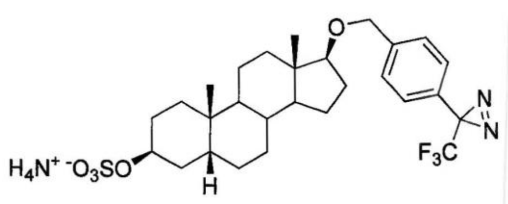

MQ235

INDEX	FREQUENCY	PPM	HEIGHT
1	14403.2	143.292	19.0
2	12973.0	129.064	39.4
3	12959.8	128.932	15.6
4	12825.1	127.592	31.7
5	9088.3	90.416	16.5
6	7762.5	77.226	14.3
7	7759.4	77.195	13.8
8	7238.5	72.014	16.4
9	5286.0	52.589	32.5
10	5005.0	49.793	79.9
11	4983.2	49.576	235.4
12	4962.2	49.367	465.3
13	4940.4	49.150	560.7
14	4919.4	48.941	458.7
15	4897.6	48.724	243.5
16	4876.5	48.515	74.7
17	4480.3	44.573	32.6
18	4180.6	41.591	33.5
19	3961.0	39.407	26.4
20	3867.6	38.477	30.4
21	3723.6	37.044	33.0
22	3622.4	36.038	34.0
23	3274.4	32.575	29.9
24	3181.7	31.654	25.9
25	2920.9	29.059	26.7
26	2781.6	27.673	26.4
27	2718.5	27.045	27.6
28	2699.8	26.860	27.5
29	2467.8	24.552	30.8
30	2460.8	24.482	25.5
31	2214.0	22.027	28.1
32	1249.5	12.430	28.3

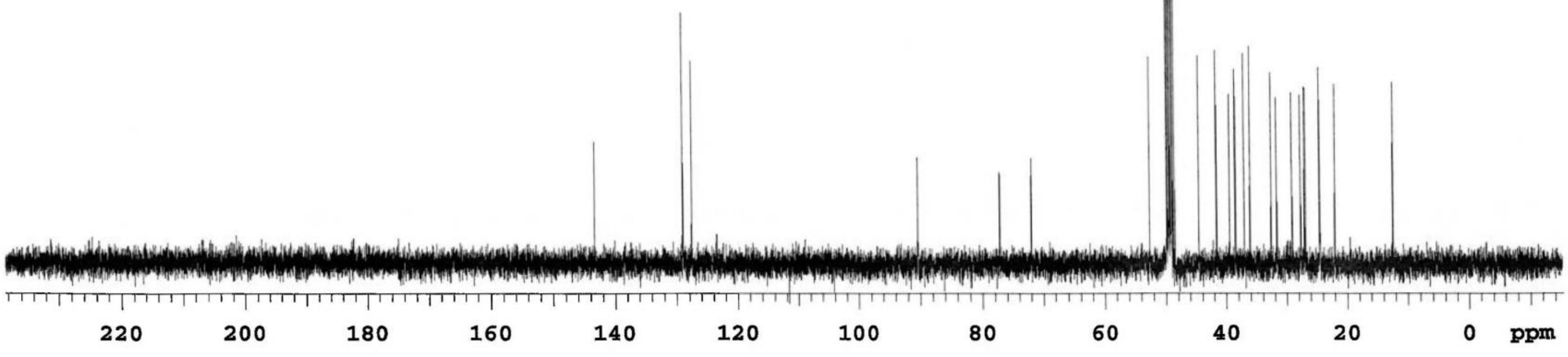

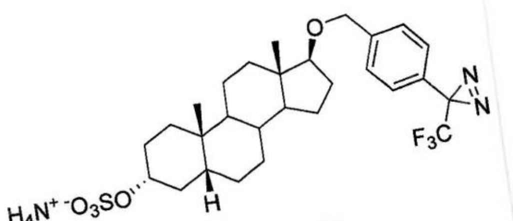

MQ236

INDEX	FREQUENCY	PPM	HEIGHT
1	14403.9	143.300	12.6
2	12972.3	129.056	49.6
3	12958.2	128.917	10.1
4	12824.3	127.585	26.1
5	9090.6	90.439	24.6
6	8091.0	80.494	25.3
7	7574.9	75.359	6.4
8	7242.4	72.052	24.0
9	5281.4	52.542	23.1
10	5005.0	49.793	61.1
11	4983.2	49.576	230.5
12	4962.2	49.367	355.8
13	4940.4	49.150	540.3
14	4919.4	48.941	343.5
15	4897.6	48.724	232.4
16	4875.8	48.507	55.3
17	4477.9	44.549	25.9
18	4403.2	43.806	22.8
19	4233.5	42.117	27.3
20	3955.6	39.352	21.7
21	3735.2	37.161	27.2
22	3677.6	36.587	20.5
23	3598.2	35.797	27.9
24	3486.9	34.690	19.3
25	2920.9	29.059	17.7
26	2912.4	28.974	19.7
27	2840.0	28.254	18.6
28	2738.8	27.247	19.5
29	2467.8	24.552	23.4
30	2410.2	23.978	27.3
31	2185.2	21.740	21.1
32	1959.5	19.494	20.5
33	1247.9	12.415	34.5

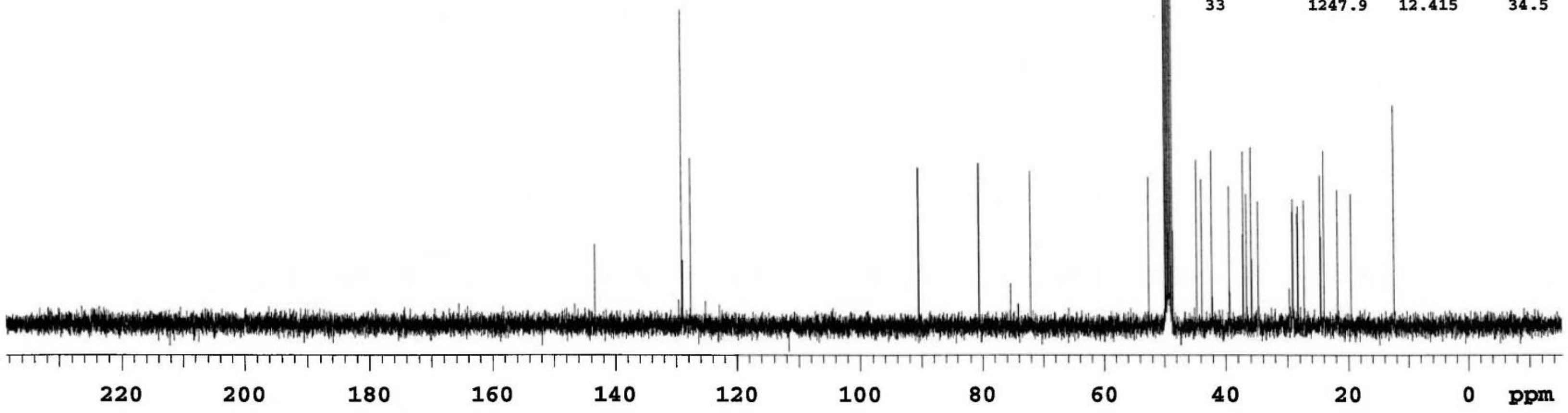

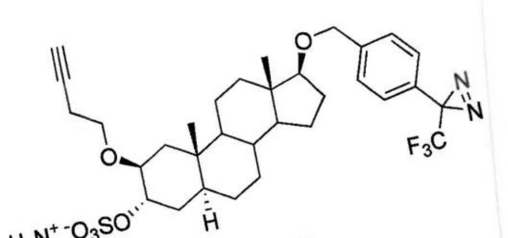

MQ237

INDEX	FREQUENCY	PPM	HEIGHT
1	14245.6	141.724	20.1
2	12814.7	127.489	59.2
3	12800.7	127.349	16.1
4	12666.0	126.009	35.0
5	8931.5	88.856	27.5
6	8128.1	80.863	15.6
7	7705.3	76.658	24.6
8	7494.4	74.559	17.6
9	7081.0	70.446	25.7
10	6916.7	68.812	25.5
11	6763.3	67.286	26.1
12	5544.2	55.157	30.7
13	5130.8	51.045	32.5
14	4845.9	48.210	155.3
15	4824.1	47.993	387.3
16	4803.1	47.784	948.3
17	4781.3	47.567	919.7
18	4760.2	47.358	942.0
19	4738.4	47.141	404.8
20	4717.4	46.932	155.5
21	4314.9	42.928	33.9
22	3940.5	39.202	31.9
23	3791.8	37.723	24.2
24	3710.8	36.917	23.3
25	3527.9	35.097	34.4
26	3488.9	34.710	30.6
27	3148.7	31.325	22.8
28	2951.8	29.366	27.1
29	2775.8	27.616	21.1
30	2753.2	27.391	21.7
31	2304.8	22.930	23.5
32	2030.8	20.204	22.8
33	1958.4	19.483	32.8
34	1264.7	12.582	35.4
35	1096.6	10.909	41.0

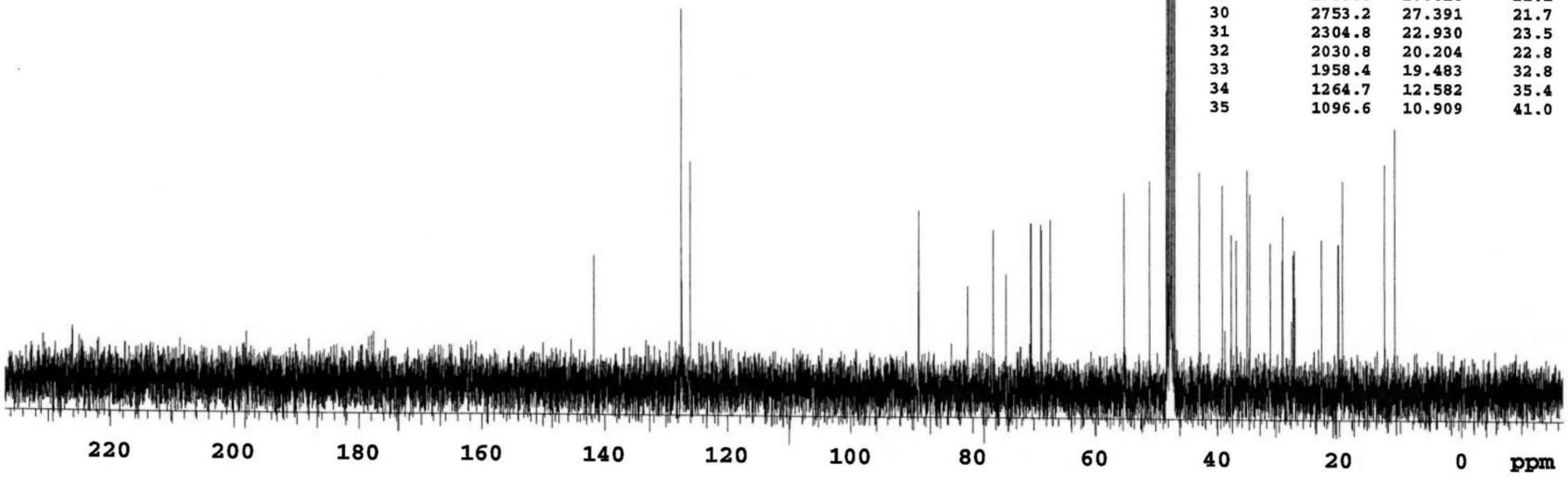

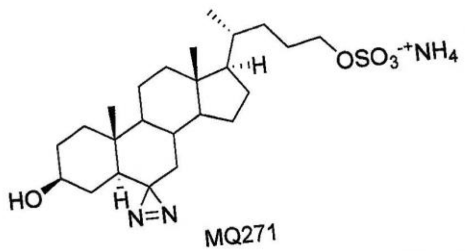

MQ271

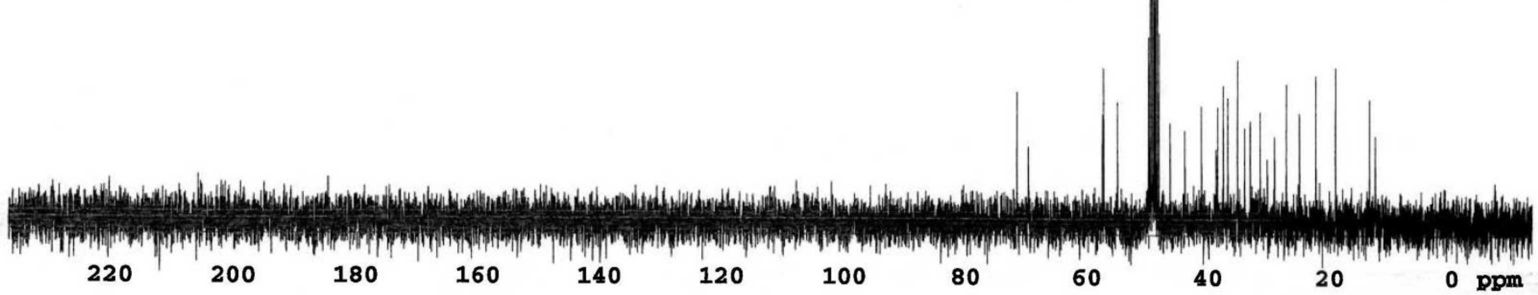

INDEX	FREQUENCY	PPM	HEIGHT
1	5402.8	71.655	19.6
2	5260.1	69.763	11.4
3	4340.9	57.571	16.2
4	4328.2	57.403	23.3
5	4154.5	55.099	18.0
6	3769.1	49.987	28.0
7	3748.3	49.713	83.3
8	3726.5	49.423	174.5
9	3705.8	49.148	156.2
10	3683.9	48.858	183.9
11	3662.1	48.568	71.1
12	3641.3	48.294	28.5
13	3505.6	46.493	14.9
14	3316.9	43.991	13.8
15	3108.7	41.229	17.5
16	2925.7	38.803	11.0
17	2906.2	38.543	17.3
18	2833.7	37.582	20.6
19	2779.6	36.865	18.8
20	2661.1	35.293	24.6
21	2571.4	34.103	14.2
22	2503.5	33.203	15.3
23	2378.1	31.539	16.6
24	2203.2	29.220	12.9
25	2052.5	27.221	20.9
26	1890.3	25.070	16.5
27	1692.4	22.445	22.2
28	1447.3	19.195	23.5
29	1018.2	13.503	18.6
30	950.3	12.603	13.0

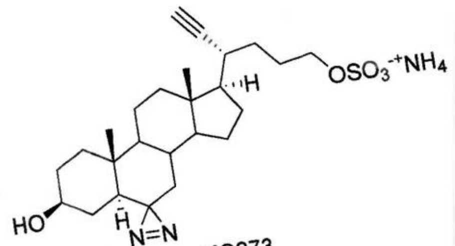

MQ273

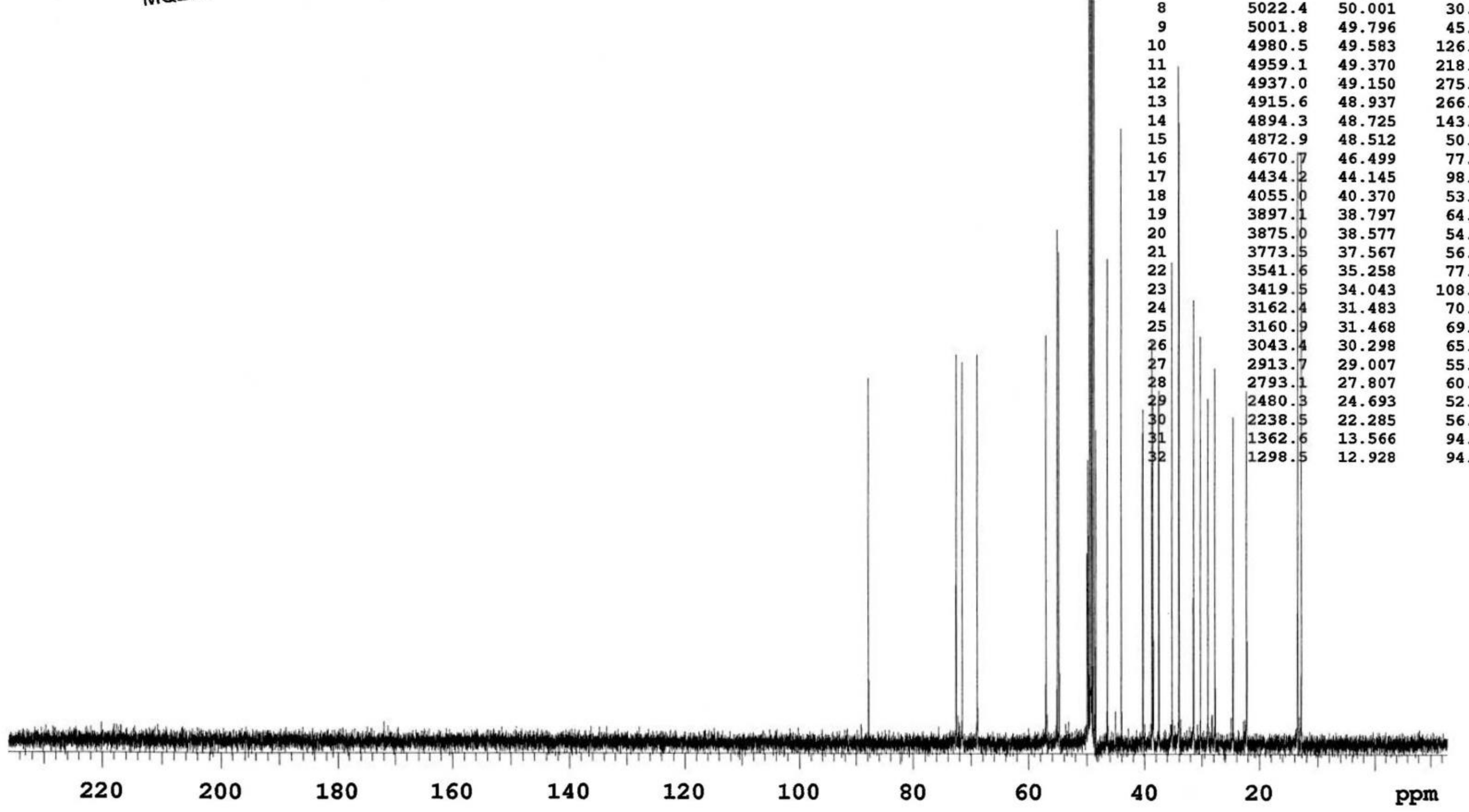

INDEX	FREQUENCY	PPM	HEIGHT
1	8831.0	87.917	58.2
2	7292.2	72.597	62.0
3	7188.4	71.564	60.8
4	6932.8	69.020	62.1
5	5735.8	57.102	65.1
6	5544.3	55.196	82.1
7	5512.2	54.877	78.5
8	5022.4	50.001	30.0
9	5001.8	49.796	45.0
10	4980.5	49.583	126.5
11	4959.1	49.370	218.7
12	4937.0	49.150	275.8
13	4915.6	48.937	266.4
14	4894.3	48.725	143.4
15	4872.9	48.512	50.0
16	4670.7	46.499	77.5
17	4434.2	44.145	98.4
18	4055.0	40.370	53.3
19	3897.1	38.797	64.3
20	3875.0	38.577	54.9
21	3773.5	37.567	56.3
22	3541.6	35.258	77.0
23	3419.5	34.043	108.4
24	3162.4	31.483	70.9
25	3160.9	31.468	69.8
26	3043.4	30.298	65.0
27	2913.7	29.007	55.1
28	2793.1	27.807	60.0
29	2480.3	24.693	52.1
30	2238.5	22.285	56.2
31	1362.6	13.566	94.7
32	1298.5	12.928	94.3
